# The alanyl-tRNA synthetase AARS1 moonlights as a lactyltransferase to promote YAP signaling in gastric cancer

**DOI:** 10.1172/JCI174587

**Published:** 2024-03-21

**Authors:** Junyi Ju, Hui Zhang, Moubin Lin, Zifeng Yan, Liwei An, Zhifa Cao, Dandan Geng, Jingwu Yue, Yang Tang, Luyang Tian, Fan Chen, Yi Han, Wenjia Wang, Shimin Zhao, Shi Jiao, Zhaocai Zhou

**Affiliations:** 1 Department of Medical Ultrasound and Department of Stomatology, Shanghai Tenth People’s Hospital, Tongji University Cancer Center, Tongji University School of Medicine, Shanghai, China.; 2 State Key Laboratory of Genetic Engineering, School of Life Sciences, Zhongshan Hospital, Fudan University, Shanghai, China.; 3 Department of General Surgery, Yangpu Hospital, Tongji University School of Medicine, Shanghai, China.; 4 School of Life Science, Inner Mongolia University, Hohhot, Inner Mongolia, China.; 5 School of Medicine, Anhui University of Science and Technology, Huainan, China.; 6 Collaborative Innovation Center for Cancer Personalized Medicine, School of Public Health, Nanjing Medical University, Nanjing, China.

**Keywords:** Metabolism, Cancer

## Abstract

Lactylation has been recently identified as a new type of posttranslational modification occurring widely on lysine residues of both histone and nonhistone proteins. The acetyltransferase p300 is thought to mediate protein lactylation, yet the cellular concentration of the proposed lactyl-donor, lactyl-coenzyme A, is about 1,000 times lower than that of acetyl-CoA, raising the question of whether p300 is a genuine lactyltransferase. Here, we report that alanyl-tRNA synthetase 1 (AARS1) moonlights as a bona fide lactyltransferase that directly uses lactate and ATP to catalyze protein lactylation. Among the candidate substrates, we focused on the Hippo pathway, which has a well-established role in tumorigenesis. Specifically, AARS1 was found to sense intracellular lactate and translocate into the nucleus to lactylate and activate the YAP-TEAD complex; and AARS1 itself was identified as a Hippo target gene that forms a positive-feedback loop with YAP-TEAD to promote gastric cancer (GC) cell proliferation. Consistently, the expression of AARS1 was found to be upregulated in GC, and elevated AARS1 expression was found to be associated with poor prognosis for patients with GC. Collectively, this work found AARS1 with lactyltransferase activity in vitro and in vivo and revealed how the metabolite lactate is translated into a signal of cell proliferation.

## Abstract 2

The alanyl-tRNA synthetase AARS1 moonlights as a protein lactyltransferase.

## Graphical abstract

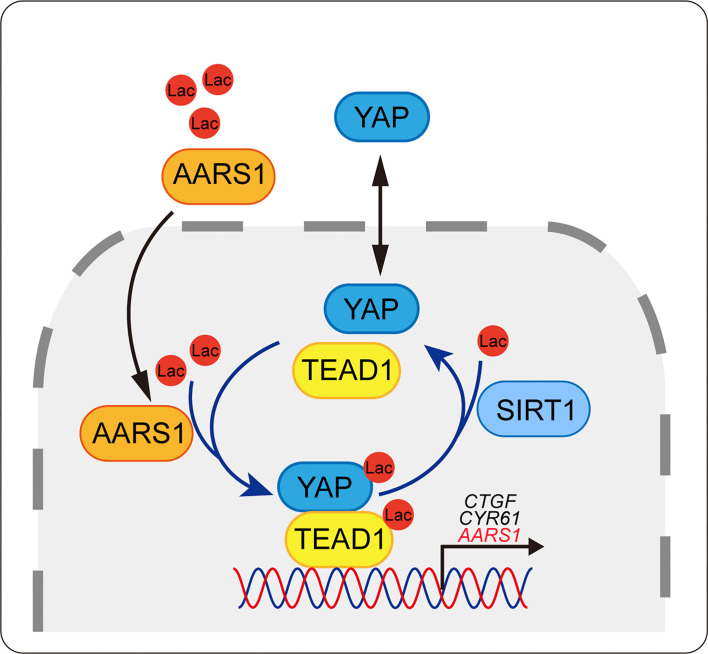

## Introduction

Lactate is a well-known metabolite found in almost all types of cells, and is highly abundant in proliferating tumor cells owing to the Warburg effect (1, 2). Despite many studies showing that tumor-secreted lactate can enter multiple types of immune cells to shape a microenvironment permissive for tumor growth (3–6), how intracellular lactate is manifested as or translated into signals beneficial for tumor growth remains elusive. Recently, lactylation of lysine residues has been identified as a new type of posttranslational modification for histones (7, 8), providing a new perspective for nonmetabolic functions of lactate. For example, histone lactylation has been found to play essential roles in stem cell pluripotency (9), neural excitation (10), Alzheimer’s disease (11), macrophage polarization (12), and tumor development (13). More recently, lactylation has also been found in nonhistone proteins (8). For example, an integrative lactylome and proteome analysis of hepatocellular carcinoma identified more than 9,000 lactylated lysines on nonhistone proteins (14).

Although protein lactylation has increasingly been appreciated as a widespread posttranslational modification especially in tumor cells, the enzyme that directly catalyzes this modification, as well as the exact chemical reaction process of catalysis, is still subject to debate. Specifically, the acetyltransferase p300 has been proposed to serve as a lactyltransferase and thus mediate histone lactylation (7), but direct in vitro evidence using purified proteins of p300 and substrate is lacking. More importantly, in that proposed catalysis system, p300 would need to use lactyl-coenzyme A (lactyl-CoA) as a lactyl-donor, but the enzymes responsible for producing lactyl-CoA in mammalian cells are still undefined, and the levels of lactyl-CoA in tumor cells are hardly detectable. In fact, the intracellular concentration of lactyl-CoA in general is at least 1,000 times lower than that of acetyl-CoA in mammalian cells (15), which may substantially limit p300’s lactyltransferase activity, if any, in vivo. Therefore, a genuine lactyltransferase that can directly use lactate as a lactyl-donor to catalyze substrate lactylation in vitro and in vivo is yet to be identified.

The evolutionally conserved Hippo signaling pathway plays an essential role in organ size control, tissue homeostasis, and tumorigenesis (16–18). In this pathway, the MST1/2 (Hippo)–LATS1/2 kinase cascade controls the subcellular localization and therefore activity of the transcriptional coactivator YAP/TAZ (19, 20). In response to various environmental stimuli, however, the upstream kinase cascade can be inactivated, allowing YAP/TAZ to enter the nucleus and interact with the TEAD family of transcription factors to regulate downstream target gene expression (21, 22). Dysregulation of the Hippo pathway — in particular, hyperactivation of YAP — has been shown to be closely associated with aberrant cell growth and tumorigenesis (23–25). It has been speculated that the Hippo pathway may also directly sense certain metabolic cues (26). However, the specific molecular machineries linking intracellular lactate to Hippo/YAP signaling, if any, are yet to be discovered, especially in tumor cells, in which lactate is highly abundant and YAP is hyperactive.

In this study, we identified alanyl-tRNA synthetase 1 (AARS1), which typically functions to catalyze the ligation of l-alanine to transfer RNA (tRNA), as a bona fide lactyltransferase that can directly use lactate and ATP to catalyze protein lactylation. Moreover, we found that in response to intracellular accumulation of lactate, AARS1 translocated into the nucleus, where it directly catalyzed lactylation of YAP at K90 and TEAD1 at K108, thereby activating downstream target gene expression to promote tumor cell proliferation. Furthermore, AARS1 was shown to be a direct target gene of YAP-TEAD1, forming a positive-feedback loop to manifest high levels of intracellular lactate as a growth signal. Consistently, we found AARS1 to be upregulated and associated with YAP-TEAD1 lactylation in gastric cancer (GC), and found that elevated expression of AARS1 was strongly associated with TNM stages and poor clinical outcomes.

## Results

### AARS1 moonlights as a protein lactyltransferase using lactate as a direct lactyl-donor.

**Figure 1:**
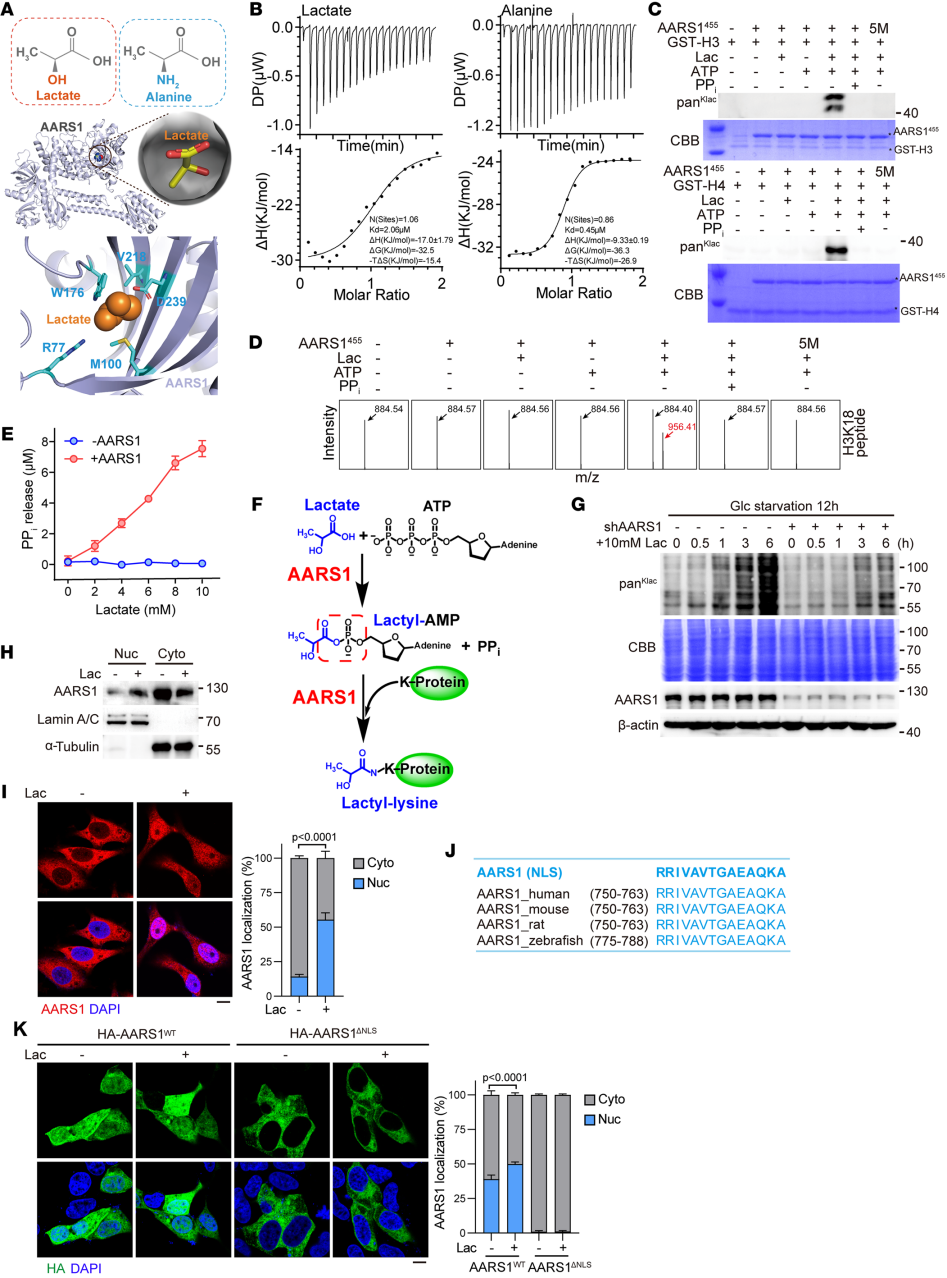
AARS1 is a lactyltransferase sensitive to intracellular lactate levels. (**A**) Top: Comparison of the chemical formula of lactate and l-alanine. Middle: A predicted overall structural view of lactate with AARS1. Bottom: Detailed interactions between lactate (orange) and amino acid residues in the catalytic core of AARS1 (cyan). (**B**) Isothermal titration calorimetry analysis of the interaction between lactate (left) or l-alanine (right) and AARS1. DP, differential power; ΔH, enthalpy; ΔG, Gibbs free energy; ΔS, entropy; T, temperature. (**C**) Immunoblotting with pan-Klac antibody to detect AARS1^455^-induced lactylation of GST-H3 and GST-H4 in vitro. Coomassie brilliant blue (CBB) staining showing the purified AARS1^455^, GST-H3, and GST-H4 used in in vitro lactylation assay. Asterisks represent the AARS1^455^, GST-H3, and GST-H4 proteins. Lac, lactate. (**D**) Mass spectrometry to determine the lactylation of the synthetic H3K18 peptide catalyzed by AARS1^455^ and its catalytic-dead mutant 5M in vitro. 5M: R77A, M100A, W176E, V218D, D239A. (**E**) PP_i_ production in in vitro lactylation assay in the absence and presence of AARS1 (*n* = 3). Data are presented as mean ± SD. (**F**) Schematic illustration showing the proposed catalytic mechanism of AARS1-induced protein lysine lactylation. (**G**) Immunoblotting with pan-Klac antibody to detect global protein lactylation levels in glucose-deprived AARS1-knockdown HGC27 cells stimulated with 10 mM lactate for indicated times. (**H**) Nucleocytoplasmic distribution of AARS1 in lactate-treated HGC27 cells. (**I**) Left: Immunofluorescence staining of AARS1 in lactate-treated HGC27 cells. Scale bar: 5 μm. Right: Statistical analysis of AARS1 cellular distribution (*n* = 10). Data are presented as mean ± SD. (**J**) Alignment of nuclear localization signal (NLS) sequences of AARS1 in the indicated species. (**K**) Left: Immunofluorescence staining of HA-AARS1 in lactate-treated HEK293A cells after transfection with HA-tagged AARS1 or its NLS-deletion (ΔNLS) mutant. Scale bar: 5 μm. Right: Statistical analysis of HA-AARS1 cellular localization (*n* = 10). Data are presented as mean ± SD. Unpaired 2-tailed Student’s *t* test (**I** and **K**).

To find a lactyltransferase that can directly use lactate and ATP to catalyze protein lactylation, we reasoned that the candidate enzyme should readily bind both lactate and ATP, as well as a protein substrate. Considering the high similarity between the chemical structure of lactate and that of l-alanine (Figure 1A), we speculated that alanyl-tRNA synthetase 1/2 (AARS1/2) may also act as a lactyltransferase to catalyze protein lactylation, a scenario reminiscent of AARS1/2 catalyzing the ligation of l-alanine to tRNA. Supporting this idea, molecular docking predicted that lactate could readily bind to the catalytic pocket of AARS1 (Figure 1A). To verify whether AARS1 may directly bind lactate and thus use it as a lactyl-donor, we used purified recombinant protein of AARS1 to examine its interaction with lactate by isothermal titration calorimetry assay. Indeed, lactate was found to bind AARS1 with a *K_D_* value of 2.06 μM, as did the positive control l-alanine (*K_D_* = 0.45 μM) (Figure 1B).

To further test whether AARS1 is a lactyltransferase per se, we performed in vitro lactylation experiments using purified recombinant protein of AARS1 (full length or amino acid residues 1–455, corresponding to the catalytic domain) (27) as an enzyme and purified proteins of histone H3 and H4, the most widely studied lactylated proteins, as substrates. The results showed that AARS1 was able to directly lactylate histone H3 and H4 in a manner dependent on both lactate and ATP (Figure 1C and Supplemental Figure 1A; supplemental material available online with this article; https://doi.org/10.1172/JCI174587DS1). The lactylated lysine residues of H3 and H4 in the in vitro lactylation assay were identified via mass spectrometry (Supplemental Figure 1B). Moreover, we used a synthetic H3K18 peptide (APRK^18^QLAT) as a substrate in the in vitro lactylation assay. Subsequent mass spectrometry also confirmed that AARS1 was indeed able to directly lactylate the H3 peptide at K18 in the presence of lactate and ATP (Figure 1D). Furthermore, 3D structural analysis indicated that mutation of amino acid residues (R77A, M100A, W176E, V218D, D239A) lining the catalytic pocket of AARS1 would disrupt its interaction with lactate (Supplemental Figure 1C). Accordingly, we found that 5M mutation of AARS1 totally abolished its lactyltransferase activity in vitro (Figure 1, C and D), again confirming the necessity of lactate binding for AARS1-mediated protein lactylation and that AARS1 directly uses lactate as a lactyl-donor. In addition, GST pull-down assay also revealed a direct interaction of AARS1 with the substrate histone H3 or H4 (Supplemental Figure 1D).

Since aminoacyl-tRNA synthetases catalyze a 2-step tRNA aminoacylation reaction, releasing pyrophosphate (PP_i_) and forming a reactive acyl adenylate (acyl-AMP) intermediate in the first step of the reaction (28), we speculated that AASR1-mediated protein lactylation would also produce PP_i_. As expected, the amount of PP_i_ released in the in vitro lactylation assay was positively correlated with lactate concentrations used, confirming PP_i_ as a product of the lactylation reaction (Figure 1E). In addition, it was reported that PP_i_ and acyl sulfonyladenosine (acyl-AMS) that mimic the tightly bound acyl-AMP intermediate could inhibit the catalytic activity of aminoacyl-tRNA synthetases (29). Indeed, we found that inclusion of PP_i_, AMP, and the synthetic lactyl-AMS (Supplemental Figure 1E) in our in vitro lactylation system significantly inhibited AARS1-mediated histone lactylation in a dose-dependent manner (Figure 1, C and D, and Supplemental Figure 1F). Moreover, as the binding affinity of l-alanine (*K_D_* = 0.45 μM) to AARS1 is slightly higher than but comparable to that of lactate (*K_D_* = 2.06 μM), inclusion of l-alanine in the reaction system was found to dose-dependently inhibit AARS1-mediated histone H3 lactylation (Supplemental Figure 1F). These data unambiguously demonstrated that AARS1 can directly bind to lactate and transfer it to lysine in a manner similar to its catalyzing of alanyl-tRNA formation, i.e., step 1: generate reactive lactyl-AMP and PP_i_ from lactate and ATP; step 2: transfer lactyl-group from lactyl-AMP to lysine residues of substrates (Figure 1F).

To compare the AARS1-mediated lactylation with previously reported lactyl-CoA–related lactylation, we employed lactyl-CoA instead of lactate in the in vitro lactylation assay to investigate the potential utilization of lactyl-CoA as a lactyl donor by AARS1. The results showed that AARS1 efficiently catalyzed histone H3 lactylation in the presence of physiological concentrations of ATP and lactate, but it failed to do so in the presence of even 100-fold higher concentrations of physiological lactyl-CoA (15) (Supplemental Figure 1G). Intriguingly, we found that high concentration of lactyl-CoA was able to trigger spontaneous protein lactylation in a nonenzymatic manner, a scenario that is not likely to exist in vivo because of the extremely low level of cellular lactyl-CoA (Supplemental Figure 1H). We next examined whether AARS1 moonlights as a lactyltransferase in vivo and found that knockdown of AARS1 significantly decreased histone H3 K18 lactylation levels in human GC cell line HGC27 (Supplemental Figure 1I). Notably, depletion of AARS1 in HGC27 cells substantially and globally decreased protein lactylation levels (Figure 1G), while knockdown of p300 only had a marginal effect (Supplemental Figure 1J). These data suggest that AARS1 catalyzes protein lactylation directly using ATP as energy source and lactate as lactyl-donor.

### AARS1 translocates into the nucleus in response to increased intracellular lactate.

We then asked whether and how AARS1, commonly understood as an enzyme residing in the cytoplasm and catalyzing ligation of l-alanine to tRNA, responds to lactate levels in cells. Interestingly, both fractionation and immunofluorescence assays showed that lactate treatment promoted AARS1 shuttling into the nucleus (Figure 1, H and I). Subsequent examination of amino acid sequences of AARS1 from various species revealed an evolutionarily conserved nuclear localization signal (NLS) motif in its C-terminal region (Figure 1J). We then generated an AARS1 mutant with the NLS deleted (ΔNLS) and examined its subcellular localization. As shown in Figure 1K, wild-type (WT) AARS1 was found to be localized in both cytoplasm and nucleus, whereas the ΔNLS mutant was found to be localized only in the cytoplasm. More importantly, addition of lactate significantly promoted the nuclear localization of WT AARS1 but not the ΔNLS mutant (Figure 1K).

Further, we investigated the mechanism through which lactate promotes nuclear translocation of AARS1. Proteins with NLS are usually transported into the nucleus via their interactions with importin-α. In this regard, we performed lactylation proteomics in HGC27 cells, which revealed that several importin-α subunits (KPNA1, 3, 4, and 6) were lactylated (Supplemental Figure 1K). Therefore, we speculated that AARS1 may interact with and directly lactylate these importin(s) in response to accumulation of intracellular lactate. One may expect that intracellular lactate can increase the interaction of AARS1 with importin, thus promoting its nuclear translocation. To test this hypothesis, we examined the interaction of AARS1 with the candidate importins, which revealed KPNA4 as a binding partner of AARS1 (Supplemental Figure 1L). Moreover, lactate promoted the interaction of AARS1 with KPNA4, while deletion of the NLS in AARS1 abolished such interaction (Supplemental Figure 1M), results suggesting that KPNA4 binds to the NLS motif of AARS1 to mediate its nuclear translocation.

### YAP and TEAD are directly lactylated by AARS1 and delactylated by SIRT1.

**Figure 2:**
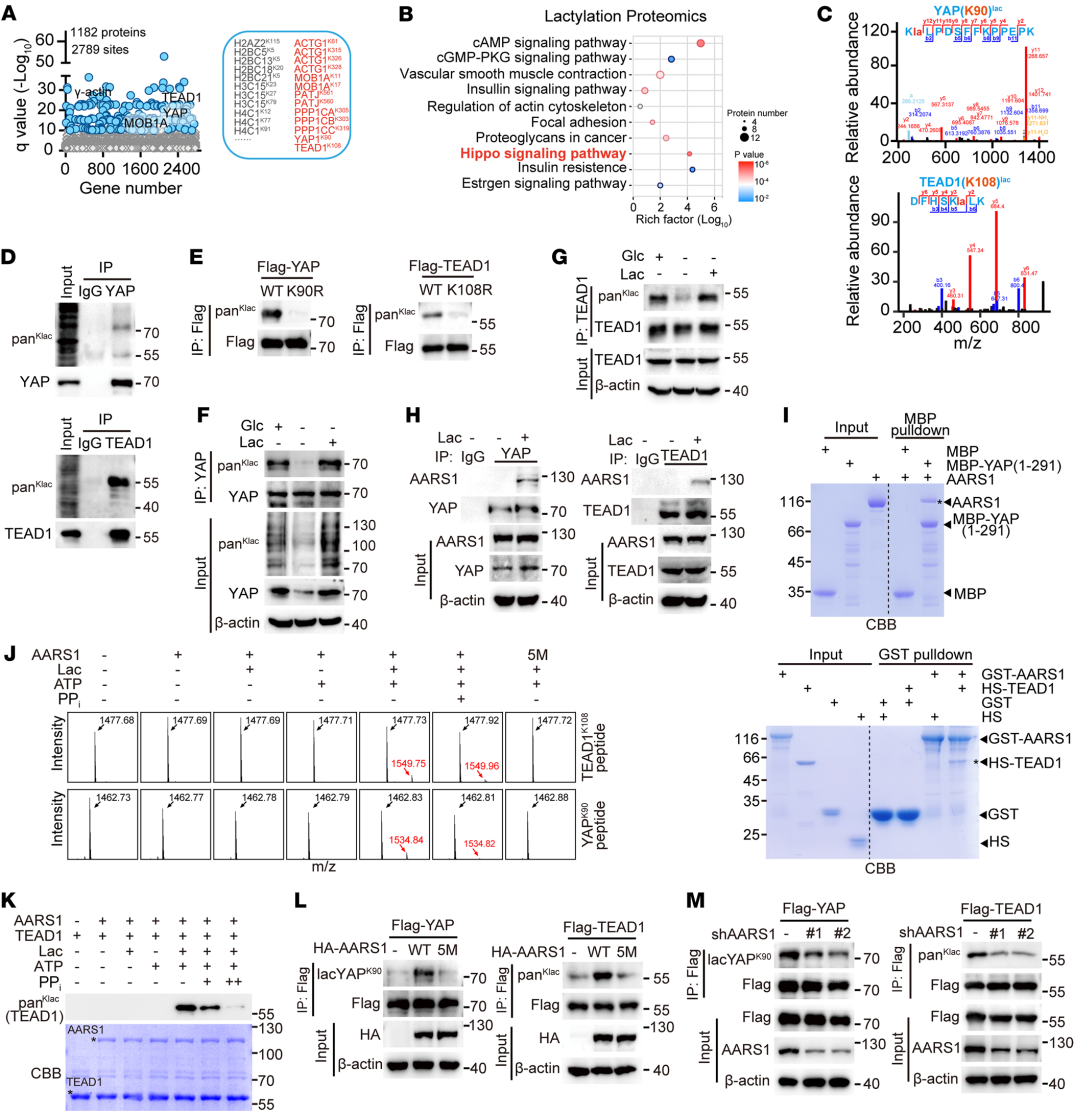
AARS1 directly interacts with and lactylates YAP-TEAD. (**A**) Lysine lactylome in lactate-treated HGC27 cells. A total of 1,182 lactylated proteins and 2,789 lactylated sites with *q* value (–log_10_) greater than 10 were identified. Histone sites modified through lactylation are shown in gray. Lactylation on Hippo pathway–associated components are shown in red. (**B**) KEGG pathway analysis of lactylated proteins identified using lactylation proteomics in HGC27 cells cultured in glucose-free medium supplemented with 25 mM lactate. (**C**) Mass spectra of lactylated sites of YAP (K90) and TEAD1 (K108). (**D**) Immunoblotting showing the lactylation of endogenous YAP and TEAD1 proteins using pan-Klac antibody. (**E**) Lactylation levels of exogenous YAP and TEAD1 in cells transfected with the indicated plasmids. (**F** and **G**) Lactylation levels of endogenous YAP (**F**) and TEAD1 (**G**) in lactate-treated HGC27 cells. Glc, glucose; Lac, lactate. (**H**) Coimmunoprecipitation analysis of the endogenous interaction between YAP (left) or TEAD1 (right) and AARS1 in lactate-treated HGC27 cells. (**I**) Pull-down assay showing the direct interaction between AARS1 and YAP-TEAD1. Top: MBP pull-down assay to detect interaction between AARS1 and MBP-YAP (1–291). Bottom: GST pull-down assay to detect interaction between GST-AARS1 and His-sumo-TEAD1 (HS-TEAD1). Asterisks represent the indicated proteins. (**J**) Mass spectrometry to determine the lactylation of the synthetic YAP K90 and TEAD1 K108 peptides catalyzed by AARS1 and its catalytic-dead mutant 5M in vitro. (**K**) Immunoblotting with pan-Klac antibody to detect AARS1-induced lactylation of TEAD1 in vitro. CBB staining showing the purified AARS1 and TEAD1 used in in vitro lactylation assay. Asterisks represent the AARS1 and TEAD1 proteins. The final concentrations of PP_i_ in the reaction mixture were 2 mM (+) and 10 mM (++), respectively. (**L**) Lactylation levels of YAP (left) and TEAD1 (right) in cells overexpressing AARS1 or its 5M mutant. (**M**) Lactylation levels of YAP (left) and TEAD1 (right) in AARS1-knockdown HEK293FT cells.

Notably, our lactylation proteomics study in HGC27 cells identified 2,789 unique Klac sites (lactylated lysines) in 1,182 proteins (Figure 2A). Among these proteins were multiple types of histones, which were previously reported to be lactylated (7). Sequence motif analysis showed that the Klac sites preferably locate downstream of serine or arginine residues (Supplemental Figure 2A). A subsequent Kyoto Encyclopedia of Genes and Genomes (KEGG) analysis indicated multiple cellular pathways, including cAMP, insulin, and Hippo, to be most likely regulated by protein lactylation (Figure 2B). To explore the possible role of protein lactylation in driving tumor cell proliferation, we then focused on the Hippo signaling pathway, in which several components or regulatory proteins, such as ACTG1, MOB1A, PPP1CA, YAP, and TEAD1, were found to be lactylated (Figure 2A). Notably, YAP and TEAD1 were lactylated at K90 and K108, respectively (Figure 2C), and both lactylated sites were found to be highly conserved (Supplemental Figure 2B).

We then performed immunoprecipitation assay to validate the mass spectra result. Indeed, immunoblotting using the anti-pan-Klac antibody repeatedly detected strong signals for lactylation of YAP-TEAD (Supplemental Figure 2C). Moreover, lactylation of endogenous YAP and TEAD1 were also observed in HGC27 cells (Figure 2D). Further, to confirm that K90 and K108 are the primary sites of YAP and TEAD1 lactylation, respectively, we mutated each residue to arginine (R) and examined their lactylation status. Indeed, the YAP K90R and TEAD1 K108R mutants showed almost no lactylation (Figure 2E). We further examined whether the lactylation of YAP and TEAD1 can respond to lactate levels. To this end, we cultured cells in media with different intracellular lactate concentrations. We found that glucose deprivation decreased the lactylation levels of YAP-TEAD1, while lactate treatment obviously rescued their lactylation (Supplemental Figure 2, D and E). Similar results were also obtained for endogenous YAP (Figure 2F) and TEAD1 (Figure 2G) in HGC27 cells.

To facilitate further study of YAP K90 lactylation, we generated a rabbit polyclonal antibody recognizing YAP K90 lactylation (hereafter referred to as lacYAP^K90^) using RLRK^lac^PDSFFKPPC peptide as an antigen. We first applied dot blot assay to test whether this lacYAP^K90^ antibody can recognize lactylated YAP using synthesized peptides corresponding to amino acid residues 87–99 of YAP, and found it can specifically detect the lactylated but not the unmodified peptides (Supplemental Figure 2F). Using this antibody, we then confirmed that lactate treatment increased the lactylation levels of endogenous YAP protein in HGC27 cells (Supplemental Figure 2G) and that YAP lactylation was completely abolished by K90R mutation (Supplemental Figure 2H) and YAP knockout (Supplemental Figure 2I). Moreover, pretreatment of this homemade antibody with a YAP K90lac peptide totally blocked its signal, i.e., abrogated its ability to recognize lactylated YAP in cells (Supplemental Figure 2J).

To investigate whether AARS1 is directly responsible for the lactylation of Hippo pathway components, we first confirmed by coimmunoprecipitation the interaction of AARS1 with YAP-TEAD1 (Figure 2H and Supplemental Figure 2, K and L). Moreover, cellular fractionation assay clearly demonstrated that such interaction mainly occurred in the nucleus (Supplemental Figure 2M). Further in vitro pull-down assays using purified recombinant proteins of AARS1 and YAP-TEAD1 revealed the interaction as a direct one (Figure 2I). We next performed in vitro lactylation experiments using synthetic peptides of TEAD1 K108 (RDFHSK^108^LKDQTC) and YAP K90 (PMRLRK^90^LPDSFC) as substrates. Mass spectrometry analysis showed that purified AARS1 protein was indeed able to directly lactylate the synthetic TEAD1 K108 and YAP K90 peptides in the presence of lactate and ATP (Figure 2J). Similarly, our in vitro lactylation assay using purified recombinant protein of TEAD1 as a substrate also showed that AARS1 was able to directly lactylate WT TEAD1 (Figure 2K), but not its K108R mutant (Supplemental Figure 2N). Also, we found that AARS1 5M mutant failed to lactylate either YAP or TEAD1 (Figure 2J), and that inclusion of PP_i_ or l-alanine significantly inhibited the AARS1-mediated lactylation of YAP-TEAD1 (Figure 2, J and K, and Supplemental Figure 2, O and P). Consistently, overexpression of WT AARS1 but not its 5M mutant in HEK293FT cells promoted lactylation of YAP-TEAD (Figure 2L), while knockdown of AARS1 markedly decreased the lactylation levels of YAP-TEAD (Figure 2M).

To probe possible enzymes responsible for the delactylation of YAP, we treated HEK293FT cells with the histone deacetylase inhibitor trichostatin A or the sirtuin inhibitor nicotinamide (NAM). The results showed that NAM treatment significantly increased the lactylation of YAP (Supplemental Figure 2Q). To further identify the specific enzyme responsible for YAP delactylation, we performed a mini-screening in YAP-overexpressing cells cotransfected with individual members of the sirtuin family of deacetylases (SIRT1–SIRT7). The results showed that overexpression of SIRT1, but not of other members of this family, substantially reduced the lactylation levels of YAP (Supplemental Figure 2R). Meanwhile, our coimmunoprecipitation assay showed an interaction of SIRT1 with YAP (Supplemental Figure 2S). Moreover, unlike WT SIRT1, a catalytically deficient mutant of SIRT1 (H363Y) failed to reduce the lactylation levels of YAP in cells (Supplemental Figure 2T). This was further confirmed by the results of an in vitro delactylation assay showing that purified SIRT1, but not the H363Y mutant, eliminated lactylation of synthetic peptides of both YAP K90lac and TEAD1 K108lac (Supplemental Figure 2U).

### Lactylation of YAP-TEAD promotes expression of Hippo pathway target genes.

**Figure 3:**
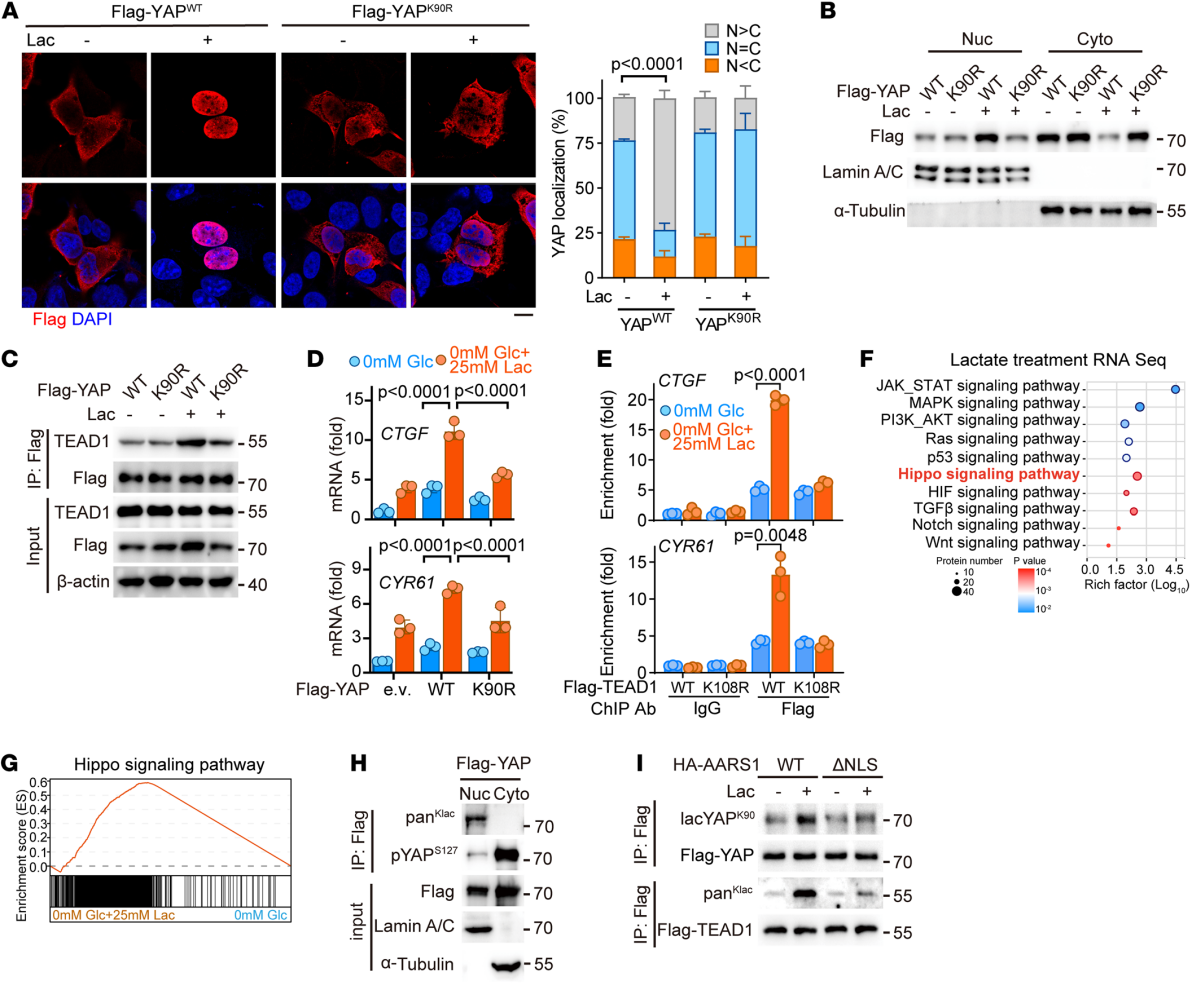
Lactylation promotes nuclear localization and stabilization of YAP-TEAD. (**A**) Left: Immunofluorescence analysis using anti-FLAG antibody showing nuclear translocation of YAP in HEK293A cells transfected with FLAG-tagged YAP or its K90R mutant following lactate treatment. Right: The signal intensity of FLAG-YAP was quantified using ImageJ software (NIH) (*n* = 3). N, nuclear localization; C, cytosolic localization; Lac, lactate. Data are presented as mean ± SD. Scale bar: 5 μm. (**B**) Nucleocytoplasmic distribution of heterologously expressed YAP or its K90R mutant in lactate-treated cells. Nuc, nuclear localization; Cyto, cytosolic localization. (**C**) Coimmunoprecipitation analysis showing the interaction of YAP or its K90R mutant with TEAD1 in lactate-treated cells. (**D**) Real-time quantitative PCR (qPCR) showing the mRNA levels of *CTGF* and *CYR61* in HEK293A cells overexpressing YAP or its K90R mutant following lactate treatment (*n* = 3). Data are presented as mean ± SD. Glc, glucose. (**E**) ChIP-qPCR analysis for the enrichment of TEAD1 or its K108R mutant on the indicated genes’ promoter in lactate-treated HEK293FT cells (*n* = 3). Data are presented as mean ± SD. (**F**) KEGG analysis of the differentially expressed genes in the glucose-deprived HGC27 cells with or without 25 mM lactate. (**G**) Gene set enrichment analysis of the Hippo pathway signature in the glucose-deprived HGC27 cells with or without 25 mM lactate. (**H**) Nucleocytoplasmic distribution of lactylation and phosphorylation of YAP in YAP-overexpressing HEK293FT cells. (**I**) Lactylation of exogenous YAP and TEAD1 in lactate-treated HEK293A cells transfected with AARS1 or its NLS-deletion (ΔNLS) mutant. Unpaired 2-tailed Student’s *t* test (**A**, **D**, and **E**).

To assess the functional consequence of YAP K90 lactylation, we transfected HEK293A cells with WT YAP, or with its K90R mutant designed to mimic a lactylation-deficient/resistant state, and examined their subcellular localization frequency in glucose-free medium supplemented or not supplemented with lactate. Both immunofluorescence (Figure 3A) and cellular fractionation (Figure 3B) assays revealed that lactate strongly promoted nuclear localization of WT YAP but not its K90R mutant, suggesting that K90 lactylation is required for lactate-induced YAP activation. Consistently, lactate enhanced the interaction of TEAD1 with WT YAP but not its K90R mutant (Figure 3C). Moreover, lactate significantly increased the mRNA levels of *CTGF* and *CYR61* in cells overexpressing WT YAP, but a lesser effect was observed in cells overexpressing the YAP K90R mutant (Figure 3D). Also, a luciferase reporter assay showed that lactate promoted WT YAP–induced but not K90R mutant–induced transactivation of TEADs (Supplemental Figure 3A). Similarly, we also explored the effect of lactylation on TEAD1 by generating a K108R mutant to mimic a lactylation-deficient/resistant state. Chromatin immunoprecipitation (ChIP) assay showed that lactate treatment enhanced the occupancy of WT TEAD1 but not its K108R mutant on the promoters of CTGF and CYR61 (Figure 3E). Overall, these data demonstrated that intracellular lactate promotes lactylation levels and transcriptional activity of YAP-TEAD1. Supporting this, RNA sequencing (RNA-Seq) analysis indicated that the Hippo pathway can indeed respond to treatment with lactate (Figure 3, F and G).

Since the lactylation sites of YAP (K90) and TEAD1 (K108) identified in this work were previously found to be ubiquitinated (30, 31), we went on to investigate the interplay between lactylation and ubiquitination of YAP-TEAD1. Given that nuclear localization of YAP is essential for transactivation of TEADs, we first examined the lactylation and ubiquitination levels of YAP and TEAD1 in the nucleus and cytoplasm. Our nucleocytoplasmic fractionation assay showed most of the lactylated YAP and TEAD1 to be localized in the nucleus, while S127-phosphorylated YAP or ubiquitinated YAP and TEAD1 were mainly distributed in the cytoplasm (Figure 3H and Supplemental Figure 3B). Then we examined a possible effect of lactate on YAP-TEAD1 ubiquitination and found that lactate treatment decreased ubiquitination levels of YAP-TEAD1 in a dose-dependent manner (Supplemental Figure 3C), consistent with our findings that increased levels of lactate promote nuclear localization of AARS1 and its interaction with YAP-TEAD (Figure 1K and Figure 2H). Moreover, WT AARS1 promoted the lactylation of YAP-TEAD1, but the ΔNLS mutant failed to do so (Figure 3I); and depletion of AARS1 significantly promoted ubiquitination of YAP-TEAD1 (Supplemental Figure 3D). Meanwhile, lactate treatment also inhibited the interaction of YAP with XPO1 (Supplemental Figure 3E), a protein previously shown to bind with and facilitate nuclear export of YAP (32), results suggesting that lactylation of YAP impaired its shuttling into the cytoplasm for ubiquitination.

### AARS1 is a direct target gene of YAP-TEAD.

**Figure 4:**
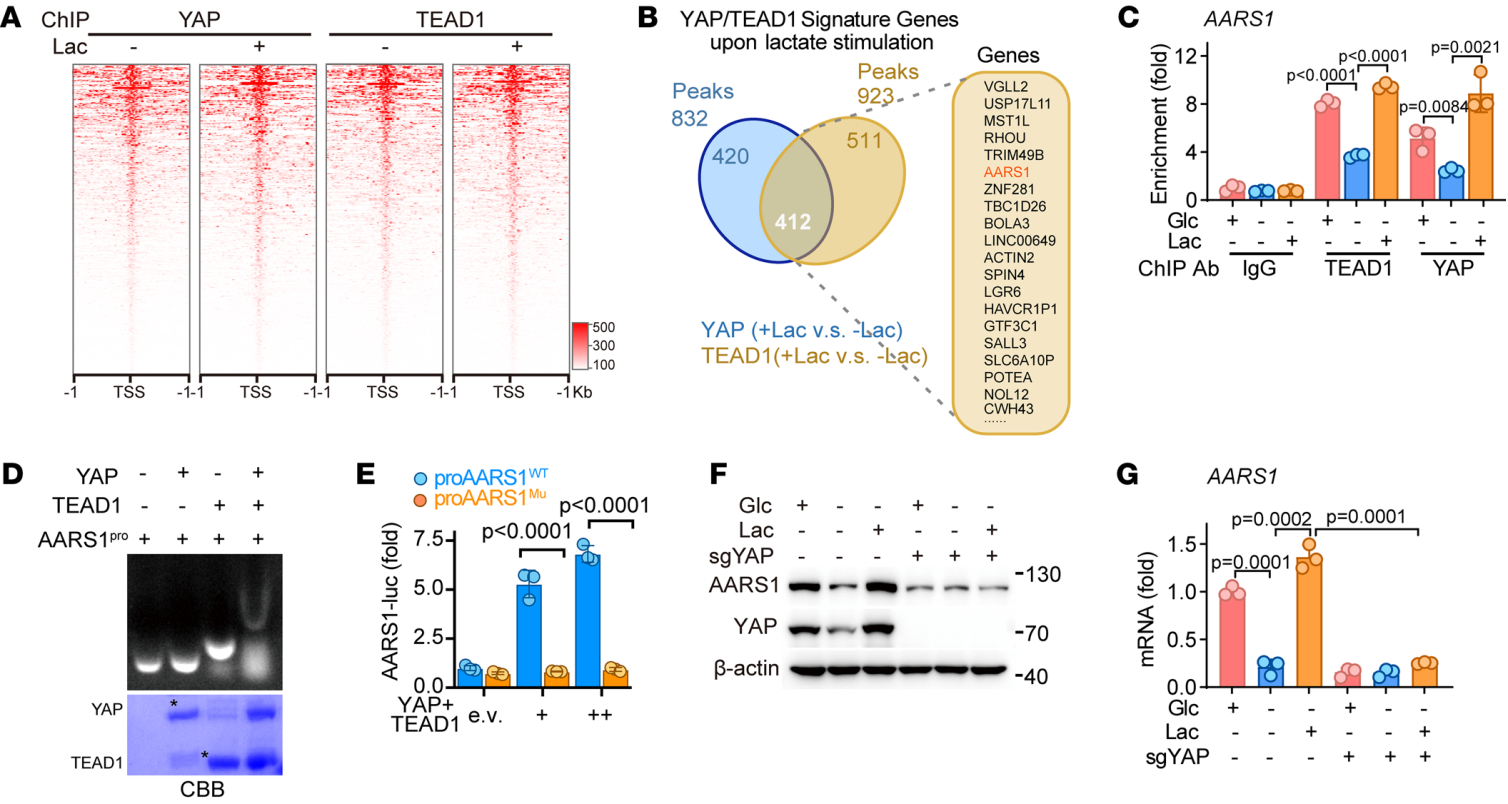
AARS1 and YAP-TEAD form a positive-feedback loop. (**A**) ChIP-Seq analysis heatmap representing the distribution of YAP or TEAD1 binding relative to the gene transcription start site (TSS) in cells cultured in glucose-free medium treated with or without lactate. Lac, lactate. (**B**) Venn diagram illustrating the overlap of YAP- and TEAD1-enriched genes upon lactate stimulation. The top 20 genes are shown. (**C**) ChIP-qPCR showing the enrichment of YAP and TEAD1 on the AARS1 promoter in lactate-treated HGC27 cells (*n* = 3). Glc, glucose. Data are presented as mean ± SD. (**D**) Top: Gel shift analysis showing the binding of YAP and TEAD1 to the synthetic DNA probe containing TEAD1-binding site on the AARS1 promoter. Bottom: CBB staining of purified recombinant YAP (MBP-YAP 1-291) and TEAD1 proteins used in gel shift assay. (**E**) Luciferase activity of WT or mutant (Mu) AARS1 promoter vectors in YAP- and TEAD1-overexpressing HEK293FT cells (*n* = 3). Data are presented as mean ± SD. (**F**) Immunoblotting of AARS1 protein levels in YAP-knockout AGS cells upon lactate treatment. (**G**) *AARS1* mRNA levels in YAP-knockout cells upon lactate stimulation (*n* = 3). Data are presented as mean ± SD. Unpaired 2-tailed Student’s *t* test (**C**, **E**, and **G**).

To characterize the genome-wide signature genes of YAP-TEAD1 upon lactate stimulation, we performed a ChIP-Seq analysis. Clearly, lactate treatment enhanced the enrichment of YAP-TEAD1 around the transcription start site region (Figure 4A). The ChIP-Seq analysis identified 832 and 923 peaks for YAP and TEAD1, respectively, with 412 overlapping peaks. Notably, YAP-TEAD1 was enriched on the promoter of AARS1 upon lactate treatment, indicating AARS1 as a downstream target gene of YAP-TEAD1 (Figure 4B). Indeed, a conserved TEAD1-binding motif was found on the promoter of AARS1 (Supplemental Figure 4A). Moreover, a ChIP assay revealed binding of YAP-TEAD1 to the promoter of AARS1 (Figure 4C). These observations were further confirmed by a gel shift assay showing that TEAD1 alone, but not YAP, retarded the DNA probe corresponding to the AARS1 promoter and that YAP-TEAD1 caused a supershift of the probe (Figure 4D).

To further test whether YAP-TEAD1 regulates the transcription of AARS1 by binding to the predicted TEAD1-binding motif on the AARS1 promoter, we constructed luciferase reporter gene vectors containing the WT (proAARS1^WT^) or mutated (proAARS1^Mu^) TEAD1-binding site (Supplemental Figure 4B). As expected, overexpression of YAP-TEAD1 in HEK293FT cells increased the luciferase reporter gene activity of the WT vector in a dose-dependent manner but did not affect the activity of the mutant vector (Figure 4E and Supplemental Figure 4C). In addition, both protein and mRNA levels of AARS1 were significantly increased in HGC27 cells upon treatment with lactate, whereas knockout of YAP abolished such effects (Figure 4, F and G). Together, these results indicated that AARS1 is a direct target gene of YAP-TEAD1, and that intracellular lactate drives a positive-feedback loop between AARS1 and YAP-TEAD1 (Supplemental Figure 4D).

### AARS1 is upregulated in human GC and associated with poor prognosis.

**Figure 5:**
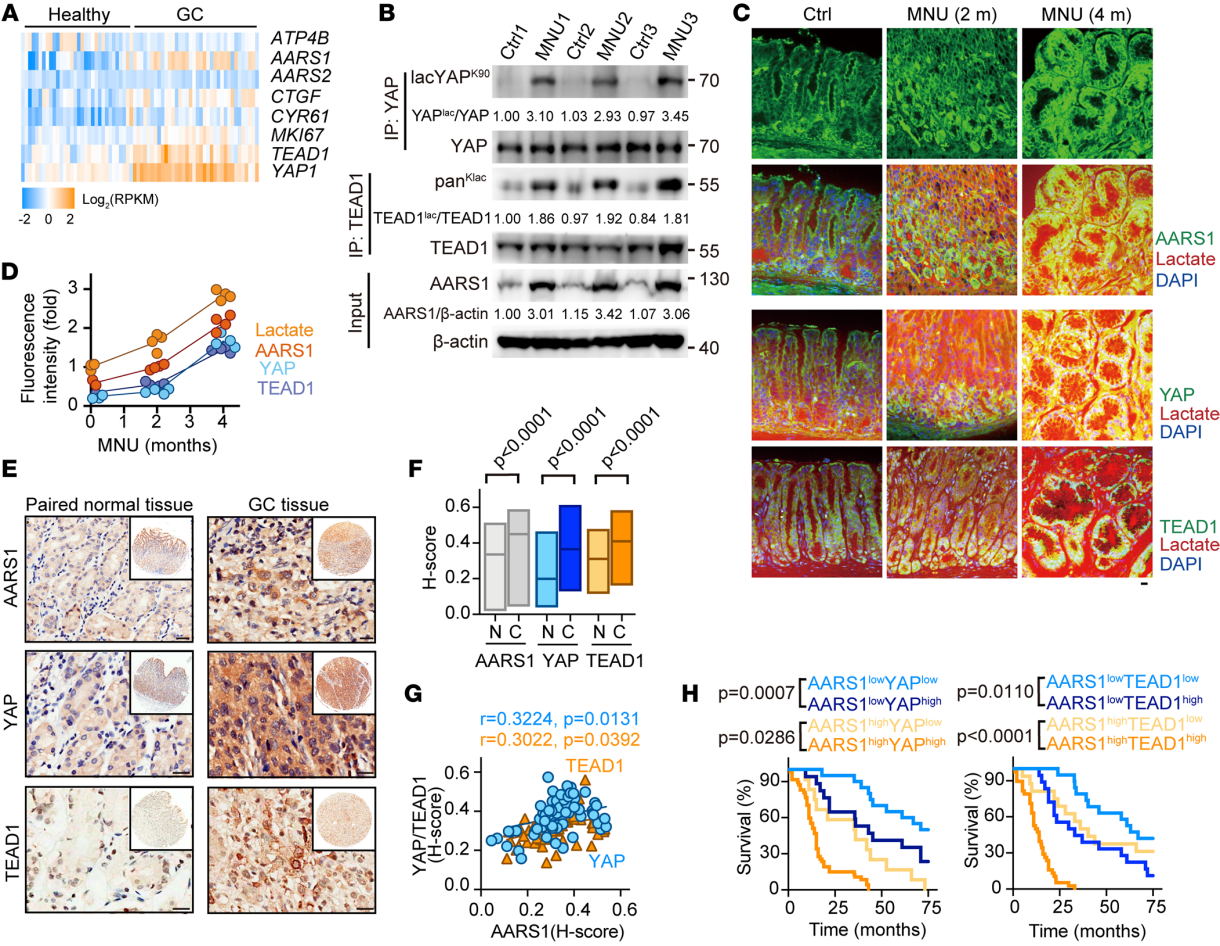
AARS1 is upregulated in gastric cancer and associated with bad clinical outcomes. (**A**) Heatmap showing the transcription of the indicated genes in healthy gastric tissues and GC tissues from the GEO database (GSE13911). (**B**) Immunoblotting of AARS1 levels and YAP-TEAD1 lactylation levels in mouse normal (Ctrl) and MNU-induced GC tissues. Relative YAP-TEAD1 lactylation and AARS1 levels calculated by gray value analysis are shown. (**C**) Immunofluorescence images of lactate, AARS1, YAP, and TEAD1 in the gastric tissues of the MNU-induced GC model at the indicated times. Scale bar: 10 μm. (**D**) Fluorescence intensity of lactate, AARS1, YAP, and TEAD1 in the murine GC model from **E** (*n* = 4). Data are presented as mean ± SD. (**E**) Immunohistochemical staining of AARS1, YAP, and TEAD1 in GC tissues and paired healthy tissues. Scale bars: 50 μm. (**F**) Histoscore (H-score) of AARS1, YAP, and TEAD1 in GC tissues (C) and paired healthy tissues (N) by a semiquantitative assessment. (**G**) Correlation between the H-score for YAP or TEAD1 and that for AARS1 in GC tissues. (**H**) Kaplan-Meier survival curve of patients with GC with AARS1/YAP (left) or AARS1/TEAD1 (right) at high or low levels from tissue microarray. Unpaired 2-tailed Student’s *t* test (**F**); Spearman’s rank correlation (**G**); log-rank test (**H**).

To assess the clinical relevance of AARS1 in GC, we analyzed AARS1 transcription in the Gene Expression Omnibus (GEO) database. As expected, the expression of *ATP4B*, a known parietal cell marker in normal gastric epithelium, was lost, while *MKI67*, *YAP1*, *TEAD1*, *CTGF*, and *CYR61* were all significantly upregulated, in GC (Figure 5A). Notably, the transcription of *AARS1* but not *AARS2* was much higher in GC tissues than in healthy tissues (Figure 5A). Moreover, the mRNA levels of *AARS1* were positively correlated with those of *MKI67* and *YAP1* (Supplemental Figure 5A). We then collected 6 human GC samples paired with adjacent normal tissues and confirmed the elevated expression levels of AARS1 in GC (Supplemental Figure 5B). Furthermore, we monitored the expression of AARS1, YAP, and TEAD1 during *N*-methyl-*N*-nitrosourea–induced (MNU-induced) mouse GC progression and found that expression levels of AARS1 and lactylation levels of YAP-TEAD1 were obviously increased upon MNU treatment (Figure 5B). In addition to the expression of AARS1 and YAP-TEAD1, the levels of lactate were also progressively increased along with the MNU-induced GC progression (Figure 5, C and D). Moreover, not only the expression of YAP and AARS1 but also their nuclear localization were enhanced in MNU-induced tumors compared with normal tissues (Supplemental Figure 5, C and D).

Subsequently, we examined the expression of AARS1 by immunohistochemical staining on a human GC tissue array containing 90 GC specimens paired with normal ones. Consistent with the above results, the expressions of AARS1, YAP, and TEAD1 were found to be significantly upregulated in GC tissues compared with associated normal tissues (Figure 5, E and F). The expression levels of AARS1 were found to be correlated with those of YAP-TEAD1 in GC tissues (Figure 5G and Supplemental Tables 1 and 2). Further Kaplan-Meier survival analysis showed that high expression levels of AARS1, especially in combination with high expression of YAP-TEAD1, strongly predicted a poor prognosis for patients with GC in this cohort (Figure 5H). In addition, expression levels of AARS1 were found to be positively correlated with *Helicobacter pylori* infection, tumor size, and tumor stages (weakly with lymph node metastasis) (Table 1).

### AARS1 promotes GC growth in a manner dependent on YAP-TEAD lactylation.

**Figure 6:**
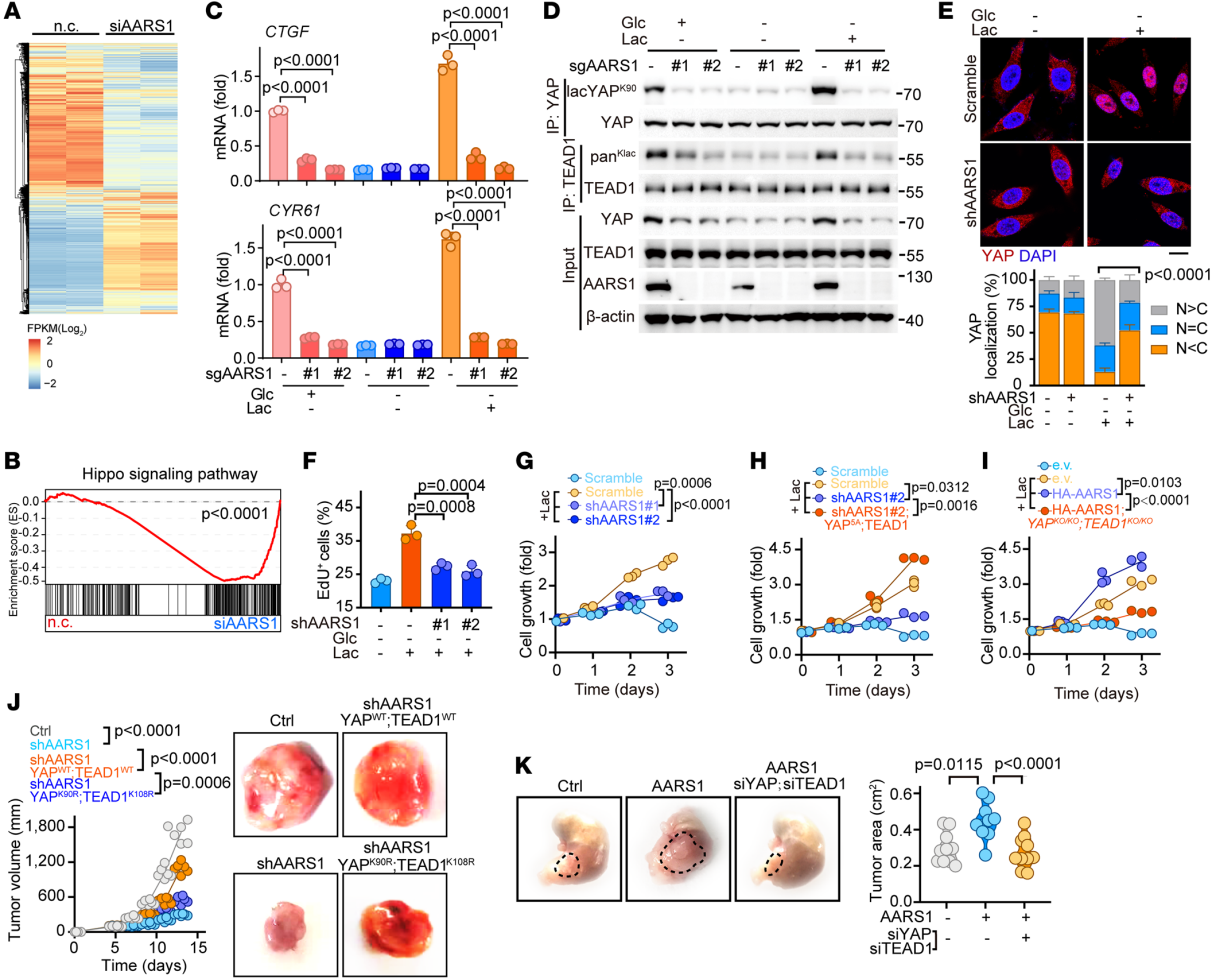
AARS1 promotes gastric cancer overgrowth via YAP-TEAD lactylation. (**A**) Heatmap of differentially expressed genes in AARS1-knockdown cells upon lactate treatment. RNA-Seq was performed to evaluate the transcriptomics of AARS1 siRNA–transfected HGC27 cells cultured in glucose-free medium with lactate for 12 hours. (**B**) GSEA of Hippo signature genes in lactate-treated HGC27 cells. Normalized enrichment score and FDR are shown. (**C**) mRNA levels of *CTGF* and *CYR61* in AARS1-knockout cells upon lactate treatment (*n* = 3). Glc, glucose; Lac, lactate. Data are presented as mean ± SD. (**D**) Lactylation levels of YAP and TEAD1 in AARS1-knockout cells upon lactate treatment. (**E**) Top: Immunofluorescence images of YAP localization in scramble control and AARS1-knockdown cells upon lactate treatment for 12 hours. Bottom: Quantification of YAP signal intensity (*n* = 3). N, nuclear localization; C, cytosolic localization. Data are presented as mean ± SD. Scale bar: 5 μm. (**F**) Percentage of EdU^+^ cells in AARS1-knockdown cells treated with lactate for 12 hours (*n* = 3). Data are presented as mean ± SD. (**G**) Cell growth curves of scramble control and AARS1-knockdown cells upon lactate treatment (*n* = 3). Data are presented as mean ± SD. (**H**) Rescue assay showing cell growth of AARS1-knockdown cells after enforced expression of YAP^5A^ and TEAD1 upon lactate treatment (*n* = 3). YAP^5A^: S61A, S109A, S127A, S164A, S397A. Data are presented as mean ± SD. (**I**) Cell growth curves of HA-AARS1–overexpressing cells after YAP and TEAD1 depletion upon lactate treatment (*n* = 3). Data are presented as mean ± SD. (**J**) Xenograft murine GC model after subcutaneous injection with HGC27 cells transfected with indicated plasmids. Tumor growth curves (left) and representative tumor images (right) are shown (*n* = 6). (**K**) Orthotopic murine GC model after injection with HGC27 cells transfected with indicated plasmids. Left: Representative images. Right: Measurement of tumor area (*n* = 10). Unpaired 2-tailed Student’s *t* test (**C**, **E**, **F**, and **K**); 1-way ANOVA (**G**–**J**).

To investigate whether AARS1 promotes tumor cell growth in a manner dependent on the Hippo pathway, we performed an RNA-Seq analysis of AARS1-knockdown HGC27 cells (Figure 6A and Supplemental Figure 6A). Gene set enrichment analysis (GSEA) showed a negative enrichment of the Hippo pathway signature genes upon AARS1 knockdown (Figure 6B). This result was further validated by a quantitative PCR assay showing that AARS1 knockout dramatically reduced the mRNA expressions of *CTGF* and *CYR61* in the presence of sufficient lactate (normal medium or glucose-free medium with exogenous lactate), but had no effect on deficiency of lactate (glucose-free medium without exogenous lactate) (Figure 6C). Consistent with these observations, depletion of AARS1 significantly decreased the lactylation levels of endogenous YAP-TEAD1 even in the presence of sufficient lactate (Figure 6D). Also, knockdown of AARS1 abrogated the promoting effect of lactate on the retention of YAP in the nucleus (Figure 6E). These results further confirmed that AARS1 is required for the lactylation of YAP-TEAD1 and therefore lactate-induced expression of Hippo pathway target genes.

Next, we assessed the potential regulatory effect of AARS1 on YAP-driven tumor growth. Knockdown of AARS1 in HGC27 cells markedly decreased lactate-induced EdU^+^ cell populations (Figure 6F and Supplemental Figure 6B) and suppressed cell growth (Figure 6G) and colony formation efficiency (Supplemental Figure 6C). However, overexpression of TEAD1 together with a constitutively active (5A) mutant of YAP rescued the growth of the AARS1-knockdown HGC27 cells (Figure 6H and Supplemental Figure 6, D and E). Conversely, overexpression of AARS1 in WT HGC27 cells promoted their growth, while depletion of YAP-TEAD1 abolished such effects (Figure 6I and Supplemental Figure 6, F and G). To further investigate the pathological function of AARS1 in tumorigenesis, we generated subcutaneous and orthotopic mouse GC models and found that knockdown of AARS1 markedly inhibited tumor growth, while enforced expression of YAP-TEAD1 abolished this inhibitory effect (Figure 6J). However, overexpression of the YAP (K90R)–TEAD1 (K108R) mutants only slightly rescued the growth of tumors inhibited by AARS1 knockdown (Figure 6J). Consistently, mice orthotopically injected with HGC27 cells stably expressing AARS1 had larger tumors in their stomachs than the control group, whereas silencing the expression of YAP-TEAD1 abrogated AARS1 overexpression–induced tumor growth (Figure 6K).

To investigate whether the regulatory effect of AARS1 on cell proliferation depends on its canonical function as a tRNA synthetase or its moonlighting function as a lactyltransferase (i.e., YAP-TEAD1 lactylation), we reintroduced WT, ΔNLS, and 5M mutant of AARS1 back into AARS1-knockout AGS cells. Immunoblotting showed that AARS1 depletion significantly reduced YAP-TEAD1 lactylation levels in cells treated with lactate, while reintroduction of WT AARS1 but not ΔNLS and 5M mutant rescued YAP-TEAD1 lactylation in AARS1-knockout cells (Supplemental Figure 6H). Subsequently, we used *O*-propargyl-puromycin (OP-Puro), an analog of puromycin that can incorporate into nascent polypeptide chains within cells, to evaluate the impact of NLS deletion on the tRNA synthetase function of AARS1. The result of flow cytometry analysis showed no significant difference in protein synthesis between AARS1-knockout cells reconstituted with WT AARS1 and those reconstituted with ΔNLS mutant AARS1, suggesting that NLS deletion did not affect the canonical function of AARS1 (Supplemental Figure 6I). However, the result of EdU cell proliferation assay showed that only WT AARS1, but not the ΔNLS and 5M mutants, rescued the cell proliferation of AARS1-knockout cells (Supplemental Figure 6J).

Furthermore, we performed a xenograft GC model to evaluate in vivo the pathological function of AARS1. The results showed that ectopic expression of WT AARS1 effectively rescued the growth of tumors derived from AARS1-knockout cells, while the ΔNLS mutant failed to do so (Supplemental Figure 6K). In addition, we overexpressed WT YAP-TEAD1 and lactylation-deficient mutants of YAP (K90R)–TEAD1 (K108R) in AARS1-overexpressed AGS cells and examined their proliferation. The results of EdU assay showed that WT YAP-TEAD1, but not lactylation-deficient mutants, significantly promoted cell proliferation in AARS1-overexpressing cells (Supplemental Figure 6L). Similar results were obtained in a xenograft GC model (Supplemental Figure 6M). Together, these findings indicate that the lactyltransferase function instead of the tRNA synthetase function of AARS1 plays an essential role in controlling GC growth.

### GC-associated R77Q mutation of AARS1 promotes its lactyltransferase activity.

**Figure 7:**
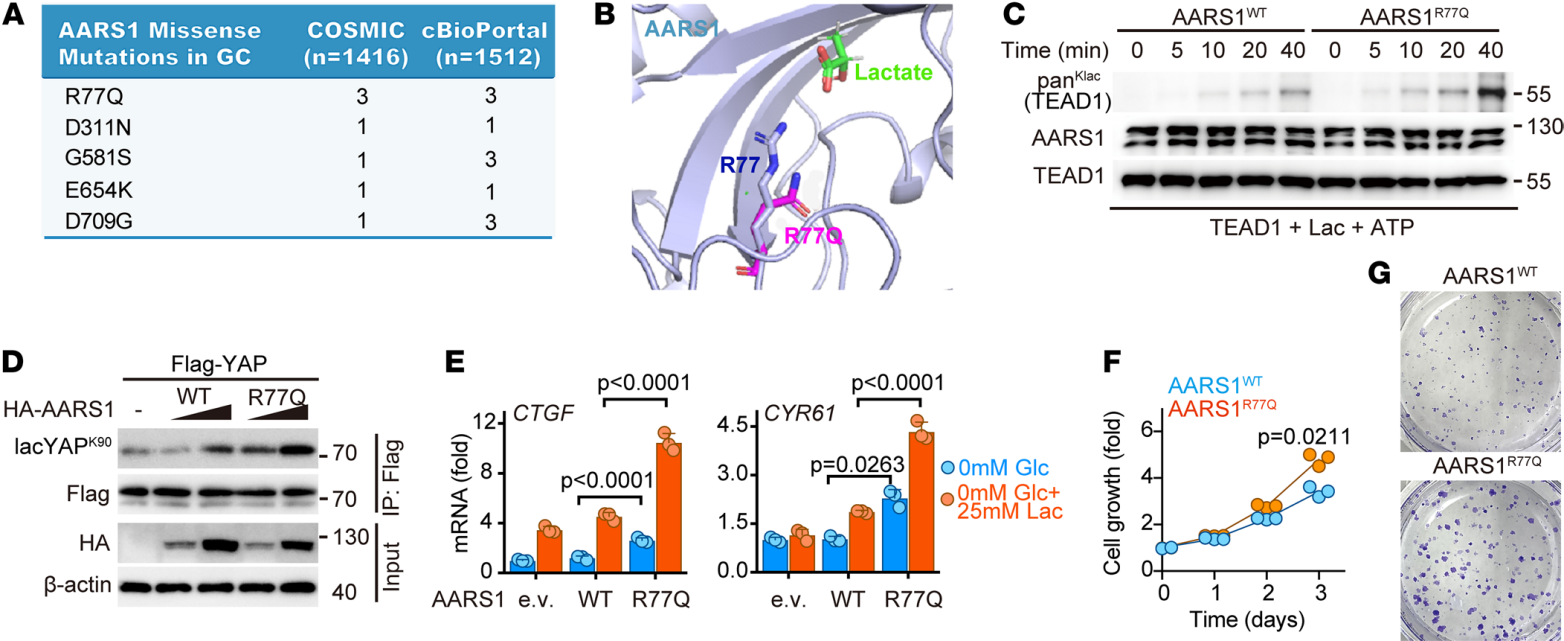
R77Q mutation promotes AARS1 activity and GC cell growth. (**A**) AARS1 mutations in patients with GC from COSMIC and cBioPortal databases. (**B**) Structural view of R77, R77Q, and lactate in the catalytic core of AARS1. (**C**) In vitro lactylation assay to assess the catalytic efficiencies of WT AARS1 and R77Q mutant AARS1. (**D**) Immunoblot analysis of YAP lactylation in HEK293FT cells cotransfected with FLAG-YAP and WT or R77Q mutant AARS1. (**E**) Real-time qPCR analysis of *CTGF* and *CYR61* mRNA levels in HEK293FT cells cotransfected with FLAG-YAP and WT AARS1 or R77Q mutant AARS1 treated with or without lactate (*n* = 3). Data are presented as mean ± SD. (**F** and **G**) Cell growth curves (**F**) and colony formation assay (**G**) of WT AARS1– and R77Q mutant–overexpressing AGS cells (*n* = 3). Data are presented as mean ± SD. Unpaired 2-tailed Student’s *t* test (**E**); 1-way ANOVA (**F**).

Given the clinical relevance (Figure 5) and the tumor-promoting role (Figure 6) of AARS1, we further analyzed AARS1 mutations in the COSMIC and cBioPortal databases and found that R77Q was the most common AARS1 mutation in patients with GC (Figure 7A). Since R77 is in the catalytic pocket of AARS1 (Figure 7B), we reasoned that R77Q mutation might affect the substrate binding or enzymatic activity of AARS1. To test this possibility, we first performed a coimmunoprecipitation assay and found no effect of the R77Q mutation on the interaction between AARS1 and YAP-TEAD1 (Supplemental Figure 7). Subsequently, however, our in vitro lactylation assay using TEAD1 as a substrate showed that the R77Q mutation seemingly increased the catalytic efficiency of AARS1 (Figure 7C). Consistently, cotransfection of 293FT cells with YAP and WT or R77Q-mutated AARS1 showed significantly greater lactylation of YAP when the R77Q mutant was used than when the WT AARS1 was used (Figure 7D). Moreover, cell growth and colony formation assay showed significantly greater GC cell growth when the R77Q mutant was overexpressed than when WT AARS1 was overexpressed (Figure 7, E and F). In keeping with these observations, the expressions of *CTGF* and *CYR61* were also notably upregulated in the R77Q mutant–overexpressing cells compared with those in the WT AARS1–overexpressing cells (Figure 7G).

## Discussion

A newly defined posttranslational modification, namely lactylation, has been suggested in recent studies to play important roles in epigenetic regulation of gene expression and to be associated with human diseases such as inflammation, Alzheimer’s disease, and cancer (7, 11, 33). In this study, we rediscovered the tRNA synthetase AARS1 to be a moonlighting but bona fide lactyltransferase that can directly use lactate as a donor of lactyl-group and ATP as an energy source — and on that basis, we revealed a noncanonical function of lactate in tumor cells, i.e., to transmit a YAP-TEAD1–activating cell proliferation–promoting signal via lactylation, explaining from a new angle how tumors benefit from the Warburg effect.

### AARS1 as a lactyltransferase and a sensor of intracellular lactate.

It has been proposed that p300 may function as a lactyltransferase to catalyze histone lactylation by using lactyl-CoA as a lactyl-donor (7). However, the enzymes that produce lactyl-CoA from lactate in mammalian cells remain unknown, and the levels of lactyl-CoA in tumor cells are extremely low (15). As a major finding of this current work, we unambiguously identified AARS1 as a lactyltransferase able to catalyze protein lactylation using free lactate and ATP, which are abundant in cells, especially in proliferating tumor cells. Notably, we provided extensive and direct evidence of AARS1’s lactyltransferase activity, in particular, by the in vitro lactylation assay using high-purity recombinant protein of AARS1 as an enzyme, and purified proteins of histones and TEAD1, or synthetic peptides, as substrates. Meanwhile, we found that l-alanine can inhibit the lactyltransferase activity of AARS1 by competing with lactate to bind the same site of the catalytic pocket in AARS1. Therefore, AARS1-mediated protein lactylation may serve as a rapid response mechanism to dynamic cellular lactate metabolism, which may directly intersect l-alanine abundance and protein synthesis. Interestingly, we accidentally found in vitro that high concentrations of lactyl-CoA may trigger spontaneous protein lactylation, which most likely is an artifact that could not widely occur in vivo owing to low levels of cellular lactyl-CoA (34). That said, we could not rule out the possibility that low concentrations of lactyl-CoA in cells, via nonenzymatic lactylation, may contribute cumulatively to degenerative processes such as aging.

Most recently, Sun et al. reported that AARS1 and AARS2 may potentially function as lactyltransferases for METTL16 (35), while Mao et al. demonstrated that AARS2 directly catalyzes lactylation of the mitochondrial proteins PDHA1 and CPT2 (36), implying a general mechanism of catalysis of protein lactylation by AARS1/2. AARS2 has been shown to specifically localize on mitochondria and to not have an NLS motif, while AARS1 was found to have an NLS motif but to normally localize throughout the cytoplasm. Importantly, we found that AARS1 can sense increased levels of intracellular lactate and shuttle into the nucleus, where it interacts with the YAP-TEAD1 complex and lactylates both YAP and TEAD1. Our previous findings suggested that amino acids can enhance the interactions between the corresponding aminoacyl-tRNA synthetases and their substrates to catalyze lysine aminoacylations (37). Similarly, here we found AARS1 interacting with YAP-TEAD1 both in cells and in vitro, and found that lactate meanwhile can enhance their interactions. Thus, AARS1 appears to be a sensor of intracellular lactate and a general lactyltransferase.

### Function fate of AARS1 as a lactyltransferase or a tRNA synthetase.

Our current work raises the important question of what are the cellular signals that control the substrate preference of AARS1, and what mechanism decides the function fate of AARS1 as a lactyltransferase or a tRNA synthetase. In this regard, note that the intracellular concentration of l-alanine is approximately 0.24 mM in Hela cells (38), and the physiological concentration of lactate ranges from 0.5 to 20 mM (39) and can reach up to 40 mM in tumor tissues (40). Therefore, on one hand, l-alanine can inhibit the lactyltransferase activity of AARS1 by directly competing with lactate to bind AARS1. On the other hand, increased intracellular lactate might also regulate the tRNA synthetase activity of AARS1 by (a) competing with l-alanine to decrease the alanyl-tRNA synthetase activity and (b) enhancing the expression of AARS1 to increase the activities of both lactyltransferase and alanyl-tRNA synthetase. We showed that lactate regulation of AARS1 expression plays a more important role in this regulation.

Overall, the function fate of AARS1 as a lactyltransferase or a tRNA synthetase seems to be determined by the intracellular concentrations of lactate and l-alanine. In normal cells primarily relying on oxidative phosphorylation to generate ATP, the intracellular lactate concentration is relatively low, and AARS1 mainly functions as a tRNA synthetase. In proliferating cancer cells addicted to aerobic glycolysis, the intracellular lactate may accumulate to high levels and promote the expression of AARS1, which in turn increases the cellular activity of AARS1 as both lactyltransferase and a tRNA synthetase. Thus, it is most likely that the function fate of AARS1 may depend on the relative abundance of lactate versus alanine in a specific cellular compartment. In this regard, note that lactate not only increased the expression of AARS1, but also promoted its translocation into the nucleus.

### Competitive relationship between lactylation and ubiquitination of YAP-TEAD.

Our study identified K90 and K108 as the major lactylation residues of YAP and TEAD1, respectively. Previous studies have reported that YAP K90 and TEAD1 K108 were also sites for ubiquitination (30, 31). Here, we showed that lactylation and ubiquitination of YAP-TEAD1 are mutually exclusive and mostly occur in different cellular compartments. AARS1 mainly interacts with and lactylates YAP-TEAD1 in the nucleus in response to increased levels of intracellular lactate, while ubiquitination of YAP-TEAD1 mainly occurs in the cytoplasm. Note that the reciprocal inhibition between lactylation and ubiquitination of YAP-TEAD1 is not merely due to competition of the identical target sites, i.e., lysine residues, but also because of the subcellular localization of YAP-TEAD1. For example, lactylation of YAP inhibited its nuclear export by XPO1 and thus prevented its translocation into the cytoplasm for ubiquitination.

### Feedback regulation of AARS1 by YAP-TEAD1.

Hyperactivation of YAP has been frequently found in malignant tumors, and such hyperactivation has been extensively correlated with tumor growth (16, 18). It has been well established that hyperactivation of YAP promotes tumor cell proliferation. Meanwhile, studies also showed that YAP-TEAD1 can promote glucose uptake and aerobic glycolysis to produce more lactate (41–43). Yet it was unclear whether and how YAP hyperactivation is coupled to intracellular lactate and global protein lactylation. In this regard, we found AARS1 serving as a direct target gene of YAP-TEAD1. And a lactate treatment was observed to enhance the binding of YAP-TEAD1 onto the promoter region of AARS1, leading to its increased expression. Thus, we concluded that AARS1 and YAP-TEAD1 form a positive-feedback loop linking high levels of intracellular lactate with global protein lactylation and accelerated cell proliferation. In addition, previous studies indicated that YAP can be activated by OGT-mediated *O*-GlcNAcylation to sense cellular glucose levels (26, 44). Since glucose is metabolized to lactate during aerobic glycolysis, this dual sensing mechanism of glucose and lactate by YAP and AARS1, respectively, may further enforce the interpretation of metabolic and nutrient cues into tumor cell proliferation signals.

### Clinical implications and therapeutic targeting of the AARS1-YAP-TEAD1 axis.

Considering the hyperactivation of YAP in GC and other human cancers, tremendous efforts have been focused on developing therapeutic strategies targeting the Hippo-YAP signaling pathway (23, 24, 45). In our current study, AARS1 expression was found to be upregulated in tumor tissues from patients with GC and MNU-induced GC mouse models, and this upregulation was consistent with our findings that AARS1 served as a direct target gene of YAP-TEAD1. Moreover, elevated expression levels and gain-of-function mutation of AARS1 and increased lactylation levels of YAP-TEAD1 were found to be closely associated with poor prognosis for patients with GC. Here, we found that genetic depletion of AARS1 regressed GC cell growth. However, as AARS1 plays a fundamental role in tRNA aminoacylation and protein synthesis and its deficiency has been reported to be associated with neurological disorders and acute liver failure (46, 47), therapeutic targeting of AARS1 to treat GC warrants further investigations.

### Physiological function of AARS1-mediated lactylation.

We discovered that AARS1 is a lactyltransferase that utilizes lactate and ATP to catalyze lysine lactylation on both histones and nonhistone proteins, and emphasized its pathological role especially via lactylation of YAP-TEAD1 in a context of tumorigenesis. However, accumulating studies have shown that lactylation can influence various physiological processes as well, and may do so via epigenetic regulation and other mechanisms. For instance, lactate is produced via glycolysis in stimulated M1 macrophages, thus promoting histone lactylation (7). The H3K18lac mark exhibits enrichment on promoter regions of homeostatic genes, thereby activating their expression and facilitating the acquisition of M2-like characteristics to ultimately achieve a biological steady state (7). Moreover, histone lactylation also plays important roles in the process of embryogenesis (48, 49). Lactylation of histones on the promoter regions of genes related to zygotic genome activation seems to facilitate their expression and promote preimplantation embryo development (48). In addition to tumor cells, lactate can be generated through glycolysis and locally accumulated in various types of cells even under physiological state. Thus AARS1 may also play a crucial role in protein lactate in these cells to regulate a variety of biological processes.

### Conclusion and limitations.

Our study revealed a noncanonical function of AARS1, namely lactyltransferase activity. In the case of AARS1-mediated lactylation of the Hippo pathway, AARS1 and YAP-TEAD form a positive-feedback loop that constitutively pushes forward the conversion of lactate metabolism into tumor cell growth. Meanwhile, our study still has limitations. For example, the clinical relevance of AARS1 to various GC subtypes as well as to other types of malignant tumors remains to be clarified. Moreover, considering the pivotal role of AARS1 in tRNA aminoacylation and protein synthesis, further investigations are warranted to explore the potential of targeting the lactyltransferase activity of AARS1 for the treatment of GC and other human malignancies. In addition to YAP-TEAD1, other substrates of AARS1 have not yet had their functions fully characterized. Also, the cell type–specific substrate spectrum for AARS1 warrants further investigation. In this regard, given the accumulation of lactate in the tumor microenvironment, protein lactylation in immune cells and tumor-associated fibroblasts is worthy of attention.

## Methods

A detailed description of materials and methods is provided in the supplemental material.

### Sex as a biological variable.

Our study examined male and female animals, and similar findings are reported for both sexes.

### Study approval.

All animal experiments were approved by the Institutional Animal Care and Use Committee of the Institute of Biochemistry and Cell Biology (Shanghai, China). The approval ID for the use of animals was SIBCB-NAF-14-004-S329-023. The gastric cancer tissue samples used in the study were derived from patients who gave written informed consent for the use of the specimen. The studies were performed in accordance with the Declaration of Helsinki and approved by the Hua’shan Hospital Institutional Review Board (Shanghai, China). The human gastric cancer tissue array was purchased from Shanghai Outdo Biotech.

### Data availability.

RNA-Seq and ChIP-Seq data reported in this paper were deposited in the NCBI’s Gene Expression Omnibus (GEO) database (GSE200850, GSE200789, and GSE200790). The human gastric cancer RNA-Seq data set used in Figure 5A was obtained from the GEO database with accession number GSE13911. Values for all data points in graphs are reported in the Supporting Data Values file. All unique/stable reagents generated in this study are available from the corresponding author with a completed material transfer agreement.

## Author contributions

JJ and HZ performed most of the experiments. ZY performed cellular assay. LA performed ChIP-Seq. YT and LT performed in vitro biochemical experiments. ML, JY, ZC, DG, FC, WW, and YH analyzed and discussed the data. ZZ, SJ, and JJ wrote the manuscript. ZZ, SJ, and SZ supervised the project. JJ is listed as the first author in recognition of his significant contribution to the inception of this study.

## Supplementary Material

Supplemental data

Unedited blot and gel images

Supporting data values

## Figures and Tables

**Table 1:**
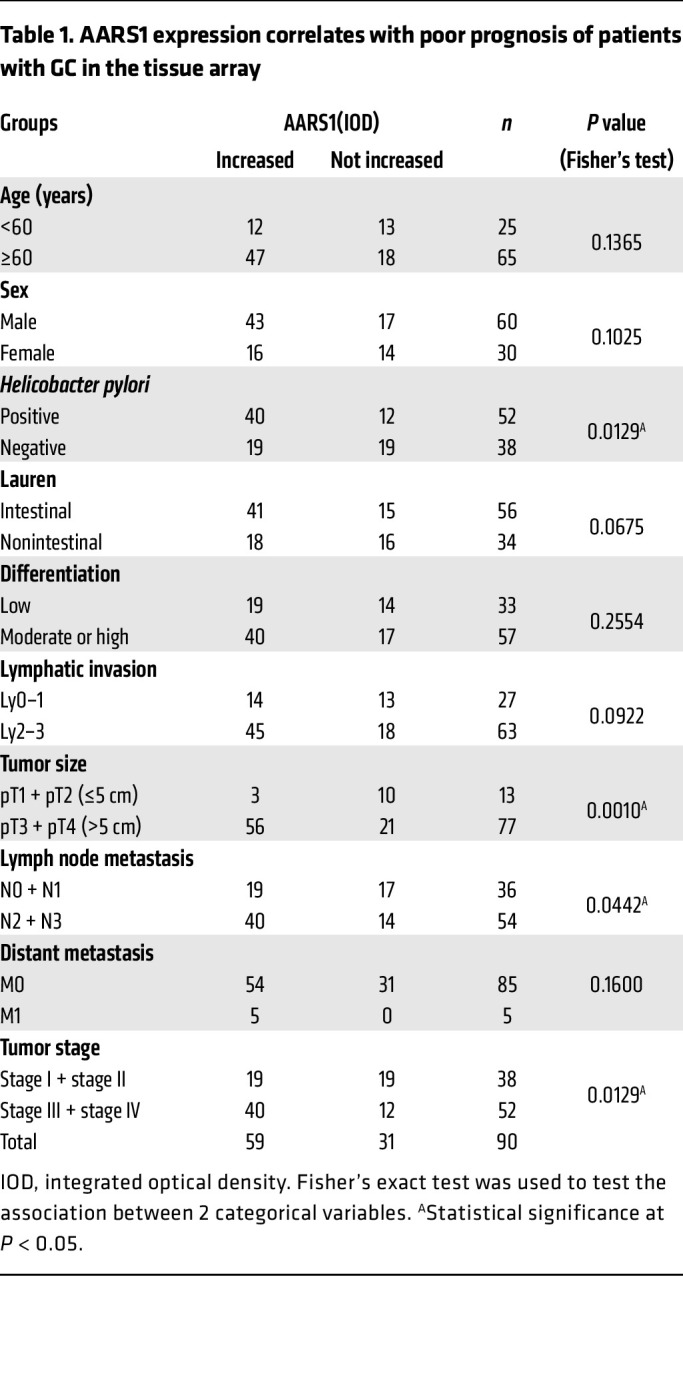
AARS1 expression correlates with poor prognosis of patients with GC in the tissue array

## Version 1 03/21/2024

In-Press Preview

## Version 2 05/15/2024

Electronic publication
