# Supplementary material for: The alanyl-tRNA synthetase AARS1 moonlights as a lactyltransferase to promote YAP signaling in gastric cancer
Source: J Clin Invest. 2024 Mar 21;134(10):e174587. doi: 10.1172/JCI174587 (PMC11093599; doi:10.1172/JCI174587)
Supplement: Supplemental data [file jci-134-174587-s262.pdf]

Supplemental Information

Supplemental Materials and Methods

Supplemental Figures 1~7

Supplemental Tables 1~4

Supplemental Materials and Methods

Cell culture

HGC27, AGS, HEK293FT and 293A cells were purchased from the cell library of the Chinese Academy of Sciences . HGC27 and AGS cells were maintained in RPMI 1640 medium (BasalMedia), and other cells were maintained in DMEM medium (BasalMedia). All cells were cultured in medium supplemented with 10% FBS (Biological Industries) and 1% penicillin/streptomycin (BasalMedia) at 37°C in a humidified atmosphere containing 5% CO_2. Cell line identity was confirmed by carrying out fingerprinting (Shanghai Genening Biotechnologies Inc.) for the HGC27 and AGS cell lines.

Plasmid and lentivirus packaging

For protein expression in *E. coli*, TEAD1, YAP (1-291), H3, H4, AARS1 full length and its catalytic domain (1-455) were cloned into modified pET28a vectors with indicated tags. For transient overexpression in HEK293FT and 293A cells, Flag, HA or Myc-tagged YAP, TEAD1, AARS1, AARS2 and XPO1 were cloned into the pCDNA 3.1 vector. For lentiviral-vector-mediated overexpression, YAP, TEAD1, and AARS1 were cloned into the pCDH-puro vector. Site-directed mutagenesis was used to generate mutant vectors of genes of interest. For AARS1 knockdown, designed shRNA oligonucleotides were inserted into the pLKO.1 vector. For p300 knockdown, designed siRNA oligonucleotides was synthesized by GenePharma. For lentiviral-vector-mediated AARS1, YAP and TEAD1 knockout, designed sgRNA

oligonucleotides from GeCKOv2 library were inserted into the LentiCRISPRv2 vector. The shRNA, siRNA and sgRNA sequences are listed in **Supplemental Table 3**.

Lentivirus was generated by co-transfecting lentiviral vector and packaging vectors psPAX2 and pMD2.G into HEK293FT cells. At 48 h after transfection, the conditioned medium was harvested and filtered through a 0.45 μm filter. Target cells were transduced with this conditioned medium in the presence of 8 μg/ml polybrene for 48 h and subjected for 5 μg/ml puromycin selection.

Lactylation proteomics analysis

HGC27 cells were cultured in glucose-free DMEM medium supplemented with 25 mM lactate for 12 h before harvest. The collected cells were washed with ice-cold phosphate-buffered saline and resuspended in a lysis buffer (8 M urea, pH 7.4, 3 μM TSA, 50 mM NAM and 1% protease inhibitor cocktail) for sonication on ice. The supernatants were collected after centrifugation at 12,000 g at 4°C for 15 min and the protein concentration was determined by Bradford assay. The protein sample was subjected to TCA precipitation and tryptic digestion overnight (trypsin:protein = 1:50, w/w). The digested peptides were dissolved in NETN buffer (50 mM Tris HCl, pH 7.6, 100 mM NaCl, 1 mM EDTA, and 0.5% NP-40) and incubated with anti-pan-Klac beads (PTM BIO, PTM-1404) at 4°C overnight. The beads were washed four times with NETN buffer and twice with ddH_2O. The peptides on the beads were eluted with 0.1% (v/v) TFA for three times and then dried in Thermo Fisher Scientific SPD210

SpeedVac Vacuum Concentrators (Thermo Fisher Scientific). The enriched peptides were subjected for HPLC-MS/MS analysis of lysine lactylation with a mass shift of +72.021 Da.

Cell growth, EdU labelling, and clonogenic assays

For cell growth assay, cells (2,000 per well) were seeded into 96-well plates overnight for attachment and then cultured in glucose-free DMEM medium (BasalMedia) with exogenous lactate (0, 5, 25 mM). Subsequently, cell growth was assessed at 24 h, 48 h, and 72 h after treatment using an ATP-based CellTiter-Lumi Plus kit (Beyotime) according to the manufacturer’s instructions. The intracellular ATP contents were measured using a BioTek SynergyTM NEO multi-detector microplate reader (Thermo Fisher Scientific). Cell growth was calculated using the equation Fold change = value (test) / value (0 h).

For EdU labelling, cells (1×10^5 per well) were seeded into 12-well plates for attachment, and then cultured in glucose-free DMEM medium (BasalMedia) with exogenous lactate (0, 5, 12.5, 25 mM) for 12 h. Subsequently, cells were incubated in EdU solution (10 μM, Beyotime) for 2 h and stained using the BeyoClick EdU-488 kit (Beyotime) according to the manufacturer's instructions. DAPI was used to dye the DNA in the cells, and the cells were visualized using a YueHe YHF40 inverted fluorescence microscope (YueHe) with 10 × objective lens.

For the clonogenic assay, isolated cells (600 per well) were seeded into a 6-well plate. After 14 days of incubation, cell colonies were stained with 0.5%

crystal violet dye and visualized using a YueHe YHF40 inverted microscope (YueHe) with 10 × objective lens.

RNA extraction, real-time qPCR, and RNA sequencing

Total RNA from cultured cells was extracted using RNA Isolater Total RNA Extraction Reagent (Vazyme) and then reverse transcribed to cDNA using HiScript II 1st Strand cDNA Synthesis Kit (Vazyme). Real-time qPCR was performed using a QuantStudio Real-time PCR system (Life Technologies) with SYBR Green qPCR master mix (Yeasen). The fold change in the gene expression was calculated using the comparative Ct method, and three replicates were tested for each cDNA sample. *GAPDH* was used as the internal reference. Primers used in this study are listed in **Supplemental Table 4.**

For RNA sequencing, HGC27 cells were transfected with scrambled siRNA (n.c.) or AARS1 siRNA at the final concentration of 50 nM for 48 h. Total RNA samples were then prepared from three biological replicates and sequenced using the Illumina NovaSeq 6000 platform of Majorbio Biotech. The data were analyzed on the free online Majorbio I-Sanger Cloud Platform.

Luciferase reporter assay

HEK293A cells were transfected with constructed luciferase reporter vectors, renilla luciferase vector, and YAP-TEAD1 overexpression vectors. Forty-eight hours after transfection, cell luciferase activities were measured using a Dual-luciferase Reporter Assay System (Promega) according to the manufacturer’s instructions. Relative luciferase activity was calculated as normalization of

Firefly to Renilla luciferase activity.

Immunoprecipitation and immunoblotting

Cellular proteins were extracted by lysis buffer (50 mM Tris HCl, pH 8.0, 1% NP-40, 2 mM EDTA, 150 mM NaCl, 1 mM PMSF), and incubated with anti-Flag, anti-HA-beads or with indicated antibodies and protein A/G beads (Santa Cruz) at 4°C overnight. Beads were then washed three times with the lysis buffer, the immunoprecipitates were eluted and boiled with SDS loading buffer and resolved in SDS-PAGE gels. The proteins were transferred to a PVDF membrane (Amersham) and further incubated with indicated primary antibodies. The immunoblotting images were scanned using a Mini Chemiluminescent Imaging and Analysis System (Sage Creation Science).

The primary antibodies and dilutions used in immunoblotting were: YAP (Santa Cruz, sc-101199, 1:1000), p-YAP (Cell Signaling Technology, 4911, 1:1000), MST1 (Cell Signaling Technology, 3682, 1:1000), LATS1 (Cell Signaling Technology, 9153, 1:1000), TEAD1 (ABclonal, A5218, 1:1000), pan-Klac (PTM BIO, PTM-1401, 1:1000), AARS1 (Proteintech, 17394-1-AP, 1:1000), p300 (Santa Cruz, sc-48343, 1:1000), Flag (Sigma-Aldrich, F3165, 1:5000), HA (Cell Signaling Technology, 3724, 1:1000), Lamin A/C (Proteintech, 10298-1-AP, 1:1000), Myc (Cell Signaling Technology, 2272, 1:1000), α-tubulin (Sigma-Aldrich, T6074, 1:5000), and β-actin (Sigma-Aldrich, A2228, 1:5000). The polyclonal antibody recognizing YAP lysine 90 lactylation (lacYAPK90, 1:1000) was generated using a K90-lactylated YAP peptide (RLRK^{lac}LPDSFFKPPC,

PTM BIO).

Immunofluorescence assay

For immunofluorescence assay, cells were seeded in confocal dishes (NEST, 801001) for attachment, and then fixed with 4% formaldehyde for 24 h. After permeabilization with 0.1% Triton X-100, the cells were blocked with 3% BSA in a solution of 0.01% Tween-20 in PBS (PBST) for 1 h and subsequently incubated with primary antibodies against YAP (Santa Cruz, sc-101199, 1:100), AARS1 (Santa Cruz, sc-165990, 1:100), Flag (Sigma-Aldrich, F3165, 1:1000), and HA (Cell Signaling Technology, 3724, 1:200) overnight at 4°C. Following incubation with AlexaFluor plus 488 goat anti-mouse IgG (Thermo, A32723, 1:400) and AlexaFluor plus 568 goat anti-Rabbit IgG (Thermo, A11011, 1:400), cell images were captured using a Zeiss LSM880 confocal microscope with 63 × oil immersion lens. DAPI (Sigma, D9542, 1:2000) was used to stain nuclear DNA.

For fluorescent immunohistochemical staining, gastric tissues were fixed with 4% paraformaldehyde (PFA), and were then sectioned into 3 μm-thick sections. Following antigen retrieval with a retrieval buffer (10 mM sodium citrate, pH 6.0), the tissue sections were incubated with primary antibodies and secondary fluorophore-conjugated antibodies. Tissue lactate staining was performed according to a previously reported protocol (1). Briefly, cryosections of frozen gastric tissue specimens were immersed in a reaction mixture containing lactate-detecting enzymes (Sigma-Aldrich, MAK064). The lactate in

the tissue section underwent enzymatic reaction and was converted into a fluorometric signal, which is proportional to the local lactate concentration. Finally, lactate staining was viewed by an Olympus FV3000 confocal microscopy and analyzed with the Imaris software (Version 9.0.1).

Chromatin immunoprecipitation (ChIP) assay and ChIP-Seq

Cells were cross-linked with freshly prepared formaldehyde (final concentration 1.42%) for 15 min, followed by addition of glycine (125 mM) for 5 min at room temperature. After two-round wash with cold PBS, cells were scraped and collected by centrifuge. Pelleted cells were resuspended in 400 μl ChIP lysis buffer (50 mM HEPES/KOH, pH 7.5; 140 mM NaCl; 1 mM EDTA; 1% Triton X-100; 0.1% Na-deoxycholate and protease inhibitors) and subjected to sonication with Qsonica Q800R3 sonicator (Qsonica) to shear the chromatin (high power, 30s on and 30 s off, 20 cycles). After sonication, samples were further diluted twice with lysis buffer and centrifuged to clear the supernatant. Eighty microliters of supernatant (1/10) were directly processed to extract total DNA as whole cell input. The remaining supernatants were transferred to new tubes and were subsequently incubated with either IgG or the indicated antibodies at 4°C overnight. Samples were added with prewashed protein A/G agarose beads (Santa Cruz, sc-2003) for another 3 h. After that, samples were washed five times with indicated buffers and were mixed with 100 μl of 10% chelex (Roche). The samples were boiled for 10 min and centrifuged at 4°C for 1 min. Supernatants were transferred to new tubes. After that, another 120 μl

of MilliQ water was added to each beads pellet, vortex for 10 s, and were centrifuged again to spin down the beads. The supernatants were combined as templates for follow-up qPCR analysis. The primers used in this study were listed in **Supplementary Table 4**.

For the ChIP-Seq assay, HA-YAP and Flag-TEAD1 transfected HEK293FT cells were resuspended in 400 μl of ChIP digestion buffer (20 mM Tris HCl, pH 7.5, 15 mM NaCl, 60 mM KCl, 1 mM $CaCl_2$ and protease inhibitors). To shear chromatin, cells were incubated with digestion buffer containing micrococcal nuclease (MNase, NEB, M0247S) at 37°C for 20 min. The reaction was then stopped with 2× stop buffer (100 mM Tris HCl, pH 8.1, 20 mM EDTA, 200 mM NaCl, 2% Triton X-100, 0.2% Na-deoxycholate and protease inhibitors). After sonication, the samples were immunoprecipitated with ChIP-grade antibodies against HA (Cell Signaling Technology, 3724) and Flag (Sigma-Aldrich, F3165) antibodies at 4°C overnight together with prewashed protein A/G beads. Subsequently, samples were eluted and reverse cross-linked in elution buffer (10 mM Tris HCl, pH 8.0, 10 mM EDTA, 150 mM NaCl, 5 mM DTT and 1% SDS) at 65°C overnight. After being sequentially digested with proteinase K (NEB, P8107S), DNA was purified using a PCR purification kit (Sangon Biotech, B518141). ChIP-derived DNA from three immunoprecipitations was pooled to generate libraries by using the Ovation Ultra-Low Library Prep kit (NuGEN) according to the manufacturer’s instructions. Sequencing was performed using an Illumina HiSeq2500 platform (APExBIO Technology LLC).

TurboID

TurboID assay were performed based on a previous protocol with minor modifications (2). Briefly, HEK293FT cells were transiently transfected with AARS1-TurboID vectors for 48 h. The transfected cells were then incubated with DMEM medium supplemented with 200 μM biotin (Sigma-Aldrich, B4639) for 2 h at 37°C. The incubated cells were lysed with lysis buffer (50 mM Tris HCl, pH 8.0, 1% NP-40, 2 mM EDTA, 150 mM NaCl, 1 mM PMSF) supplemented with micrococcal nuclease (NEB, M0247S, 1:5000) on ice for 15 min. Subsequently, cell lysate was centrifuged at 12,000 rpm at 4°C for 10 min to collect supernatant. The supernatant was incubated with streptavidin beads (Smart-lifesciences, SA021005) at 4°C for 2 h. The beads were washed with lysis buffer for three times as samples for 4D label-free mass spectrum analysis (Majorbio).

Recombinant protein purification

The recombinant proteins were expressed in *Escherichia coli* BL21(DE3) strain. Protein expression in BL21 cells (OD_{600} = 0.6) was induced by isopropyl-β-D-thiogalactopyranoside (IPTG) at a final concentration of 0.5 mM at 25°C for 20 h. Collected bacteria were lysed in lysis buffer (20 mM Tris HCl, pH 7.5, 500 mM NaCl, 10% glycerol and 1 mM DTT) with a high-pressure homogenizer, followed by a centrifugation at 15,000 g for 30 min at 4°C. The supernatants were incubated with GST, MBP, or Ni-NTA agarose beads (Yeasen) for affinity chromatography according to the manufacturer's instructions. Protein purity

was examined by Coomassie brilliant blue (CBB) staining and protein concentrations were determined by Bradford protein assay.

Pulldown assay

GST-H3, GST-H4, GST-AARS1 or MBP-YAP proteins immobilized on MBP/GST beads were incubated with indicated prey proteins in a binding buffer (20 mM HEPES, pH 7.5, 100 mM NaCl) overnight at 4°C. After being washed three times, the beads were eluted with the binding buffer containing 10 mM maltose monohydrate or 10 mM glutathione. Eluted proteins were analyzed using SDS-PAGE and subsequent CBB staining.

Isothermal titration calorimetry (ITC)

ITC experiments were conducted using an iTC200 microcalorimeter (Microcal) at 25°C. For calorimetric measurements, purified AARS1 was loaded into the ITC cell at a concentration of 30 μM, and 300 μM lactate or L-alanine in 50 mM Tris HCl and 150 mM NaCl were auto-loaded into the syringe. Each titration included a single 0.4 mL injection followed by 19 sequential injections of 2 mL aliquots, with a spacing of 300 s between the injections, and stirring at 1,000 rpm. Data were analyzed using the ORIGIN data analysis software (MicroCal Software).

Gel shift assay

Gel shift assay were performed based on a previous protocol with minor modifications (3). Briefly, purified TEAD1 and YAP (1-291) proteins were incubated with double-stranded DNA oligonucleotides (5’-

CAGACAGGGCCTCGCTCTGTCGCCCAAGGAATGCAGTGTCACGATCAGG GCTCACTGCAG-3'), corresponding to the sequences containing TEAD1 binding site on the promoter of *AARS1*, in the binding buffer (10 mM Tris HCl, pH 7.5, 50 mM KCl, 1 mM DTT, 2% Glycerol) at 25°C for 1 h. The reaction samples were analyzed with a 5% native polyacrylamide gel in Tris-Glycine buffer (25 mM Tris HCl, pH 8.3, 192 mM Glycine) and viewed using ethidium bromide staining.

***In vitro* lactylation assay**

In vitro lactylation assay were performed in a 20 µl reaction mixtures containing 50 mM Tris HCl, pH 7.6, 10 mM $MgCl_2$, 20 mM KCl, 10 mM lactate, 10 mM ATP, 0.5 mg/ml recombinant $AARS1^{455}$ and 0.5 mg/ml TEAD1, GST-H3, GST-H4, 0.5 mg/ml synthetic H3K18 peptide (APRKQLAT), TEAD1 K108 peptide (RDFHSKLKDQTC) or YAP K90 peptide (PMRLRKLPDSFC) at 37°C for 1 h. Protein samples were boiled in 5× SDS loading buffer and the lactylation levels were analyzed by immunoblotting using pan-Klac antibody (PTM BIO, PTM-1401, 1:1000). H3K18, TEAD1 K108 and YAP K90 peptide was desalted using C18 ZipTip (Millipore) and subjected for mass spectrum analysis using a MALDI-TOF/TOF mass spectrometer (SCIEX-5800). Peptide lactylation exhibited a +72.021 Da mass shift.

***In vitro* delactylation assay**

In vitro delactylation assay were performed in 20 µl reaction mixtures containing 50 mM Tris HCl, pH 7.6, 6 mM $MgCl_2$, 1 mM DTT, 1 mM NAD^+, 1 mg/ml

recombinant SIRT1, 1 mM PMSF and 0.5 mg/ml synthetic lactylated TEAD1 K108 peptide (RDFHSK^{lac}LKDQTC) or YAP K90 peptide (PMRLRK^{lac}LPDSFC) at 37°C for 1 h. The peptides were desalted using C18 ZipTip and subjected for mass spectrum analysis using a MALDI-TOF/TOF mass spectrometer (SCIEX-5800).

PPi measurement

PPi levels in *in vitro* lactylation assay was measured using a commercial Fluorimetric Pyrophosphate assay kit (Yeasen) according to the manufacturer's instructions. Lactylation reactions were carried out in a 20 μl mixtures containing 50 mM Tris HCl, pH 7.6, 10 mM $MgCl_2$, 20 mM KCl, 0-10 mM lactate, 10 mM ATP, 0.5 mg/ml recombinant AARS1 and 0.5 mg/ml TEAD1 K108 peptide.

Immunohistochemistry staining

A tissue array of 90 gastric cancer specimens, each one paired with a normal specimen, was obtained from Shanghai Outdo Biotech (Cat# HStmA180Su10). The follow-up time for the GC patients ranged from Dec. 2009 to Jun. 2010. Data on clinicopathological features and prognoses of the patients were collected and analyzed retrospectively. The studies were performed in accordance with the Declaration of Helsinki and approved by the Hua'shan Hospital Institutional Review Board (HIRB). Patients' characteristics are shown in **Table 1**. The microarray sections were used for AARS1 IHC staining analysis with AARS1 antibody (Santa Cruz, sc-165990, 1:50), YAP antibody (Santa Cruz,

sc-101199, 1:50), and TEAD1 antibody (ABclonal, A5218, 1:50). The results were analyzed with Image Scope software.

The integrated optical density (IOD, H-score) of the immunostaining was also measured by using Image-Pro Plus Version 6.2 software (Media Cybernetics Inc, Bethesda, MD), and the cutoff value for the definition of subgroups was the median IOD. The IOD > median was considered as high expression group, and IOD ≤ median was considered as low expression group.

Animal models

All animals were housed in ventilated cages under specific pathogen-free conditions with a 12 h light/dark cycle and ad libitum access to food and water. Healthy female BALB/c nude mice (6-week-old) were allowed to acclimate to these conditions for 1 week before being used. During the tumor formation assay, HGC27 cells (1×10^6) were subcutaneously injected into the left flank or orthotopically injected into the stomach of each tested mouse. Mice were sacrificed four weeks after injection of tumor cells, and tumor volumes were then measured.

To establish a N-Nitroso-N-methylurea (MNU)-induced GC mouse model, 8-week-old C57BL/6J mice received three cycles of MNU (TRC, Toronto, Canada) in drinking water. For each cycle, drinking water containing 240 μg/ml MNU was served for the mice for one week, and then normal drinking water was served next week. After 24 weeks, mice were scarified for subsequent analysis.

Statistics

In general, at least two independent experiments were performed with similar results. No data were excluded from the analysis. No statistical methods were used to predetermine sample sizes and sample sizes were chosen empirically and are similar to those reported in previous studies. Experiments included a minimum of three replicates as indicated in the figure legends. Data distribution was assumed to be normal but this was not formally tested. For *in vivo* experiments, all mice were randomly allocated into different experimental groups. For *in vitro* studies, no randomization was performed. The investigators were not blinded to allocation during experiments and outcome assessment.

Estimations of sample size for planned comparisons of two independent means were undertaken using GraphPad Prism 9. Data are shown as mean ± S.D. for continuous variables and as frequencies and proportions for categorical variables. Continuous data were compared using Student's t-tests (when comparing two variables) or one-way ANOVA (when comparing three or more variables). To determine correlations, the Spearman rank correlation was used for continuous variables. Survival curves were calculated according to the Kaplan-Meier method; survival analysis was performed using the logrank test. A value of $p < 0.05$ was considered to indicate a significant difference.

Study approval

All animal experiments were approved by the Institutional Animal Care and Use Committee of the Institute of Biochemistry and Cell Biology. The approval ID for

the use of animals was SIBCB-NAF-14-004-S329-023. Tumor volume was determined by taking caliper measurements of tumor length, width, and depth and using the equation volume = 0.5236 × length × width × depth (mm3). The maximal tumor size of animal experiments in this study was 2,000 mm^3. The gastric cancer tissue samples used in the study were derived from patients who signed informed consents for the use of the specimen. The studies were performed in accordance with the Declaration of Helsinki and approved by the Hua'shan Hospital Institutional Review Board (HIRB). The human gastric cancer tissue array was purchased from Shanghai Outdo Biotech.

References

1. Walenta S, Schroeder T, and Mueller-Klieser W. Metabolic mapping with bioluminescence: basic and clinical relevance. *Biomol Eng.* 2002;18(6):249-62.
2. An L, Cao Z, Nie P, Zhang H, Tong Z, Chen F, et al. Combinatorial targeting of Hippo-STRIPAK and PARP elicits synthetic lethality in gastrointestinal cancers. *J Clin Invest.* 2022;132(9).
3. Ju J, Wang Y, Liu R, Zhang Y, Xu Z, Wang Y, et al. Human fetal globin gene expression is regulated by LYAR. *Nucleic Acids Res.* 2014;42(15):9740-52.

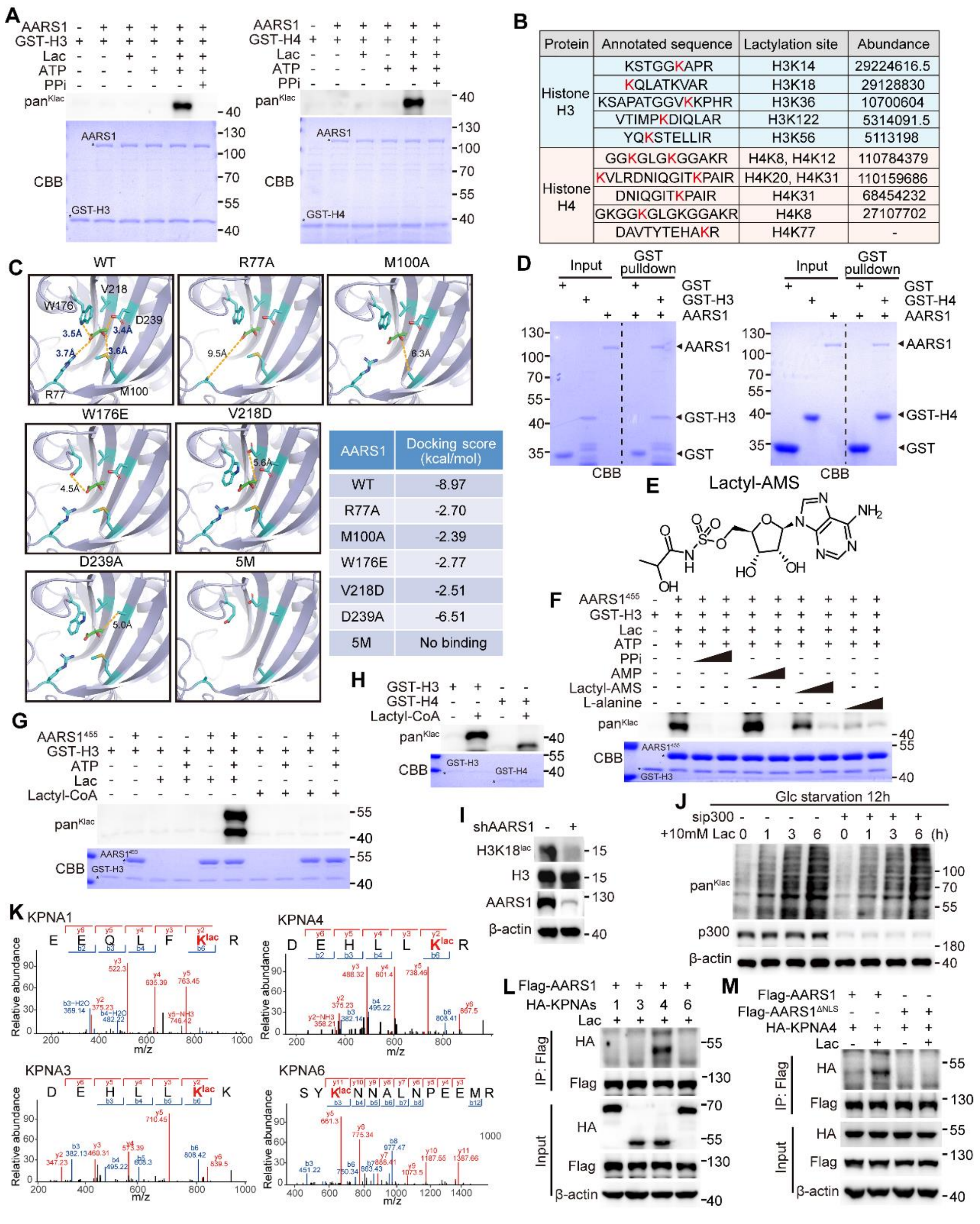

Supplemental Figure 1. AARS1 is a lactyl-transferase

(A) Immunoblotting with pan-Klac antibody to detect AARS1-induced lactylation of GST-H3 and GST-H4 *in vitro*. Coomassie brilliant blue (CBB) staining showing the purified full-length AARS1, GST-H3 and GST-H4 used in *in vitro* lactylation assay. Asterisks represent the AARS1, GST-H3 and GST-H4 proteins. The final concentrations of PPi in the reaction

mixture was 10mM. **(B)** Identified lactylated lysine residues of H3 and H4 from in vitro lactylation assay. **(C)** Predicted interactions between lactate and the amino acid residues in the catalytic core of wildtype and mutant AARS1 (top). Calculated binding energy score of lactate binding to wildtype and mutant AARS1 (bottom). **(D)** GST-pulldown assay to detect the interaction between AARS1 and GST-H3 and GST-H4. Asterisks represent the indicated proteins. **(E)** Structure of synthetic lactyl-AMS that mimics lactyl-AMP intermediate. **(F)** Immunoblotting with pan-Klac antibody to detect $AARS1^{455}$-induced lactylation of GST-H3 *in vitro* in the presence of PPi, AMP, lactyl-AMS and L-alanine. Coomassie brilliant blue (CBB) staining showing the purified $AARS1^{455}$ and GST-H3 used in *in vitro* lactylation assay. Asterisks represent the $AARS1^{455}$ and GST-H3 proteins. The final concentrations of PPi, AMP, lactyl-AMS and L-alanine in the reaction mixture were 2mM and 10mM, respectively. **(G)** Immunoblotting with pan-Klac antibody to detect AARS1455-induced lactylation of GST-H3 *in vitro* in the presence of lactate or lactyl-CoA. Coomassie brilliant blue (CBB) staining showing the purified $AARS1^{455}$ and GST-H3 used in *in vitro* lactylation assay. Asterisks represent the $AARS1^{455}$ and GST-H3 proteins. The concentrations of lactate and lactyl-CoA in the reaction mixture were 10mM and 10μM, respectively. **(H)** Immunoblotting with pan-Klac antibody to detect lactylation of GST-H3 and GST-H4 *in vitro* with or without 0.4mM lactyl-CoA. Coomassie brilliant blue (CBB) staining showing the purified GST-H3 and GST-H4 used in *in vitro* lactylation assay. Asterisks represent the GST-H3 and GST-H4 proteins. **(I)** Histone H3K18 lactylation levels in AARS1-knockdown HGC27 cells. **(J)** Immunoblotting with pan-Klac antibody to detect global protein lactylation levels in glucose-deprived p300-knockdown HGC27 cells

stimulated with 10mM lactate for indicated times. **(K)** Mass spectra of lactylated sites of KPNAs (KPNA1, KPNA3, KPNA4 and KPNA6). **(L)** Co-immunoprecipitation analysis of the interactions between Flag-AARS1 and HA-KPNAs in HEK293FT cells cultured in glucose-free medium with 25 mM lactate. **(M)** Co-immunoprecipitation analysis of the interactions between KPNA4 and AARS1 or its ΔNLS mutant in HEK293FT cells cultured in glucose-free medium treated with or without 25 mM lactate.

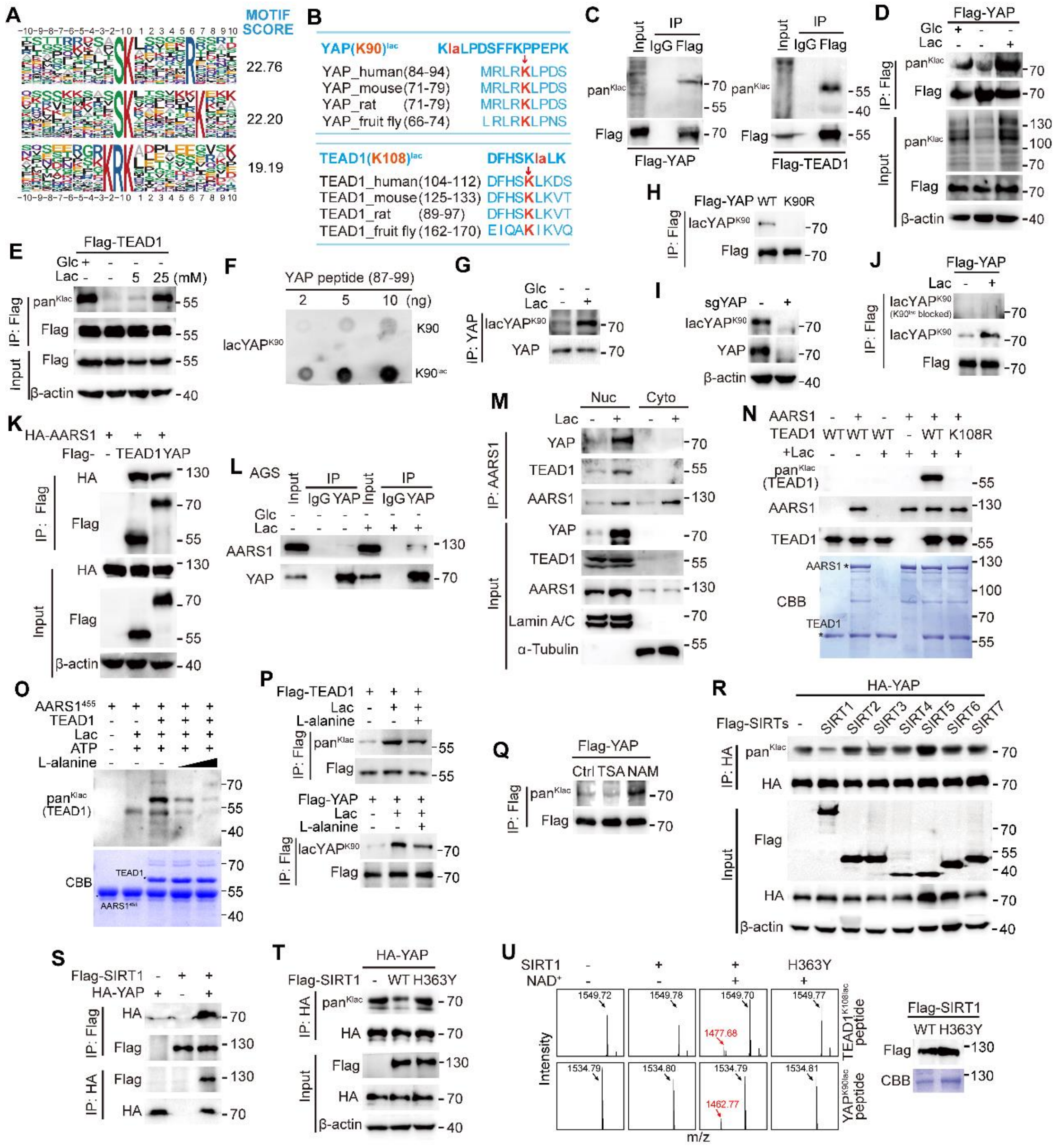

Supplemental Figure 2. AARS1 lactylates YAP-TEAD complex

(A) Top 3 motifs of lactylated lysine residues from lactylation proteomics. **(B)** Sequence alignment of lactylated sites of YAP and TEAD1 in the indicated species. **(C)** Lactylation levels of exogenous YAP and TEAD1 using pan-Klac antibody in HEK293FT cells transfected with the indicated plasmids. **(D)** Lactylation levels of exogenous YAP in lactate-stimulated HEK293FT cells. Glc, glucose. Lac, lactate. **(E)** Lactylation levels of exogenous TEAD1 in lactate-stimulated HEK293FT cells. Glc, glucose. Lac, lactate. **(F)** Dot blot

analysis using lacYAPK90 antibody to detect synthesized unmodified YAP peptide (K90) or K90-lactylated YAP peptide (K90lac) (amino acid residues 87 to 99). **(G)** Immunoblotting using lacYAPK90 antibody to detect the lactylation levels of YAP at K90 in the lactate-treated HGC27 cells. Lac, lactate. **(H)** Immunoblotting using lacYAPK90 antibody to detect the lactylation levels of YAP at K90 in HEK293FT cells transfected with YAP wildtype (WT) and its mutant (K90R). **(I)** Immunoblotting analysis of YAP K90 lactylation and YAP levels in YAP knockout cells. **(J)** Immunoblotting of YAP K90 lactylation in Flag-immunoprecipitates form Flag-YAP- overexpressing HEK293FT cells cultured in glucose-free medium with or without lactate. LacYAPK90 antibody was pretreated with or without YAP K90lac peptide. Lac, lactate. **(K)** Exogenous interaction of YAP and TEAD1 with AARS1 in HEK293FT cells transfected with the indicated plasmids. **(L)** Endogenous interaction between YAP and AARS1 in AGS cells treated with or without lactate. Glc, glucose. Lac, lactate. **(M)** Endogenous interaction of YAP and TEAD1 with AARS1 in the nuclear and cytosolic fractions of lactate-treated HGC27 cells. Lac, lactate. **(N)** *In vitro* lactylation assay with pan-Klac antibody to detect TEAD1 lactylation catalyzed by AARS1. Immunoblot and CBB stain analysis of the purified recombinant TEAD1 and AARS1 proteins used in the *in vitro* lactylation assay. Asterisks indicate purified recombinant AARS1 and TEAD1. **(O)** *In vitro* lactylation assay with pan-Klac antibody to detect AARS1-mediated TEAD1 lactylation in the presence of L-alanine. The final concentrations of PPi in the reaction mixture were 2 mM and 10 mM. **(P)** Lactylation levels of TEAD1 and YAP in TEAD1 and YAP-overexpressing cells treated with lactate and L-alanine. **(Q)** Lactylation levels of YAP in YAP-overexpressing cells treated with TSA (0.5 μM) or NAM (5 mM). **(R)**

Lactylation levels of YAP in HEK293FT cells co-transfected with HA-YAP and Flag-SIRTs. **(S)** Exogenous interaction between YAP and SIRT1 in HEK293FT cells transfected with the indicated plasmids. **(T)** Lactylation levels of YAP in HEK293FT cells transfected with the indicated plasmids. **(U)** Mass spectrometry to determine the delactylation of the synthetic lactylated YAP K90lac and TEAD1 K108lac peptides by SIRT1 and its catalytic-dead mutant (H363Y) *in vitro* (top). Immunoblotting and CBB staining of wildtype (WT) and H363Y SIRT1 used in the *in vitro* delactylation assay (bottom).

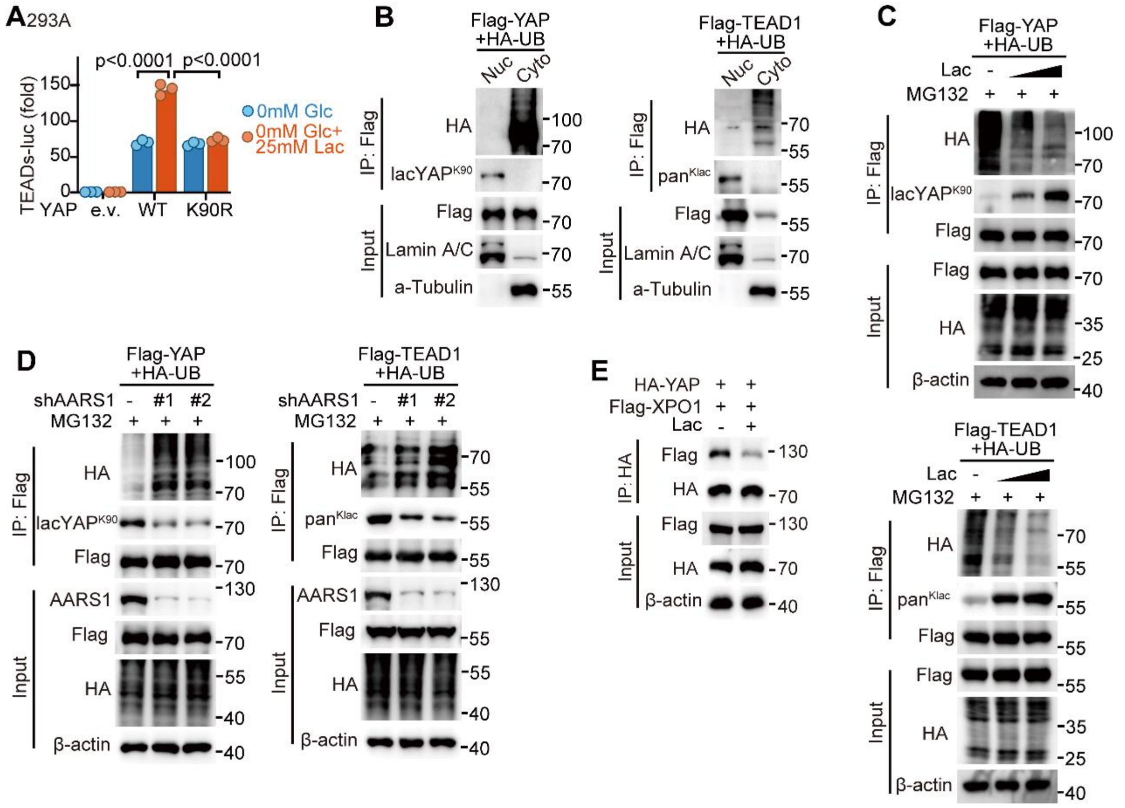

Supplemental Figure 3. AARS1-induced lactylation enhances nuclear trans-localization and stabilization of YAP-TEAD

(A) Relative TEAD4 luciferase activity in lactate-treated HEK293FT cells transfected with the indicated plasmids (*n* = 3). Data are presented as mean ± SD. **(B)** Nucleocytoplasmic distribution of lactylation and ubiquitination levels of YAP and TEAD1 in Flag-tagged YAP or TEAD1 overexpressing HEK293FT cells. Nuc, nuclear localization. Cyto, cytosolic localization. **(C)** Immunoblotting analysis of lactylation and ubiquitination levels of YAP and TEAD1 in lactate treated HEK293FT cells. Lac, lactate. **(D)** Immunoblotting analysis of lactylation and ubiquitination levels of YAP and TEAD1 in AARS1 knockdown HEK293FT cells. **(E)** Co-immunoprecipitation analysis of the interaction between exogenous YAP and XPO1 in HEK293FT cells with or without lactate stimulation. Unpaired Student's *t* test (**A**).

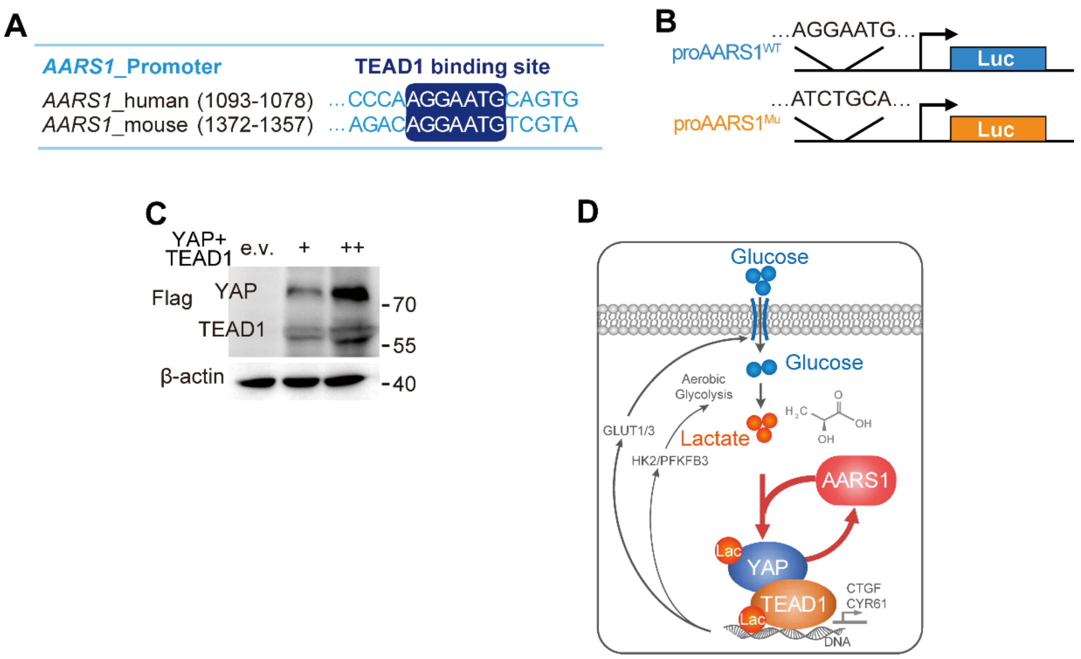

Supplemental Figure 4. A positive feedback loop of AARS1-YAP-TEAD axis

(A) Conserved TEAD1 binding site in the promoter region of the human and mouse *AARS1* genes. **(B)** Schematic representation of luciferase reporter constructs containing *AARS1* promoter with wildtype (WT) or mutant (Mu) TEAD1 binding site. **(C)** The protein levels of YAP and TEAD1 in HEK293FT cells transfected with the indicated plasmids. **(D)** Schematic illustration of the positive feedback loop between AARS1 and YAP-TEAD1 signaling.

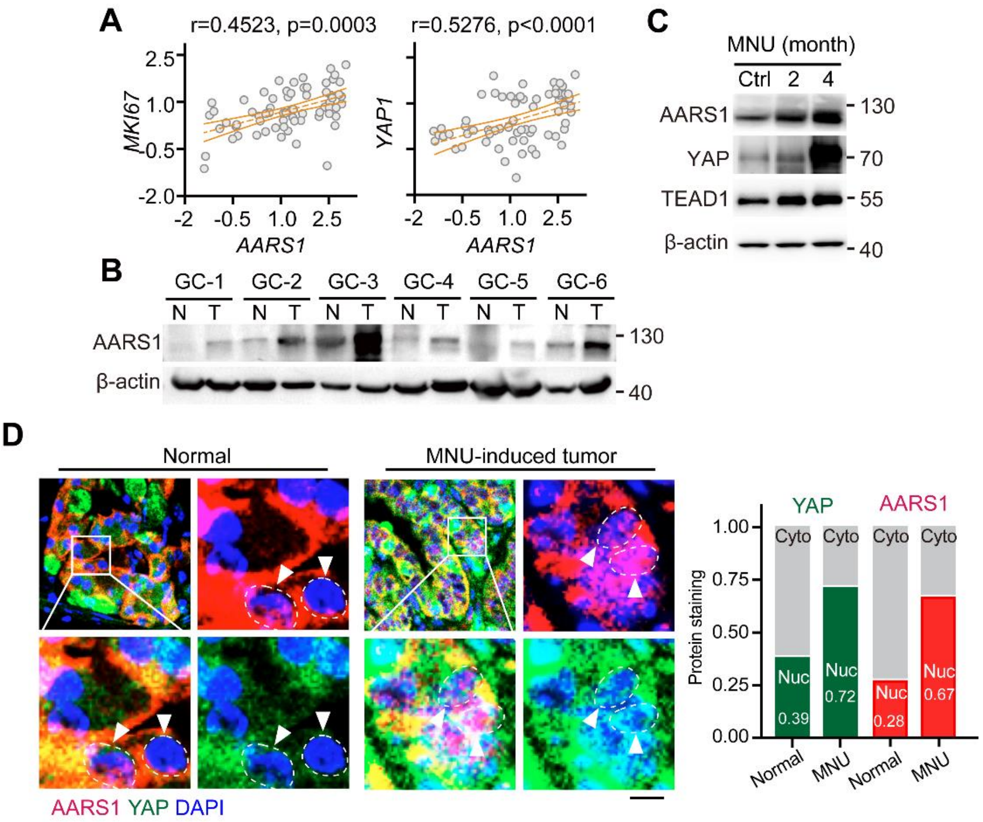

Supplemental Figure 5. AARS1 expression is upregulated in gastric cancer

(A) Pearson's correlation analysis to evaluate the association of *AARS1* transcription with *MKI67* (left) or *YAP1* (right) from GEO database, GSE13911. **(B)** Immunoblotting of AARS1 levels in human healthy and GC tissues. **(C)** Immunoblot analysis of AARS1, YAP, and TEAD1 levels in the tissues of the MNU-induced GC model at indicated times. **(D)** Immunohistofluorescence images of AARS1 and YAP in MNU-induced murine GC (left). Arrows and dash lines represented the staining of AARS1 and YAP in the nucleus. Quantification of AARS1 in the nuclear and cytosolic distribution was shown (right) ($n = 3$). Scale bar = 5 μm. Spearman rank correlation (**A**).

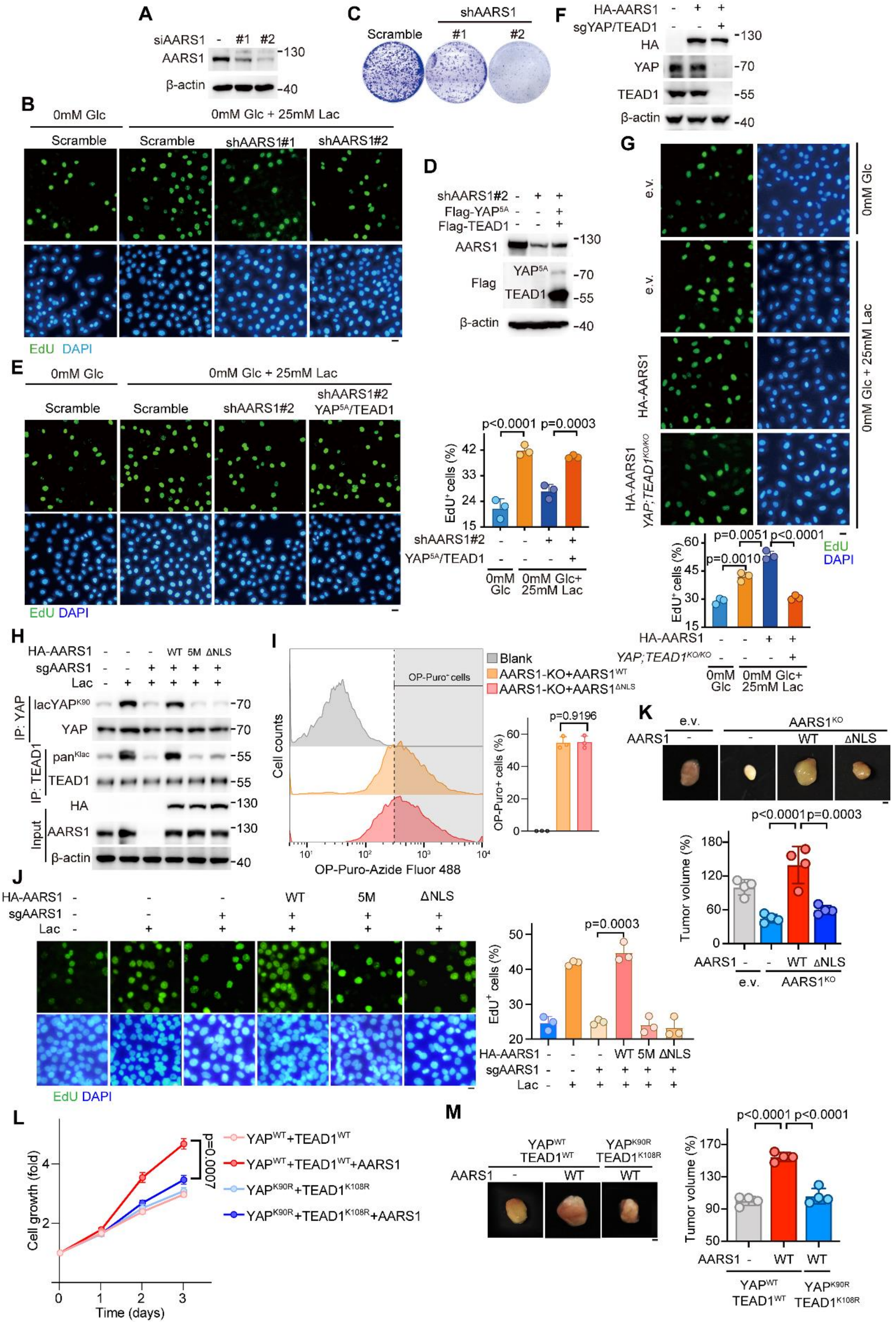

Supplemental Figure 6. AARS1 promotes gastric cancer via YAP-TEAD lactylation

(A) Knockdown efficiency of AARS1 siRNAs in HGC27 cells. **(B)** Representative images

of EdU-labelling cells in AARS1-knockdown cells treated with lactate for 12 h. Glc, glucose. Lac, lactate. Scale bar = 10 μm. **(C)** Colony formation assay of AARS1-knockdown AGS cells. **(D)** The protein levels of AARS1, YAP and TEAD1 in AARS1-knockdown AGS cells overexpressing YAP^{5A} and TEAD1. **(E)** Representative EdU staining in AARS1-knockdown cells overexpressing YAP^{5A} and TEAD1 (left). Percentage of EdU^{+} cells was measured (right) ($n = 3$). Scale bar = 10 μm. Data are presented as mean ± SD. **(F)** The protein levels of AARS1, YAP and TEAD1 in HA-AARS1-overexpressing and YAP-TEAD1-knockout AGS cells. **(G)** Representative EdU staining images of HA-AARS1-overexpressing and YAP-TEAD1-knockout cells upon lactate treatment (top). Percentage of EdU^{+} cells was measured (bottom) ($n = 3$). Scale bar = 10 μm. Data are presented as mean ± SD. **(H)** Immunoblotting analysis of YAP-TEAD1 lactylation in control, AARS1-knockout and AARS1 WT, 5M and ΔNLS mutants rescued AARS1-knockout cells in the presence of lactate. Lac, lactate. **(I)** Flow cytometry analysis of $OP\text{-}Puro^{+}$ cells in AARS1 WT or ΔNLS mutants rescued AARS1-knockout cells ($n = 3$). **(J)** Representative images of EdU staining of control, AARS1-knockout and AARS1 WT, 5M and ΔNLS mutants rescued AARS1-knockout cells treated with lactate (left). Percentage of EdU^{+} cells (right) ($n = 3$). Scale bar = 10 μm. Data are presented as mean ± SD. **(K)** Tumor growth of control, AARS1-knockout and AARS1 WT or ΔNLS mutants rescued AARS1-knockout cells in tumor xenograft model ($n = 4$). Scale bar = 2 mm. Data are presented as mean ± SD. **(L** and **M)** Cell growth curve ($n = 3$) (L) and tumor growth ($n = 4$) (M) of wildtype and lactylation-deficient mutants YAP-TEAD1-overexpressed cells in the presence of AARS1. Scale bar = 2 mm. Data are presented as mean ± SD. Unpaired Student's *t* test (**E**, **G**, **I**, **J**, **K**, and **M**). One-way ANOVA

(**L**).

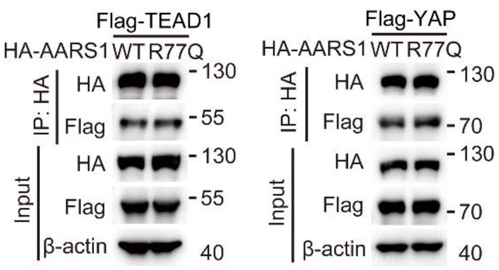

Supplemental Figure 7. R77Q mutation promotes AARS1 activity and GC cell growth

Co-immunoprecipitation analysis of the interaction between YAP-TEAD1 and WT or R77Q mutant AARS1.

Supplemental Table 1. AARS1 expression positively correlates with YAP expression in the tissue array for 90 GC patients

YAP / AARS1	Increased	Non-increased	Fisher's exact test
Increased	48	11	p=0.0125*
Non-increased	17	14	

Numbers of patients were listed with increased or non-increased expression of AARS1/ YAP in GC tissues compared to associated normal tissues.

* Represents statistical significance at $p < 0.05$.

Supplemental Table 2. AARS1 expression positively correlates with TEAD1 expression in the tissue array for 90 GC patients

AARS1 \ TEAD1	Increased	Non-increased	Fisher's exact test
Increased	41	18	p = 0.0397*
Non-increased	14	17	

Numbers of patients were listed with increased or non-increased expression of AARS1/ TEAD1 in GC tissues compared to associated normal tissues.

* Represents statistical significance at $p < 0.05$.

Supplemental Table 3. siRNA, shRNA and sgRNA sequences

Target gene	Sequence (5'-3')
siAARS1-1	GGUGGAUGACAGCAGUGAAGA
siAARS1-2	GGACAUCAUUAAUGAAGAAGA
siYAP	GGUGAUACUAUCAACCAAAUU
sip300	CCAAGUGGCACACUGUGCAUCUUCU
shAARS1-1	GGTGGATGACAGCAGTGAAGA
shAARS1-2	GGACATCATTAATGAAGAAGA
sgYAP	ATTATAGGTCCTCTTCCTGA
sgTEAD1	GGGACACTCACCTGGAATGG
sgAARS1-1	CCACGCTCGGACCATCACTG
sgAARS1-2	TGGGGGCAAACATAATGACC

Supplemental Table 4. Primers used in real-time qPCR and ChIP qPCR

Real-time qPCR primers		
Gene	Forward primer	Reverse primer
GAPDH	GAAGGTGAAGGTCGGAG	GAAGATGGTGATGGGATTTC
CTGF	AAAAGTGCATCCGTACTCCCA	CCGTCGGTACATACTCCACAG
CYR61	GGTCAAAGTTACCGGGCAGT	GGAGGCATCGAATCCCAGC
AARS1	TCACCCAAGAGTTTGGCATTC	CCTGGGAGGATTTTGGTGTCA
YAP	TAGCCCTGCGTAGCCAGTTA	TCATGCTTAGTCCACTGTCTGT
ChIP qPCR primers		
Gene	Forward primer	Reverse primer
CTGF-pro	TGTGAGCTGGAGTGTGCCAG	AGGCTTTTATACGCTCCGGG
CYR61-pro	GCCAACCAGCATTCCTGAGA	GAGCCCGCCTTTTATACGGG
AARS1-pro	GGGACTCCAGCTGACCTCAT	AGGCATTGCATCAAACATTTCAC
