# Supplementary material for: The alanyl-tRNA synthetase AARS1 moonlights as a lactyltransferase to promote YAP signaling in gastric cancer
Source: J Clin Invest. 2024 Mar 21;134(10):e174587. doi: 10.1172/JCI174587 (PMC11093599; doi:10.1172/JCI174587)

pan^{Klac} for Fig1C

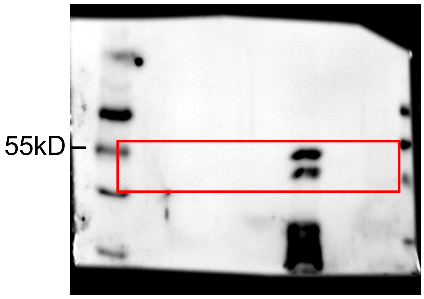

CBB for Fig1C

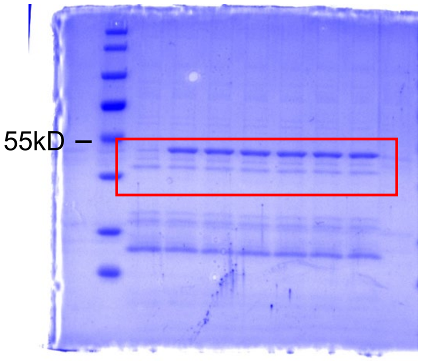

pan^{Klac} for Fig1C

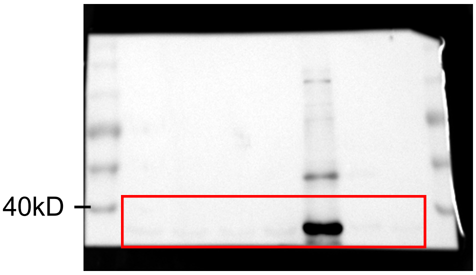

CBB for Fig1C

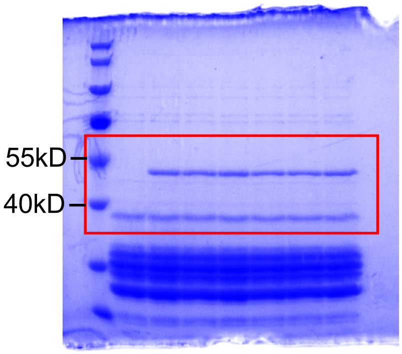

pan^{Klac} for Fig1G

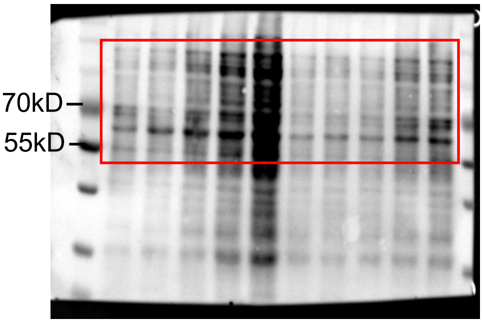

CBB for Fig1G

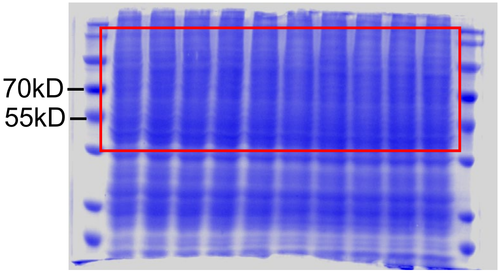

AARS1 for Fig1G

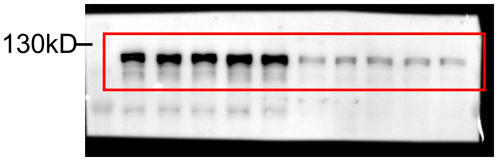

β-actin for Fig1G

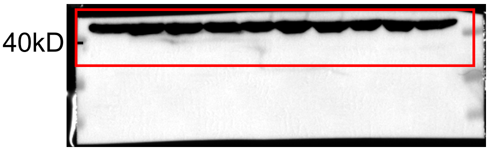

AARS1 for Fig1H

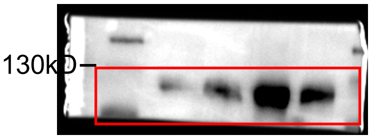

Lamin A/C for Fig1H

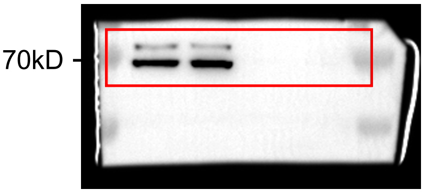

α-Tubulin for Fig1H

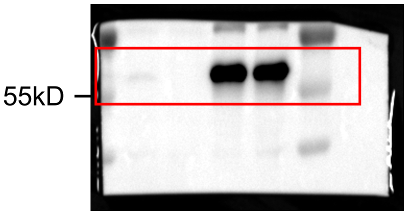

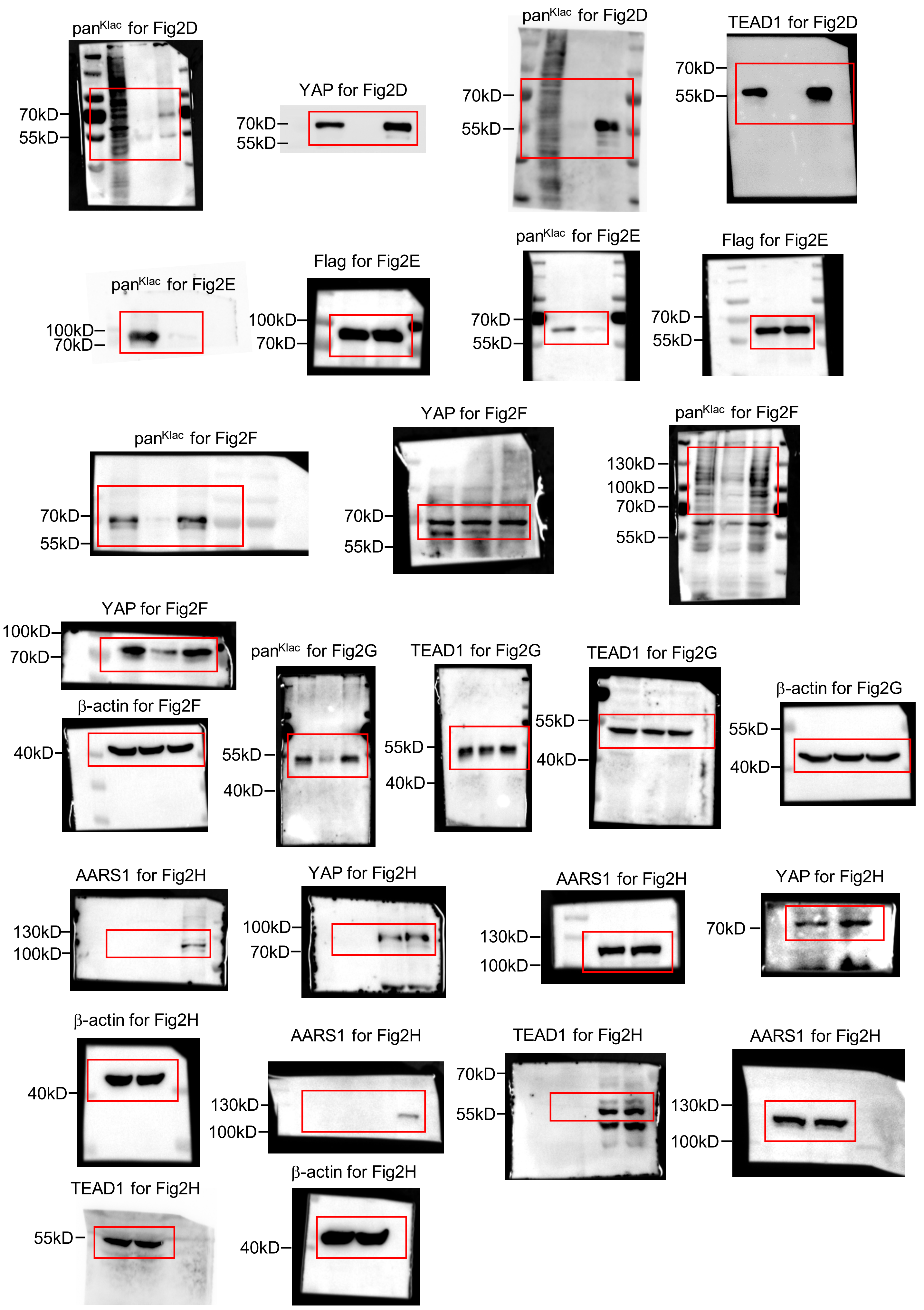

pan^Klac for Fig2D
70kD
55kD
YAP for Fig2D
70kD
55kD
pan^Klac for Fig2D
70kD
55kD
TEAD1 for Fig2D
70kD
55kD
pan^Klac for Fig2E
100kD
70kD
Flag for Fig2E
100kD
70kD
pan^Klac for Fig2E
70kD
55kD
Flag for Fig2E
70kD
55kD
pan^Klac for Fig2F
70kD
55kD
YAP for Fig2F
70kD
55kD
pan^Klac for Fig2F
130kD
100kD
70kD
55kD
YAP for Fig2F
100kD
70kD
β-actin for Fig2F
40kD
pan^Klac for Fig2G
55kD
40kD
TEAD1 for Fig2G
55kD
40kD
TEAD1 for Fig2G
55kD
40kD
β-actin for Fig2G
55kD
40kD
AARS1 for Fig2H
130kD
100kD
YAP for Fig2H
100kD
70kD
AARS1 for Fig2H
130kD
100kD
YAP for Fig2H
70kD
β-actin for Fig2H
40kD
AARS1 for Fig2H
130kD
100kD
TEAD1 for Fig2H
70kD
55kD
AARS1 for Fig2H
130kD
100kD
TEAD1 for Fig2H
55kD
β-actin for Fig2H
40kD

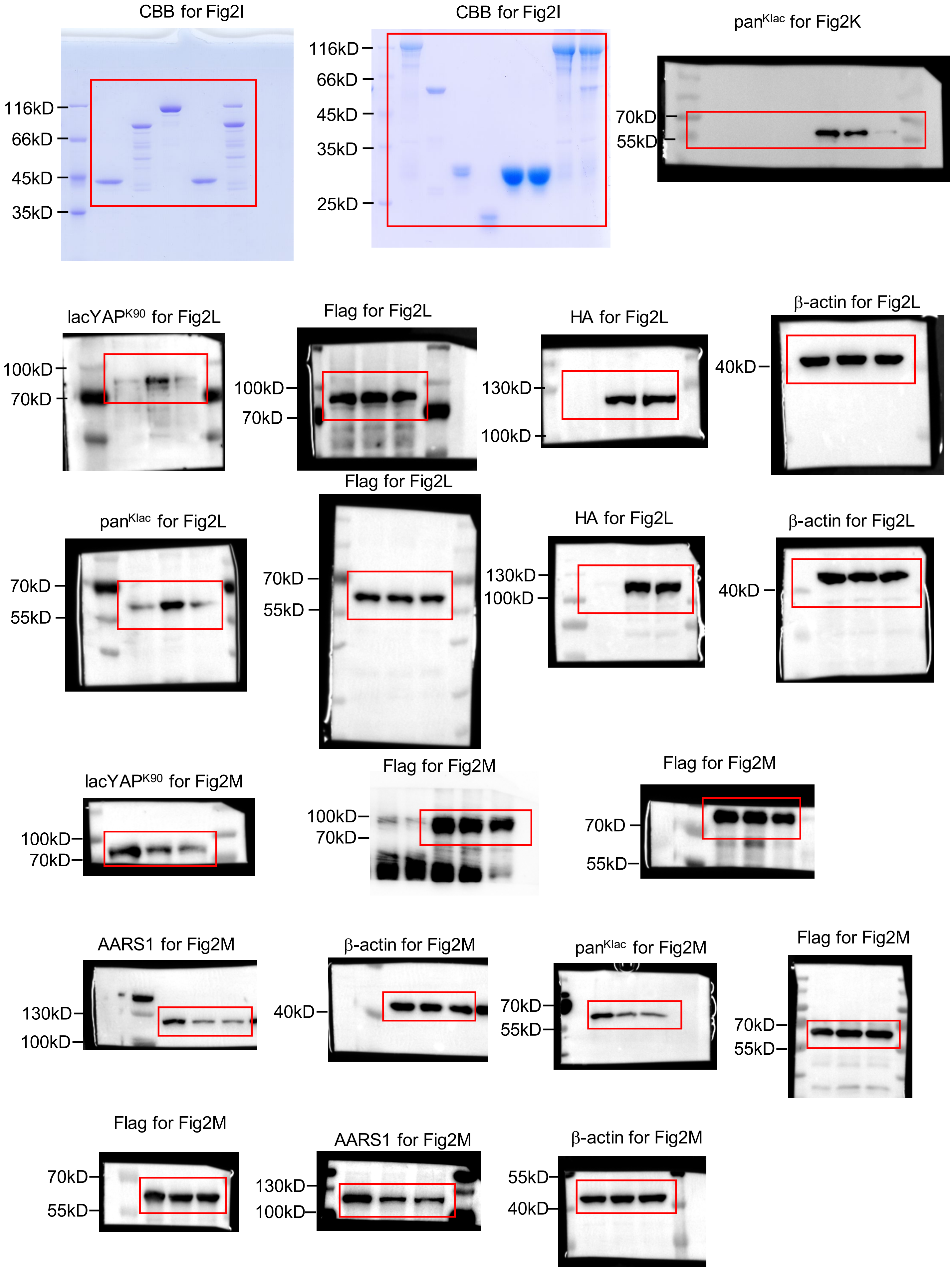
CBB for Fig2I
116kD
66kD
45kD
35kD
CBB for Fig2I
116kD
66kD
45kD
35kD
25kD
pan^Klac for Fig2K
70kD
55kD
lacYAP^K90 for Fig2L
100kD
70kD
Flag for Fig2L
100kD
70kD
HA for Fig2L
130kD
100kD
β-actin for Fig2L
40kD
pan^Klac for Fig2L
70kD
55kD
Flag for Fig2L
70kD
55kD
HA for Fig2L
130kD
100kD
β-actin for Fig2L
40kD
lacYAP^K90 for Fig2M
100kD
70kD
Flag for Fig2M
100kD
70kD
Flag for Fig2M
70kD
55kD
AARS1 for Fig2M
130kD
100kD
β-actin for Fig2M
40kD
pan^Klac for Fig2M
70kD
55kD
Flag for Fig2M
70kD
55kD
Flag for Fig2M
70kD
55kD
AARS1 for Fig2M
130kD
100kD
β-actin for Fig2M
55kD
40kD

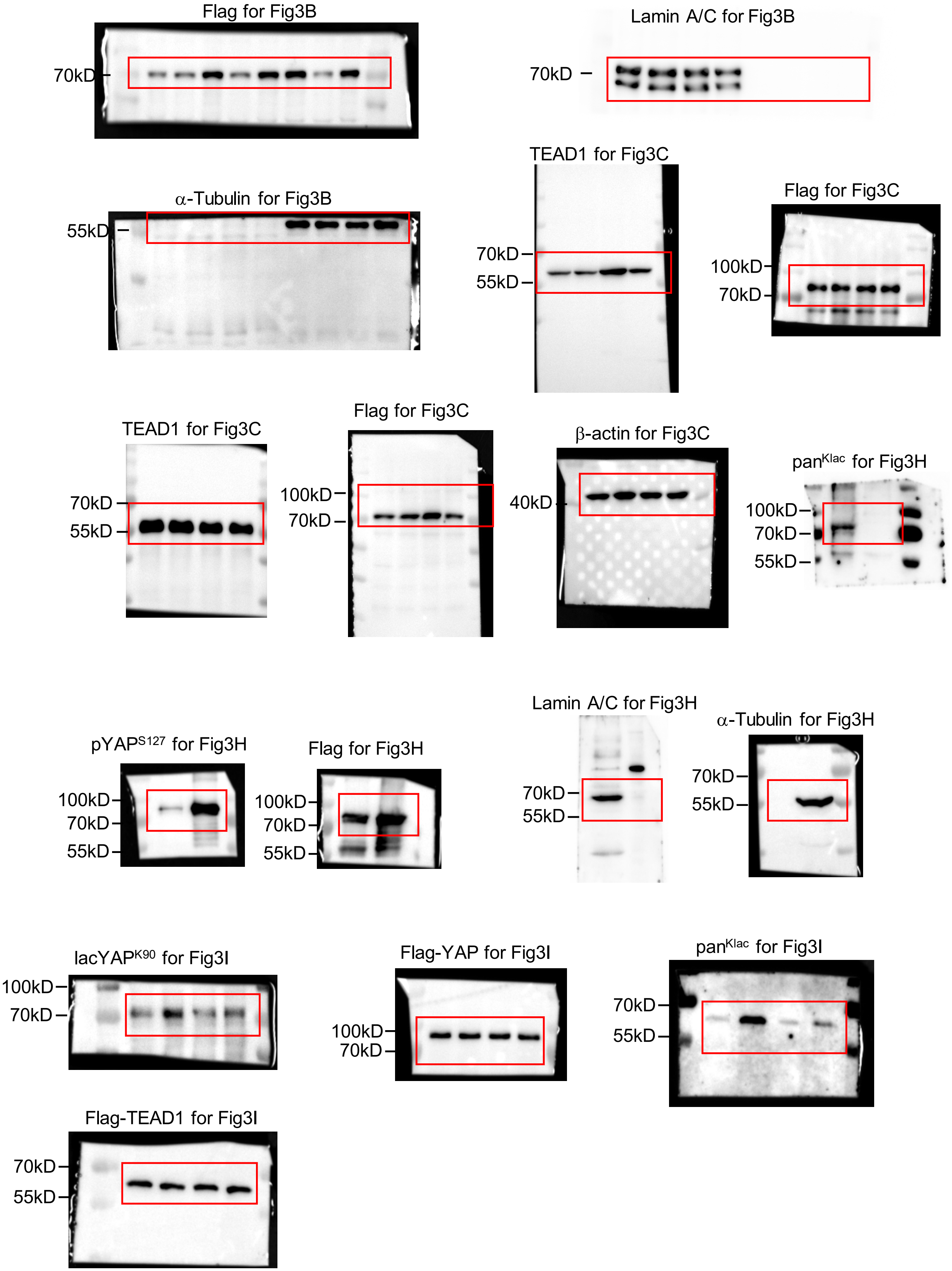

Flag for Fig3B
70kD
Lamin A/C for Fig3B
70kD
α-Tubulin for Fig3B
55kD
TEAD1 for Fig3C
70kD
55kD
Flag for Fig3C
100kD
70kD
TEAD1 for Fig3C
70kD
55kD
Flag for Fig3C
100kD
70kD
β-actin for Fig3C
40kD
panKlac for Fig3H
100kD
70kD
55kD
pYAPS127 for Fig3H
100kD
70kD
55kD
Flag for Fig3H
100kD
70kD
55kD
Lamin A/C for Fig3H
70kD
55kD
α-Tubulin for Fig3H
70kD
55kD
lacYAPK90 for Fig3I
100kD
70kD
Flag-YAP for Fig3I
100kD
70kD
panKlac for Fig3I
70kD
55kD
Flag-TEAD1 for Fig3I
70kD
55kD

Fig4D

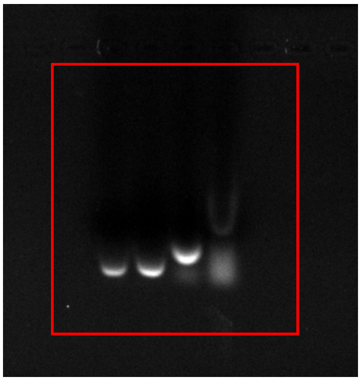

AARS1 for Fig4F

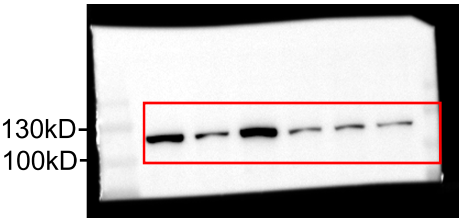

YAP for Fig4F

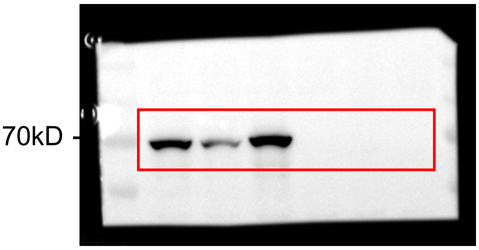

β-actin for Fig4F

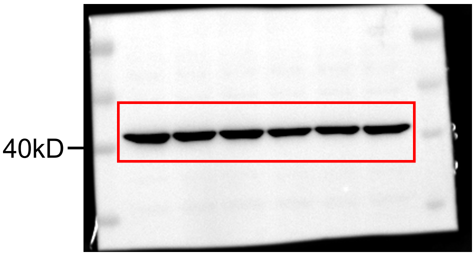

lacYAP^{K90} for Fig5B

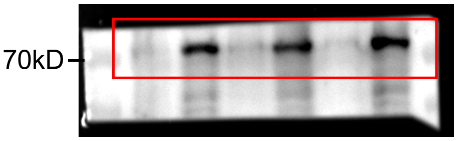

YAP for Fig5B

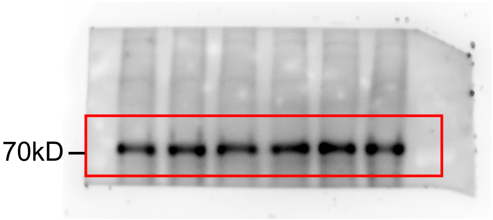

pan^{Klac} for Fig5B

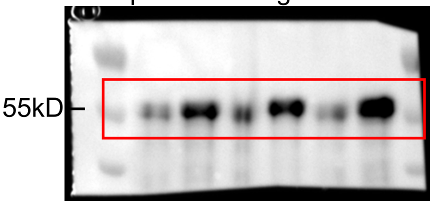

TEAD1 for Fig5B

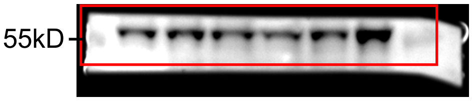

AARS1 for Fig5B

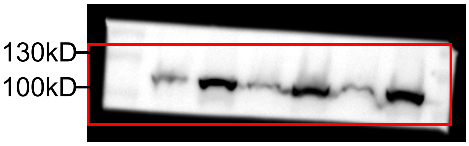

β-actin for Fig5B

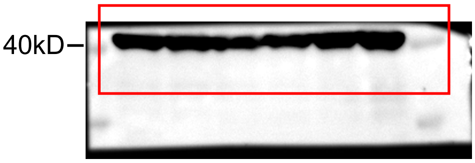

lacYAP^{K90} for Fig6D

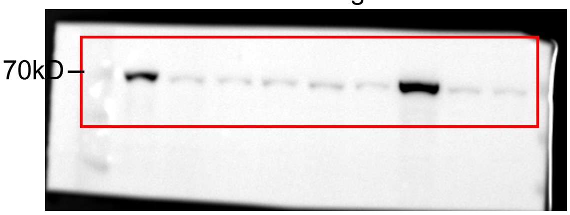

YAP for Fig6D

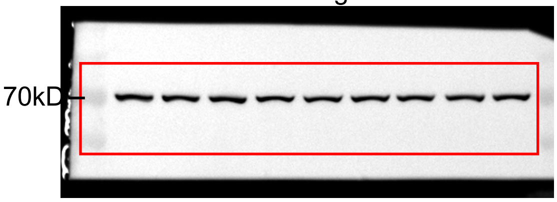

pan^{Klac} for Fig6D

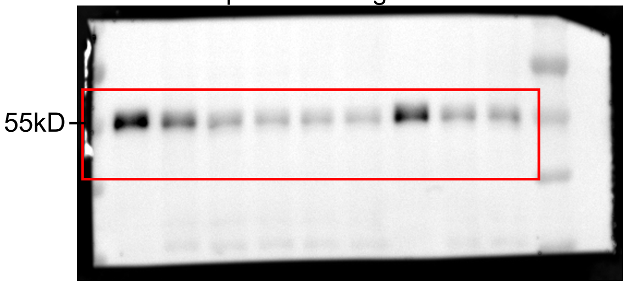

TEAD1 for Fig6D

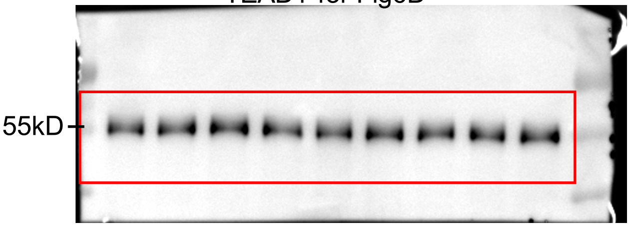

YAP for Fig6D

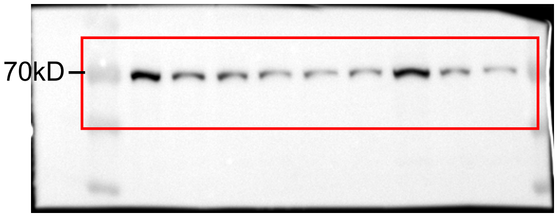

TEAD1 for Fig6D

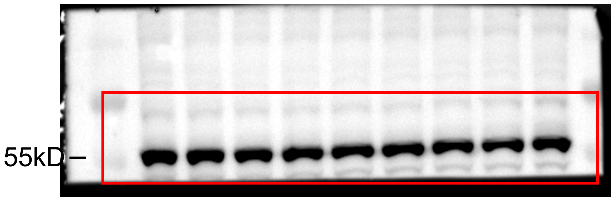

AARS1 for Fig6D

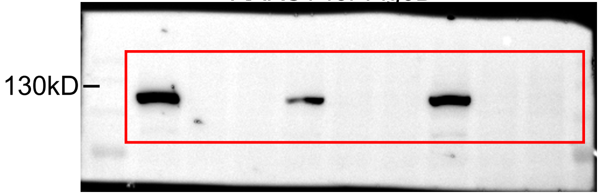

β-actin for Fig6D

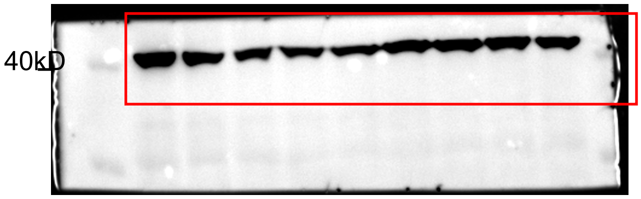

pan^{Klac} for Fig7C

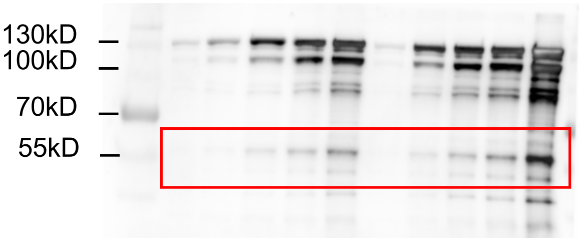

AARS1 for Fig7C

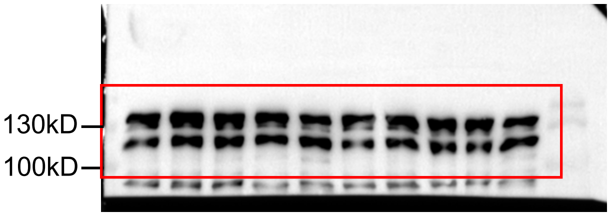

TEAD1 for Fig7C

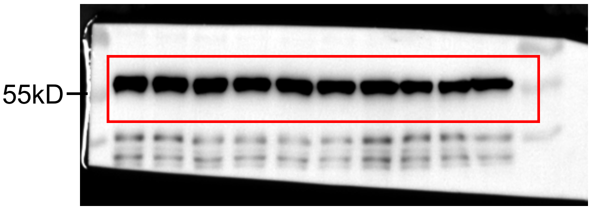

lacYAP^{K90} for Fig7D

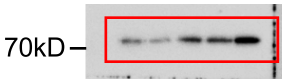

Flag for Fig7D

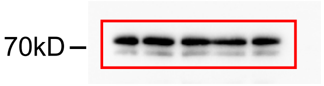

HA for Fig7D

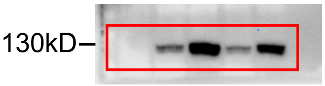

β-actin for Fig7D

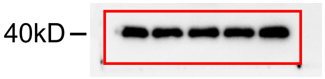

pan^{Klac} for Supplemental Fig1A

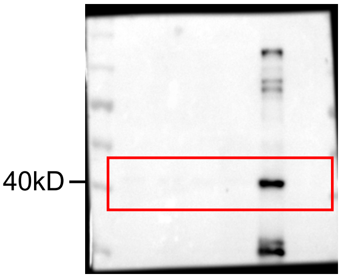

CBB for Supplemental Fig1A

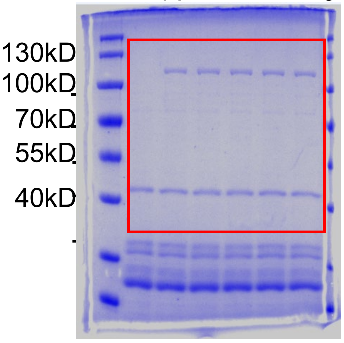

pan^{Klac} for Supplemental Fig1A

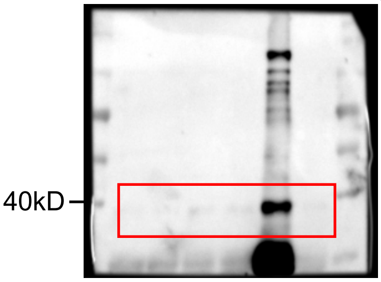

CBB for Supplemental Fig1A

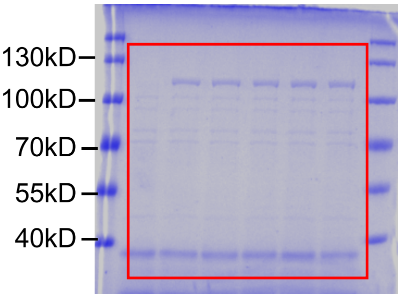

CBB for Supplemental Fig1D

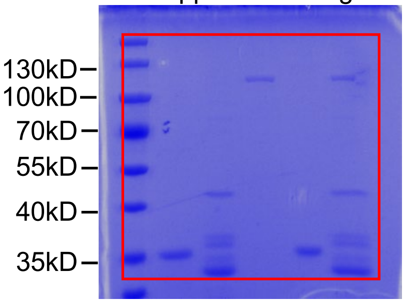

CBB for Supplemental Fig1D

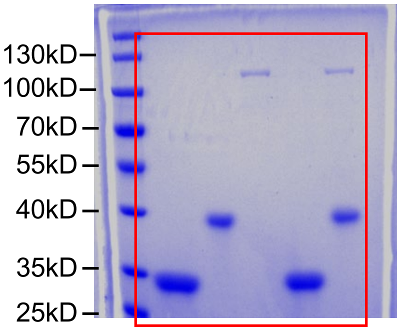

pan^{Klac} for Supplemental Fig1F

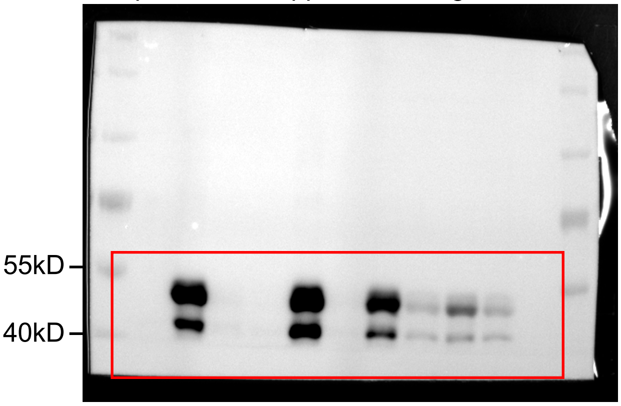

CBB for Supplemental Fig1F

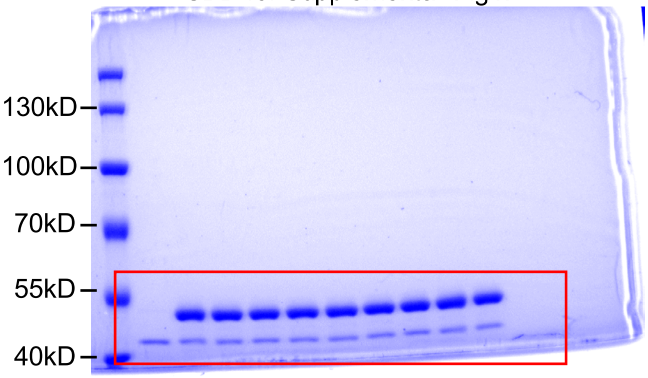

pan^{Klac} for Supplemental Fig1G

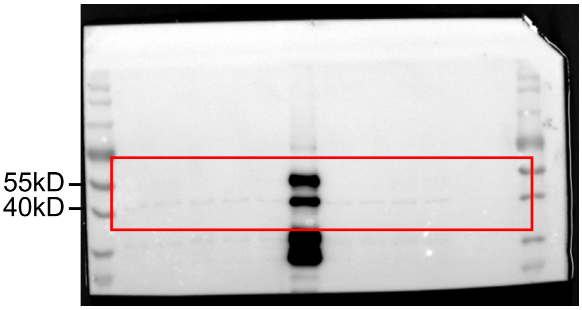

CBB for Supplemental Fig1G

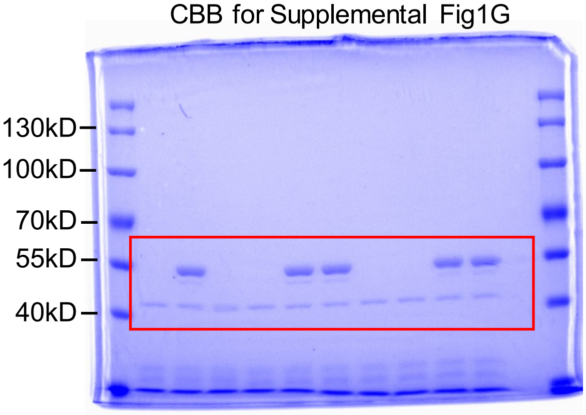

pan^{Klac} for Supplemental Fig1H

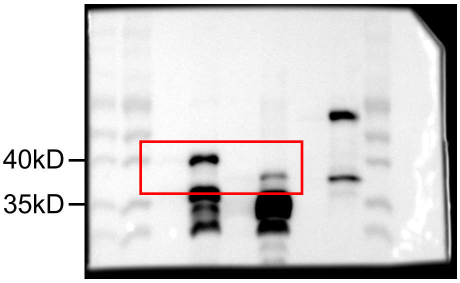

CBB for Supplemental Fig1H

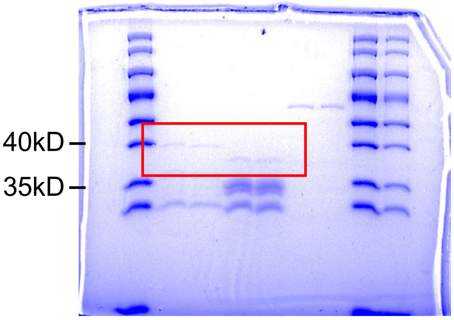

H3K18^{lac} for Supplemental Fig1I

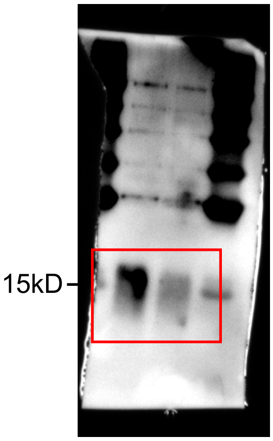

H3 for Supplemental Fig1I

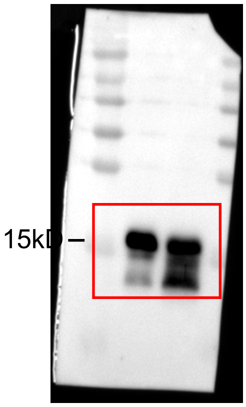

AARS1 for Supplemental Fig1I

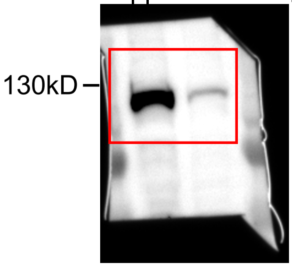

β-actin for Supplemental Fig1I

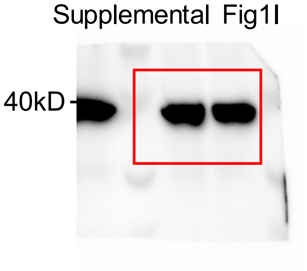

pan^{Klac} for Supplemental Fig1J

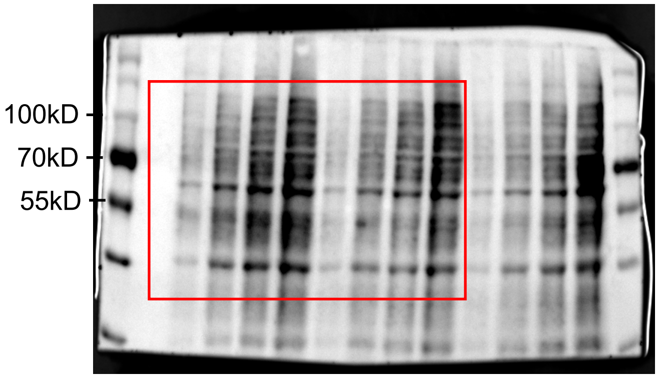

p300 for Supplemental Fig1J

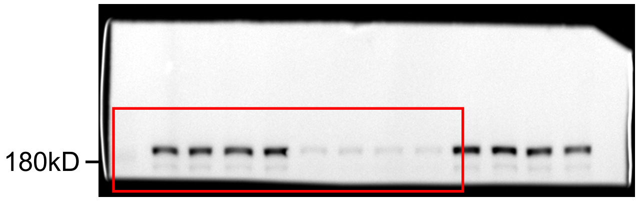

β-actin for Supplemental Fig1J

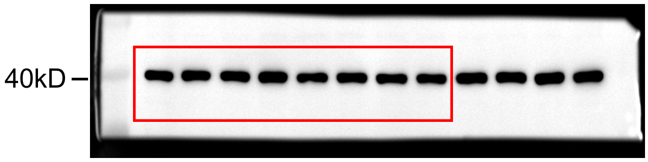

HA for Supplemental Fig1L

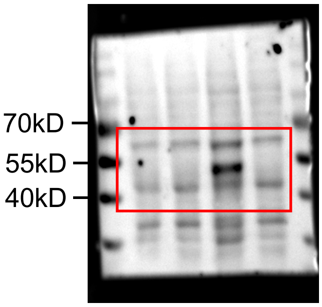

Flag for Supplemental Fig1L

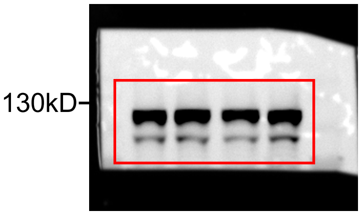

HA for Supplemental Fig1L

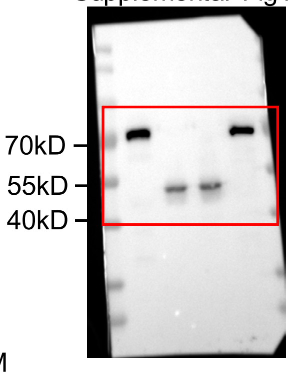

Flag for Supplemental Fig1L

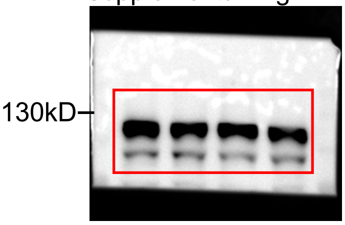

β-actin for Supplemental Fig1L

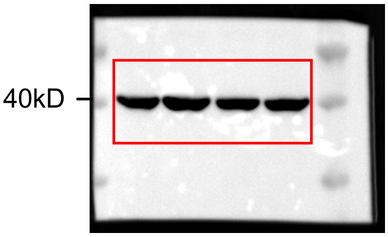

HA for Supplemental Fig1M

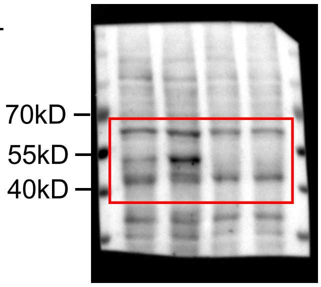

Flag for Supplemental Fig1M

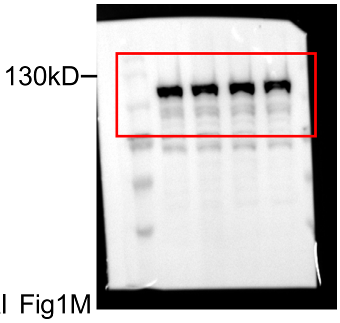

HA for Supplemental Fig1M

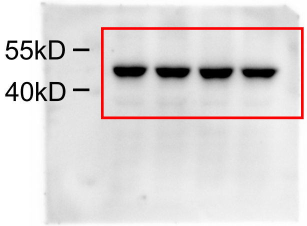

Flag for Supplemental Fig1M

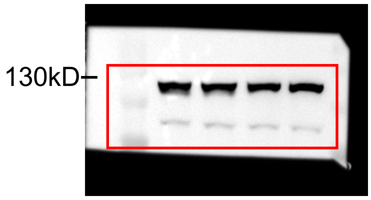

β-actin for Supplemental Fig1M

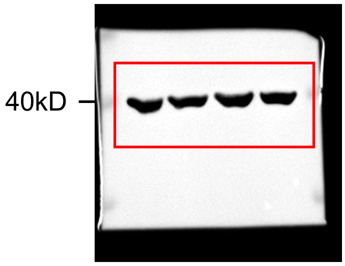

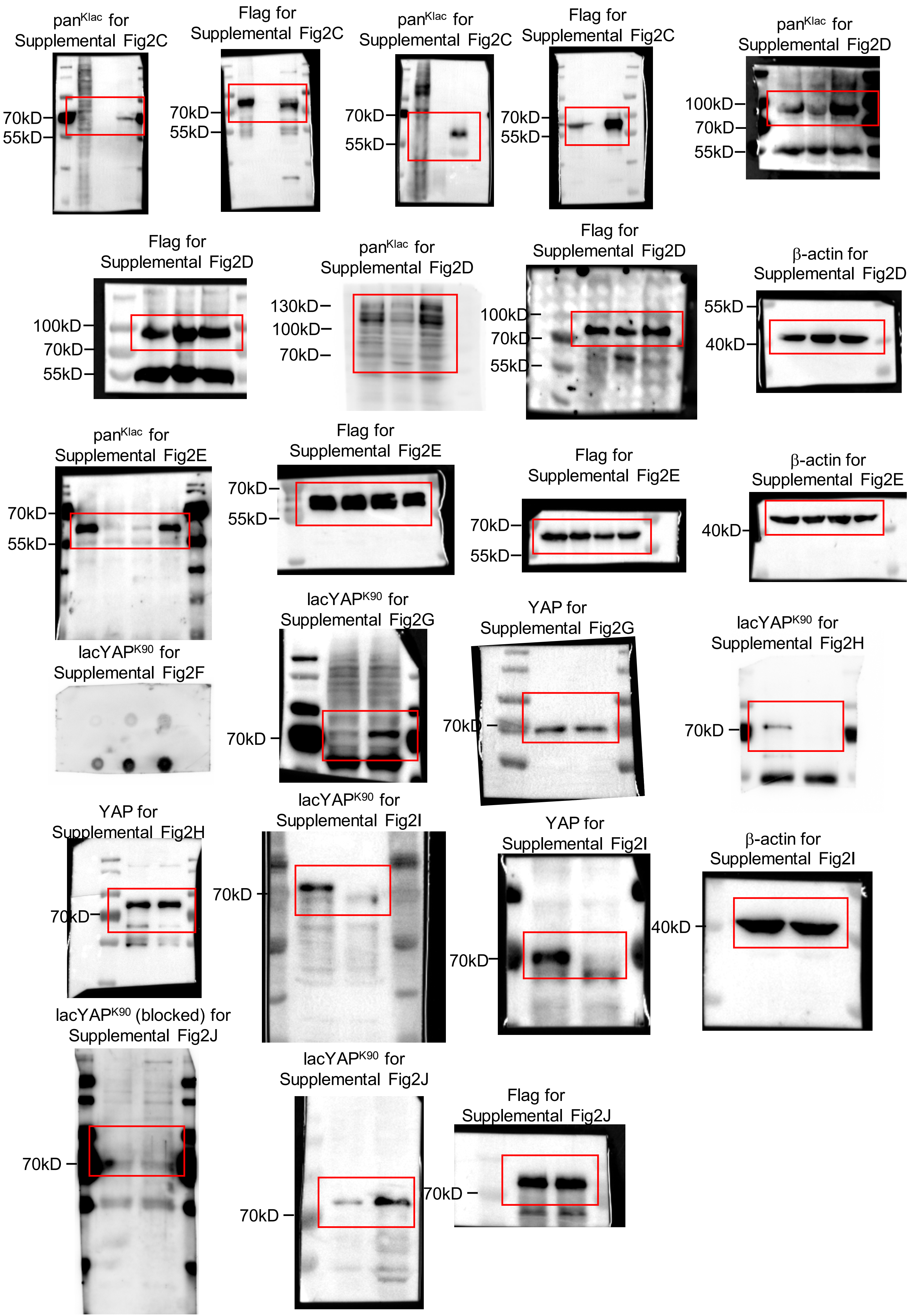
pan^{Klac} for Supplemental Fig2C
70kD
55kD
Flag for Supplemental Fig2C
70kD
55kD
pan^{Klac} for Supplemental Fig2C
70kD
55kD
Flag for Supplemental Fig2C
70kD
55kD
pan^{Klac} for Supplemental Fig2D
100kD
70kD
55kD
Flag for Supplemental Fig2D
100kD
70kD
55kD
pan^{Klac} for Supplemental Fig2D
130kD
100kD
70kD
Flag for Supplemental Fig2D
100kD
70kD
55kD
β-actin for Supplemental Fig2D
55kD
40kD
pan^{Klac} for Supplemental Fig2E
70kD
55kD
Flag for Supplemental Fig2E
70kD
55kD
Flag for Supplemental Fig2E
70kD
55kD
β-actin for Supplemental Fig2E
40kD
lacYAP^{K90} for Supplemental Fig2F
lacYAP^{K90} for Supplemental Fig2G
70kD
YAP for Supplemental Fig2G
70kD
lacYAP^{K90} for Supplemental Fig2H
70kD
YAP for Supplemental Fig2H
70kD
lacYAP^{K90} for Supplemental Fig2I
70kD
YAP for Supplemental Fig2I
70kD
β-actin for Supplemental Fig2I
40kD
lacYAP^{K90} (blocked) for Supplemental Fig2J
70kD
lacYAP^{K90} for Supplemental Fig2J
70kD
Flag for Supplemental Fig2J
70kD

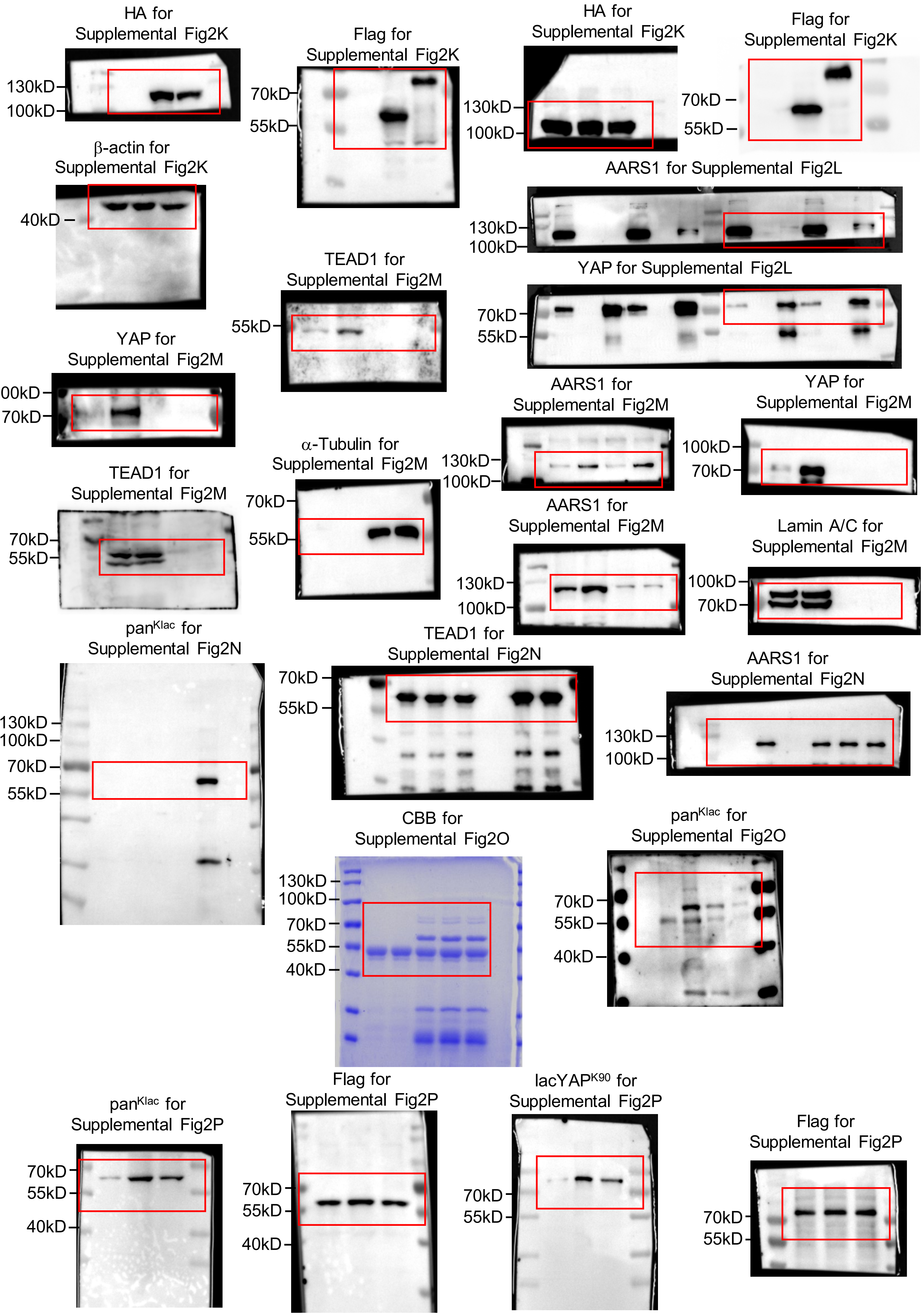
HA for Supplemental Fig2K
130kD
100kD
Flag for Supplemental Fig2K
70kD
55kD
HA for Supplemental Fig2K
130kD
100kD
Flag for Supplemental Fig2K
70kD
55kD
β-actin for Supplemental Fig2K
40kD
AARS1 for Supplemental Fig2L
130kD
100kD
TEAD1 for Supplemental Fig2M
55kD
YAP for Supplemental Fig2L
70kD
55kD
YAP for Supplemental Fig2M
00kD
70kD
AARS1 for Supplemental Fig2M
130kD
100kD
YAP for Supplemental Fig2M
100kD
70kD
α-Tubulin for Supplemental Fig2M
70kD
55kD
TEAD1 for Supplemental Fig2M
70kD
55kD
AARS1 for Supplemental Fig2M
130kD
100kD
Lamin A/C for Supplemental Fig2M
100kD
70kD
panKlac for Supplemental Fig2N
130kD
100kD
70kD
55kD
TEAD1 for Supplemental Fig2N
70kD
55kD
AARS1 for Supplemental Fig2N
130kD
100kD
CBB for Supplemental Fig2O
130kD
100kD
70kD
55kD
40kD
panKlac for Supplemental Fig2O
70kD
55kD
40kD
panKlac for Supplemental Fig2P
70kD
55kD
40kD
Flag for Supplemental Fig2P
70kD
55kD
40kD
lacYAPK90 for Supplemental Fig2P
70kD
55kD
Flag for Supplemental Fig2P
70kD
55kD

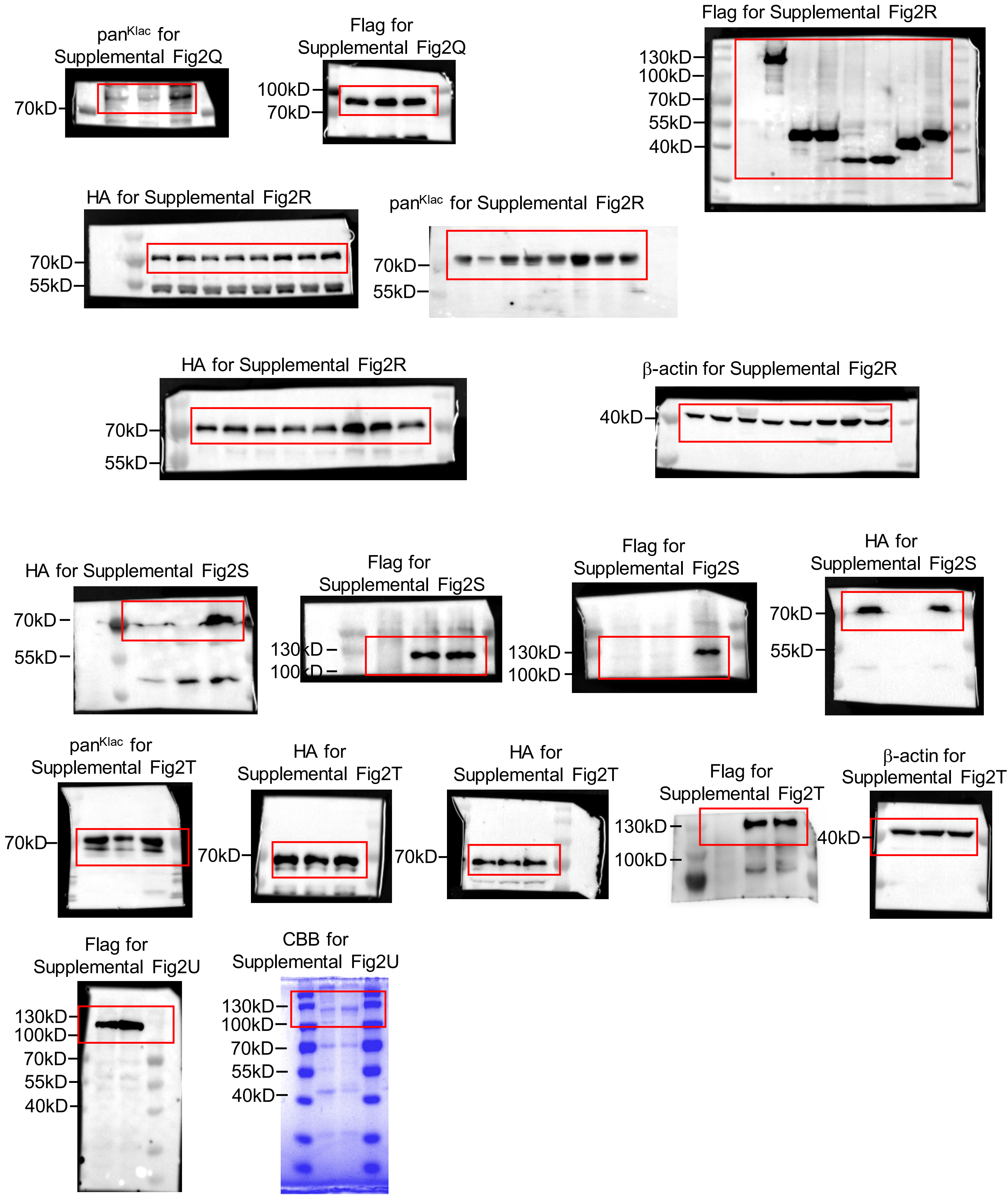

pan^{Klac} for Supplemental Fig2Q
70kD
Flag for Supplemental Fig2Q
100kD
70kD
Flag for Supplemental Fig2R
130kD
100kD
70kD
55kD
40kD
HA for Supplemental Fig2R
70kD
55kD
pan^{Klac} for Supplemental Fig2R
70kD
55kD
HA for Supplemental Fig2R
70kD
55kD
β-actin for Supplemental Fig2R
40kD
HA for Supplemental Fig2S
70kD
55kD
Flag for Supplemental Fig2S
130kD
100kD
Flag for Supplemental Fig2S
130kD
100kD
HA for Supplemental Fig2S
70kD
55kD
pan^{Klac} for Supplemental Fig2T
70kD
HA for Supplemental Fig2T
70kD
HA for Supplemental Fig2T
70kD
Flag for Supplemental Fig2T
130kD
100kD
β-actin for Supplemental Fig2T
40kD
Flag for Supplemental Fig2U
130kD
100kD
70kD
55kD
40kD
CBB for Supplemental Fig2U
130kD
100kD
70kD
55kD
40kD

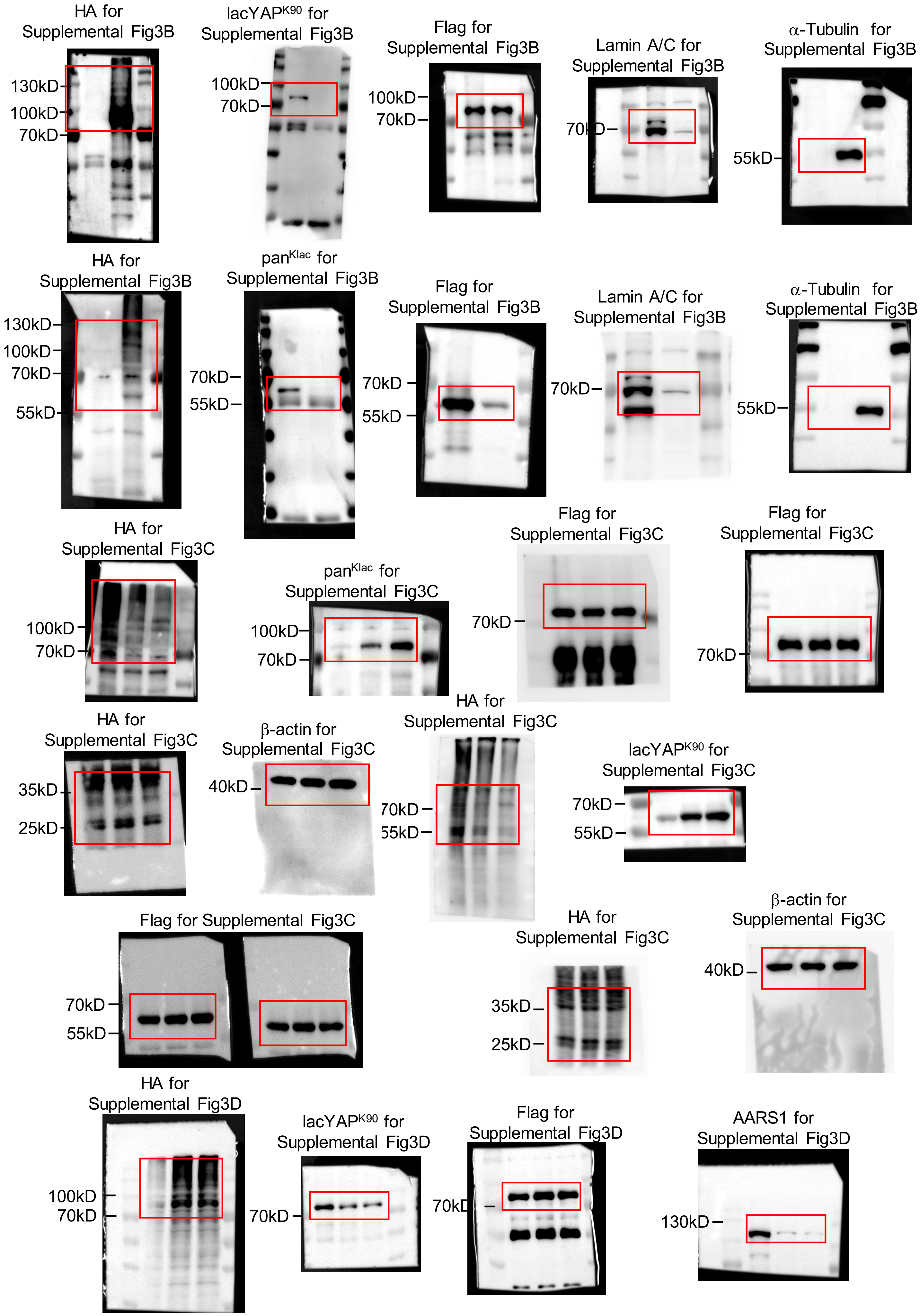
HA for Supplemental Fig3B
130kD
100kD
70kD
lacYAP^{K90} for Supplemental Fig3B
100kD
70kD
Flag for Supplemental Fig3B
100kD
70kD
Lamin A/C for Supplemental Fig3B
70kD
α-Tubulin for Supplemental Fig3B
55kD
HA for Supplemental Fig3B
130kD
100kD
70kD
55kD
pan^{Klac} for Supplemental Fig3B
70kD
55kD
Flag for Supplemental Fig3B
70kD
55kD
Lamin A/C for Supplemental Fig3B
70kD
α-Tubulin for Supplemental Fig3B
55kD
HA for Supplemental Fig3C
100kD
70kD
pan^{Klac} for Supplemental Fig3C
100kD
70kD
Flag for Supplemental Fig3C
70kD
Flag for Supplemental Fig3C
70kD
HA for Supplemental Fig3C
35kD
25kD
β-actin for Supplemental Fig3C
40kD
HA for Supplemental Fig3C
70kD
55kD
lacYAP^{K90} for Supplemental Fig3C
70kD
55kD
Flag for Supplemental Fig3C
70kD
55kD
HA for Supplemental Fig3C
35kD
25kD
β-actin for Supplemental Fig3C
40kD
HA for Supplemental Fig3D
100kD
70kD
lacYAP^{K90} for Supplemental Fig3D
70kD
Flag for Supplemental Fig3D
70kD
AARS1 for Supplemental Fig3D
130kD

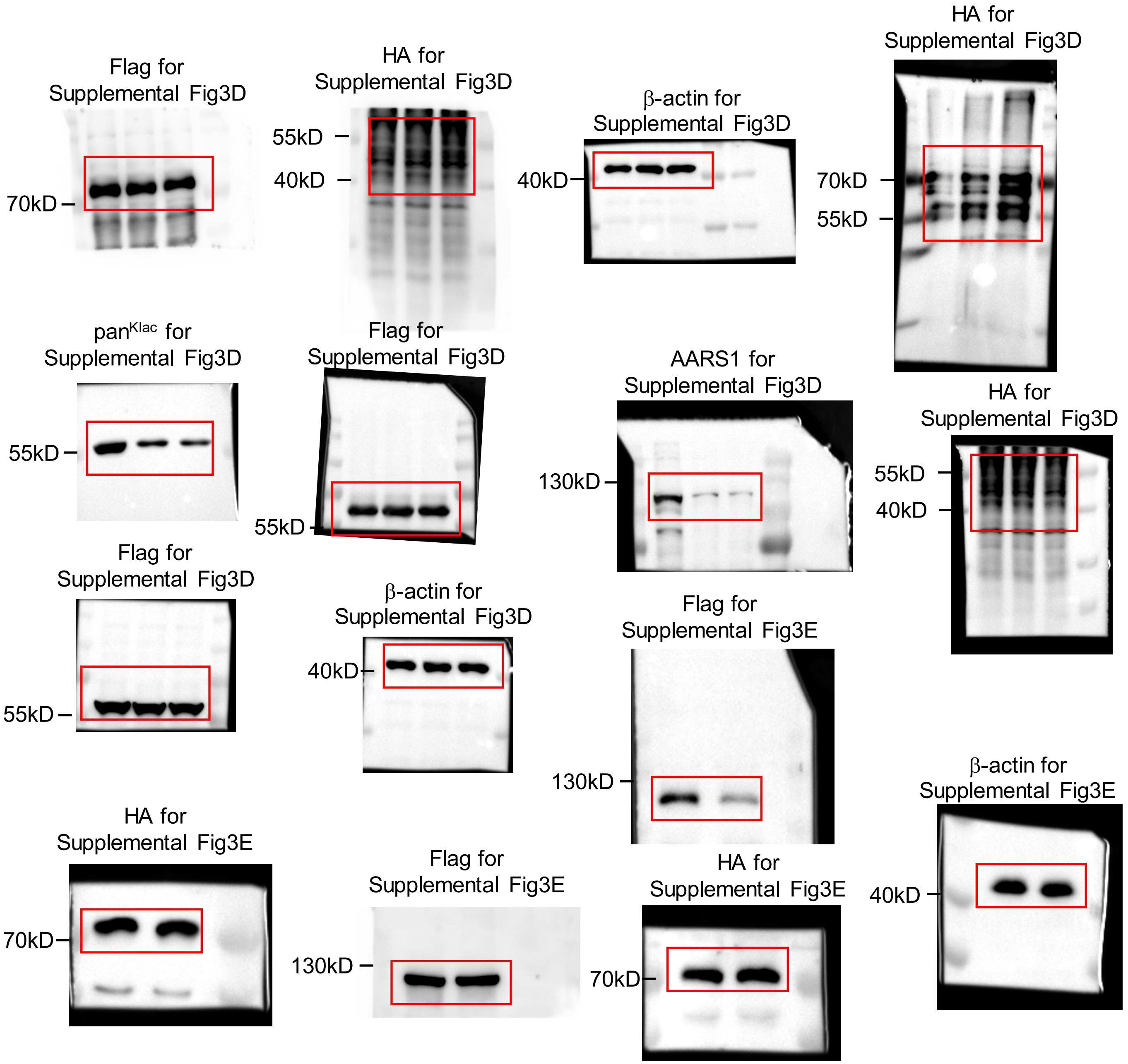

Flag for
Supplemental Fig3D
70kD
HA for
Supplemental Fig3D
55kD
40kD
β-actin for
Supplemental Fig3D
40kD
HA for
Supplemental Fig3D
70kD
55kD
pan^{Klac} for
Supplemental Fig3D
55kD
Flag for
Supplemental Fig3D
55kD
AARS1 for
Supplemental Fig3D
130kD
HA for
Supplemental Fig3D
55kD
40kD
Flag for
Supplemental Fig3D
55kD
β-actin for
Supplemental Fig3D
40kD
Flag for
Supplemental Fig3E
130kD
β-actin for
Supplemental Fig3E
40kD
HA for
Supplemental Fig3E
70kD
Flag for
Supplemental Fig3E
130kD
HA for
Supplemental Fig3E
70kD

Flag for
Supplemental Fig 4C

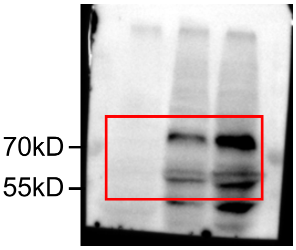

β-actin for
Supplemental Fig 4C

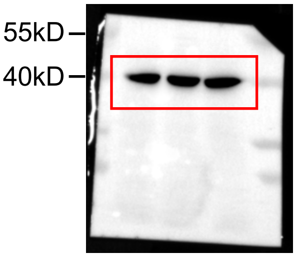

AARS1 for Supplemental Fig 5C

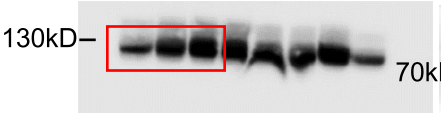

YAP for Supplemental Fig 5C

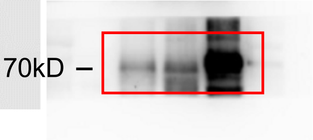

TEAD1 for Supplemental Fig 5C

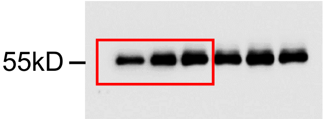

β-actin for Supplemental Fig 5C

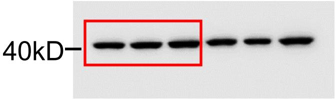

AARS1 for Supplemental Fig 5B

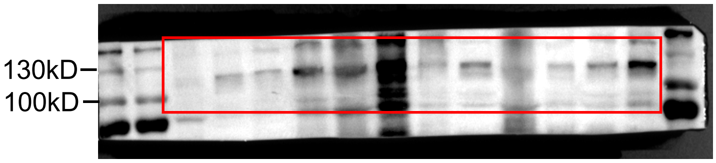

β-actin for Supplemental Fig 5B

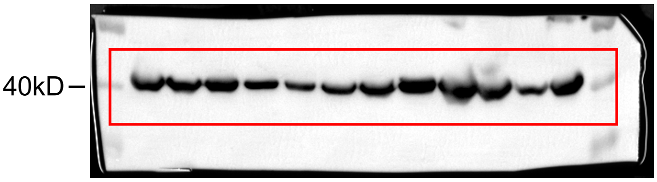

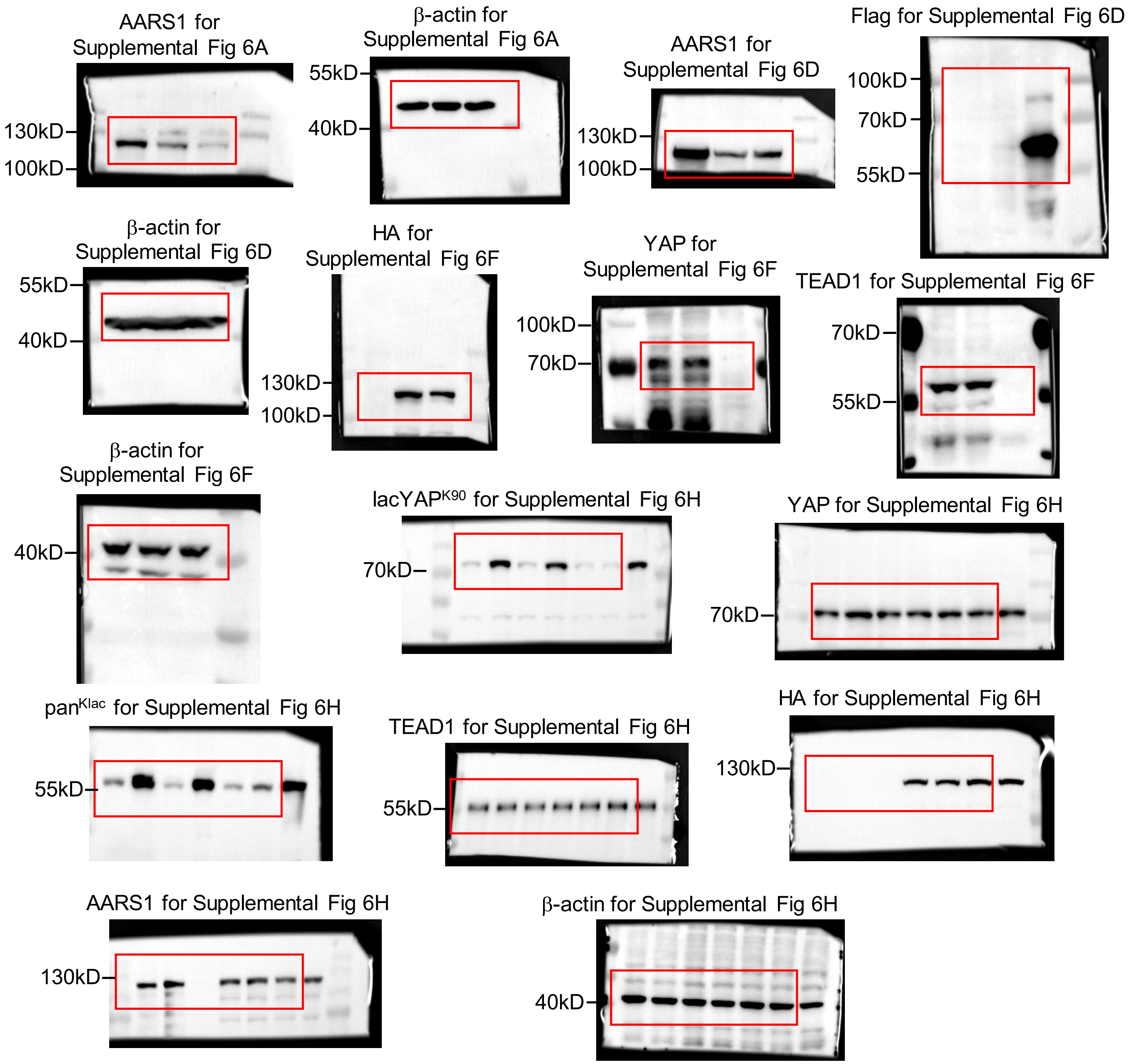

AARS1 for Supplemental Fig 6A
130kD
100kD
β-actin for Supplemental Fig 6A
55kD
40kD
AARS1 for Supplemental Fig 6D
130kD
100kD
Flag for Supplemental Fig 6D
100kD
70kD
55kD
β-actin for Supplemental Fig 6D
55kD
40kD
HA for Supplemental Fig 6F
130kD
100kD
YAP for Supplemental Fig 6F
100kD
70kD
TEAD1 for Supplemental Fig 6F
70kD
55kD
β-actin for Supplemental Fig 6F
40kD
lacYAP^{K90} for Supplemental Fig 6H
70kD
YAP for Supplemental Fig 6H
70kD
pan^{Klac} for Supplemental Fig 6H
55kD
TEAD1 for Supplemental Fig 6H
55kD
HA for Supplemental Fig 6H
130kD
AARS1 for Supplemental Fig 6H
130kD
β-actin for Supplemental Fig 6H
40kD

HA for Supplemental Fig7

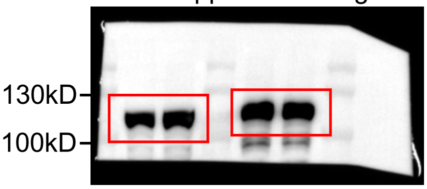

Flag for Supplemental Fig7

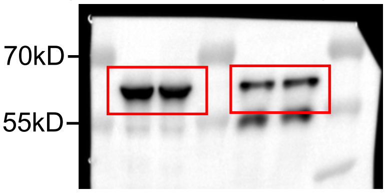

β-actin for Supplemental Fig7

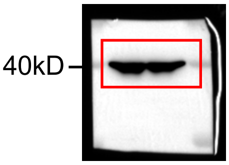

HA for Supplemental Fig7

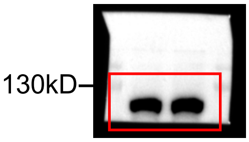

Flag for Supplemental Fig7

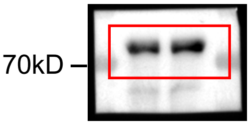

HA for Supplemental Fig7

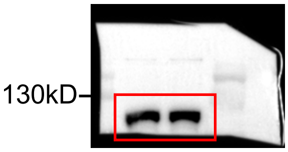

Flag for Supplemental Fig7

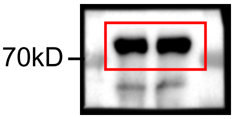

β-actin for Supplemental Fig7

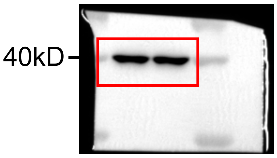

Supplement: Unedited blot and gel images [file jci-134-174587-s263.pdf]
